# Endosteal integrin-α8⁺ mesenchymal stem cells maintain haematopoietic stem cell function via extracellular matrix-mediated interactions

**DOI:** 10.1038/s41467-026-75276-2

**Published:** 2026-07-06

**Authors:** Ryosuke Yuta, Hisayuki Yao, Kentaro Hosokawa, Yuichiro Semba, Yuki Esaki, Shunichi Adachi, Ngan Thi Kim Nguyen, Ben D MacArthur, Fumio Arai

**Affiliations:** 1 Department of Stem Cell Biology and Medicine, Graduate School of Medical Sciences, Kyushu University, Fukuoka, Japan; 2 Department of Medicine and Biosystemic Science, Kyushu University Graduate School of Medical Sciences, Fukuoka, Japan; 3 Division of Precision Medicine, Kyushu University Graduate School of Medical Sciences, Fukuoka, Japan; 4 The Alan Turing Institute, London, UK; 5 Institute for Life Sciences, University of Southampton, Southampton, UK; 6 Mathematical Sciences, University of Southampton, Southampton, UK

**Keywords:** Haematopoietic stem cells, Mesenchymal stem cells, Stem-cell niche

## Abstract

The stem cell niche is a specialised microenvironment essential for maintaining haematopoietic stem cells (HSCs). Here we identify a distinct subset of mesenchymal stem cells (MSCs) expressing integrin α8 (Itga8⁺ MSCs) within the bone marrow (BM) endosteum. These cells exhibit distinct MSC properties and higher haematopoietic supportive activity than other MSC subsets. Depletion of Itga8⁺ MSCs decreased HSC numbers, reduced quiescence of endosteal HSCs and diminished BM repopulating capacity. Itga8⁺ MSCs support haematopoiesis through cell adhesion-related mechanisms. Single-cell RNA sequencing further identified Itga8⁺ MSCs as a distinct subpopulation within bone-lining cells. Moreover, we identified Mfap4 as a candidate factor expressed in Itga8⁺ MSCs, and Mfap4 supported the BM reconstitution capacity of HSCs. These results suggest that Itga8⁺ MSCs represent a distinct niche cell population for HSC maintenance. This work provides new insights into HSC regulation and strategies to optimise in vitro HSC maintenance and enhance in vivo engraftment.

## Abstract (web summary)

Here they identify integrin α8⁺ mesenchymal stem cells as a distinct endosteal bone marrow niche population that supports HSC maintenance. Their depletion impairs HSC quiescence and repopulation, with Mfap4 identified as one of the supportive factors.

## Introduction

Haematopoietic stem cells (HSCs) are essential for the continuous production of all blood cell lineages and the maintenance of haematological homeostasis through their self-renewal and differentiation capabilities^1,2^. The function of HSCs is critically dependent on their surrounding microenvironment, known as the HSC niche^3–5^, which provides structural support and essential signals^6–10^. The HSC niche can be broadly divided into two anatomical regions: the perivascular and endosteal niche^11–13^. Within these areas, various factors like C-X-C motif chemokine ligand 12 (CXCL12), stem cell factor (SCF) and angiopoietin-1 (Angpt1) are crucial for HSC maintenance^6–8^.

In the perivascular niche, vascular endothelial cells, mesenchymal stem cells (MSCs), and CXCL12-abundant reticular (CAR) cells are essential for HSC support^6,14,15^. Perivascular MSCs, identified by markers such as leptin receptor-positive (LepR⁺) and Nestin-GFP⁺ contribute significantly to HSC maintenance^14,16^. Mature haematopoietic cells, including megakaryocytes and macrophages, also influence HSC maintenance within this microenvironment^17–19^.

The endosteal niche, adjacent to the bone surface, comprises mesenchymal stem/progenitor cells (MSC/MPCs), osteoblasts and osteoblast progenitors^13,20,21^. This region is often associated with quiescent HSCs^8,9,22^, and HSC numbers correlate with endosteal niche cell abundance^13,21^. Although some studies suggest the endosteal area is more important for lymphoid progenitor cells, our previous studies indicate it also plays a direct role in HSC maintenance^23–25^. We previously identified three bone-lining cell (BLC) populations in the endosteal niche: Activated leukocyte cell adhesion molecule (Alcam)⁺ Stem cell antigen-1 (Sca-1)^−^ osteoblasts, Alcam^−^Sca-1⁺ MSCs and Alcam^−^Sca-1^−^ cells^26^. Among these, Alcam⁺Sca-1⁻ BLCs express high levels of cell adhesion-related molecules and maintain the long-term BM reconstitution capacity of HSCs in vitro^26^. These findings suggest that Alcam⁺Sca-1^−^ BLCs are a key component of the endosteal niche, providing physical support through cell-to-cell and cell-to-extracellular matrix (ECM) interactions that preserve HSC function.

Within the Alcam⁺Sca-1^−^ BLC population, we previously identified a subpopulation expressing pluripotent stem cell marker genes and low levels of osteoblast markers^26^, although the nature and function of the cells in this fraction were unclear because methods for their selective isolation were not available. In this study, we show that this subpopulation is characterised by expression of integrin α8 (*Itga8*). The Itga8⁺ fraction shows osteogenic, adipogenic, and chondrogenic differentiation capacity, indicating that it represents bona fide MSCs. Furthermore, we demonstrate that Itga8⁺ MSCs play a crucial role in supporting HSC quiescence and maintaining long-term repopulating (LTR) capacity.

In this study, we identify Itga8⁺ MSCs as a distinct MSC subpopulation in the bone marrow endosteum with superior haematopoietic supportive activity. Depletion of Itga8⁺ MSCs reduces HSC numbers, impairs endosteal HSC quiescence, and diminishes BM repopulating capacity, demonstrating that these cells are essential components of the endosteal niche. Furthermore, we identify Mfap4 as a candidate niche factor expressed by Itga8⁺ MSCs that supports the BM reconstitution capacity of HSCs. These findings provide new insights into HSC regulation and offer potential strategies for optimising in vitro HSC maintenance and enhancing in vivo engraftment for therapeutic applications.

## Results

### Identification of an Itga8+ BLC subset in the endosteal BM

**Fig. 1:**
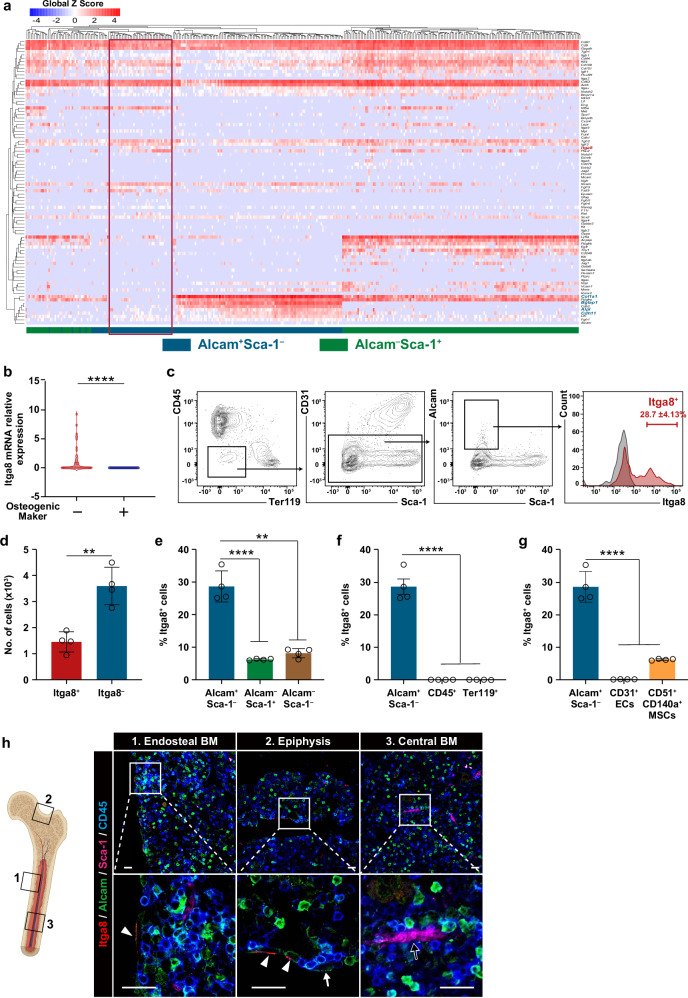
An Itga8^+^ subpopulation is present within the Alcam^+^Sca-1^−^ BLC population. **a** Heat map of differentially expressed genes in Alcam^+^Sca-1^−^ BLCs and Alcam^−^Sca-1^+^ BLCs from single-cell qPCR. A subpopulation within Alcam^+^Sca-1^−^ BLCs that does not express osteogenic markers (Red frame) characteristically expresses Itga8. Alcam^+^Sca-1^−^ BLCs (192 cells, blue), Alcam^−^Sca-1^+^ BLCs (192 cells, green). *Itga8* (Red), Osteoblast markers: *Col1a1*, *Bglap1*, *Alpl* and *Cdh11* (Blue). **b** Comparison of *Itga8* expression between Alcam^+^Sca-1^−^ BLCs lacking osteoblast markers and Alcam^+^Sca-1^−^ BLCs expressing osteoblast markers. *Itga8* expression was analysed using single-cell qPCR shown in (**a**). **c** Representative flow cytometry profile of BLCs stained with CD45, Ter119, CD31, Alcam, Sca-1 and Itga8. **d** The number of Itga8^+^Alcam^+^Sca-1^−^ BLCs (Itga8^+^) and Itga8^−^Alcam^+^Sca-1^−^ BLCs (Itga8^−^) in mice (*n* = 4/group). **e** The proportion of Itga8^+^ cells in Alcam^+^Sca-1^−^ BLCs, Alcam^−^Sca-1^+^ MSCs and Alcam^−^Sca-1^−^ BLCs. **f** Itga8 expression in CD45^+^ haematopoietic cells and Ter119^+^ erythroid cells. **g** Itga8 expression in CD31^+^ endothelial cells and CD51^+^CD140a^+^ MSCs of the vascular niche. (*n* = 4/group). **h** Immunohistochemical staining of the BM in the femur for Itga8 (red), Alcam (green), Sca-1 (magenta) and CD45 (blue). (1): Endosteal region, (2): Epiphysis, (3): Central BM. The lower panels are magnified views of the area outlined by the box in the upper panel. Itga8^+^Alcam^+^Sca-1^−^ BLCs (arrowheads), Itga8^−^Alcam^+^Sca-1^−^ BLCs (arrows) and Alcam^−^Sca-1^+^ BLCs (open arrowheads). Scale bars: 20 μm. Data are presented as mean ± SD. Statistical significance was determined by a two-sided unpaired *t*-test for (**b** and **d**). Statistical differences were determined by ordinary one-way ANOVA followed by Tukey’s multiple-comparison test for (**e–g**).**p < 0.01, ****p < 0.0001 (**b**) exact *p* = 6.77 × 10⁻⁸. **d** exact *p* = 0.0019. **e** Alcam^+^Sca-1^−^ vs Alcam^−^Sca-1^+^: exact *p* = 4.2 × 10^−^^6^. Alcam^+^Sca-1^−^ vs Alcam^−^Sca-1^−^: exact *p* = 8.8 × 10^−^^6^. **f** Alcam^+^Sca-1^−^ vs CD45^+^: exact *p* = 3.7 × 10^−^^7^. Alcam^+^Sca-1^−^ vs Ter119^+^: exact *p* = 3.7 × 10^−^^7^. **g** Alcam^+^Sca-1^−^ vs CD31^+^ECs: exact *p* = 3.8 × 10^−^^7^. Alcam^+^Sca-1^−^ vs CD51^+^CD140a^+^MSCs: exact *p* = 2.9 × 10^−^^6^. Created in BioRender. Arai, F. (2026) https://BioRender.com/weipum5.

Alcam^+^Sca-1^−^ BLCs contain a unique subpopulation that does not express osteoblast markers^26^. To identify this subpopulation within Alcam^+^Sca-1^−^ BLCs, we performed single-cell quantitative polymerase chain reaction (qPCR) analysis of Alcam^+^Sca-1^−^ BLCs and Alcam^−^Sca-1^+^ BLCs. After single-cell RNA amplification, multi-gene expression profiling was conducted using a high-density microfluidic device to characterise the subpopulations in detail (Fig. 1a). Many Alcam^+^Sca-1^**−**^ BLCs expressed osteoblast markers such as alkaline phosphatase (*Alpl*), osteocalcin (*Ocn*), collagen type 1a1 (*Col1a1*) and cadherin-11 (*Cdh11*) (Fig. 1a). However, consistent with previous reports, we identified a subpopulation that did not express these markers (Fig. 1a; red frame)^26^. Furthermore, we found that this subpopulation characteristically expressed *Itga8* (Fig. 1a; red frame and Extended Data Fig. 1).

Itga8^+^ cells were rarely detected within the osteoblast-marker-positive fraction (Fig. 1b and Extended Data Fig. 1). Flow cytometric analysis showed that 28.7 ± 4.13% of the Alcam^+^Sca-1^−^ BLCs express Itga8 (Fig. 1c, d). Additionally, we observed low expression among other cells that are part of the endosteal niche (Fig. 1e). Minimal *Itga8* expression was observed in haematopoietic cells (Fig. 1f). Similarly, minimal *Itga8* expression was observed in endothelial cells and MSCs that constitute the perivascular niche (Fig. 1g).

To determine the localisation of Itga8^+^Alcam^+^Sca-1^−^ BLCs, we performed immunofluorescence staining of mouse BM. A small subset of Alcam⁺Sca-1^−^ BLCs residing at the endosteal surface of the cortical bone and the epiphysis expressed Itga8 (Fig. 1h). Itga8⁺ cells were not found in the central BM (Fig. 1h). Consistent with previous reports^27^, Sca-1 was highly expressed in the arterial endothelial cells in BM (Fig. 1h). These findings confirm that Itga8⁺ cells are a subset of the Alcam⁺Sca-1^−^ BLCs that are characteristically located at the endosteal surface of the BM.

### Itga8^+^Alcam^+^Sca-1^−^ BLCs exhibit distinct MSC characteristics

**Fig. 2:**
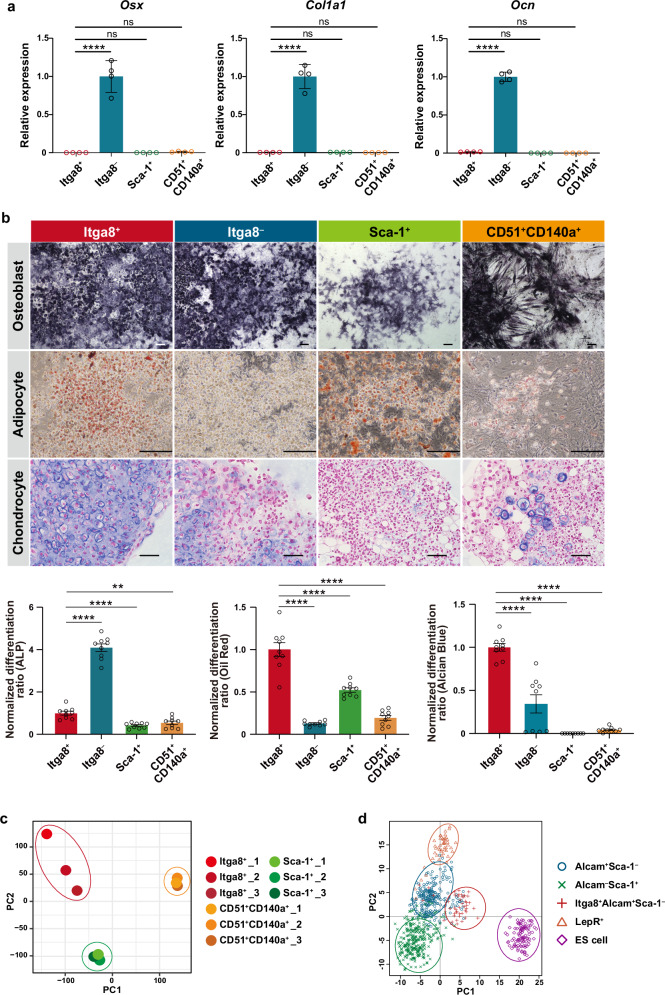
The depletion of Itga8^+^ MSCs leads to a reduction in functional HSCs. **a** Expression of osteoblast markers (*Osx*, *Col1a1* and *Ocn*) in Itga8^+^Alcam^+^Sca-1^−^ (Itga8^+^) BLCs, Itga8^−^Alcam^+^Sca-1^−^ (Itga8^−^) BLCs, Alcam^–^Sca-1^+^ MSCs and CD51^+^CD140a^+^ MSCs (*n* = 4/group). **b** Differentiation potential of Itga8^+^ BLCs, Itga8^−^ BLCs, Sca-1^+^ MSCs and CD51^+^CD140a^+^ MSCs after osteogenic, adipogenic and chondrogenic induction. Osteoblastic differentiation was evaluated by ALP staining, adipocyte differentiation by Oil Red O staining, and chondrocyte differentiation by Alcian blue staining. Scale bars: 100 μm (osteoblasts and adipocytes), 300 μm (chondrocytes). Quantification of each staining images. **c** Principal component analysis (PCA) of RNA-seq data of Itga8^+^ MSCs, Sca-1^+^ MSCs and CD51^+^CD140a^+^ MSCs (*n* = 3/group). **d** PCA of single-cell qPCR data of Alcam^+^Sca-1^−^ BLCs (blue, circle), Sca-1^+^ MSCs (green, cross), Leptin receptor (LepR)^+^ MSCs (brown, triangle), Itga8^+^Alcam^+^Sca-1^−^ BLCs (red, plus sign) and ES cells (purple, diamond). Itga8^+^Alcam^+^Sca-1^−^ BLCs form a distinct cluster, separate from other BLCs and MSCs. Data are presented as mean ± SD. Statistical differences were determined by ordinary one-way ANOVA followed by Tukey’s multiple-comparison test for (**a**). Statistical significance was determined by a two-sided unpaired *t*-test for (**b**). **p < 0.01, ****p < 0.0001 (**a**) (*Osx*) Itga8^+^ vs Itga8^−^: exact *p* = 6.5 × 10^−^^8^. (*Col1a1*) Itga8^+^ vs Itga8^−^: exact *p* = 3.0 × 10^−^^9^. (*Ocn*) Itga8^+^ vs Itga8^−^: exact *p* = 1.4 × 10^−^^13^. **b** (osteoblasts) Itga8^+^ vs Itga8^−^: exact *p* = 4.62 × 10^−^^11^. Itga8^+^ vs Sca-1^+^: exact *p* = 2.50 × 10^−^^5^. Itga8^+^ vs CD51^+^CD140a^+^: exact *p* = 0.0017. (adipocytes) Itga8^+^ vs Itga8^−^: exact *p* = 1.28 × 10^−^^8^. Itga8^+^ vs Sca-1^+^: exact *p* = 4.57 × 10^−^^5^. Itga8^+^ vs CD51^+^CD140a^+^: exact *p* = 7.96 × 10^−^^8^. (chondrocytes) Itga8^+^ vs Itga8^−^: exact *p* = 3.11 × 10^−^^5^. Itga8^+^ vs Sca-1^+^: exact *p* = 1.23 × 10^−^^13^. Itga8^+^ vs CD51^+^CD140a^+^: exact *p* = 3.44 × 10^−^^13^.

We characterised Itga8⁺Alcam⁺Sca-1^−^ BLCs to determine their properties. Expression analysis of osteoblast markers revealed that these cells, unlike the Itga8^−^ fraction, lacked an osteoblastic phenotype (Fig. 2a). These data indicate that Itga8⁺Alcam⁺Sca-1^−^ BLCs may represent a distinct type of undifferentiated mesenchymal cell compared to osteoblasts or their precursors.

Next, we analysed the differentiation potential of the Itga8⁺ and Itga8^−^ fractions of Alcam⁺Sca-1^−^ BLCs, as well as Alcam^−^Sca-1⁺ MSCs and CD51⁺CD140a⁺ BM-derived MSCs^20,28,29^. Consistent with our previous report^27^, Itga8^−^Alcam⁺Sca-1^−^ BLCs had a strong tendency to differentiate into osteoblasts. In contrast, the Itga8⁺ fraction differentiated into osteogenic, adipogenic and chondrogenic lineages with greater efficiency than Sca-1⁺ MSCs or CD51⁺CD140a⁺ MSCs (Fig. 2b). These data indicate that Itga8⁺ cells represent an MSC subset within the Alcam⁺Sca-1^−^ BLC population.

We then performed RNA sequencing to compare the gene expression profiles of Itga8⁺Alcam⁺Sca-1^−^ MSCs (hereafter referred to as Itga8⁺ MSCs) with those of other MSC populations. Principal component analysis (PCA) revealed that Itga8⁺ MSCs, Sca-1⁺ MSCs and CD51⁺CD140a⁺ MSCs formed distinct clusters (Fig. 2c). K-means clustering further categorised the gene expression profiles of these MSCs into six clusters (Extended Data Fig. 2a). Notably, Itga8⁺ MSCs highly expressed genes in cluster 1, which were enriched for pathways related to connective tissue development, cell adhesion and tissue development (Extended Data Fig. 2b). Additionally, Itga8⁺ MSCs showed high expression of genes in cluster 6, which was enriched for pathways such as multicellular organism development and tissue development (Extended Data Fig. 2g). In contrast, Sca-1⁺ MSCs had high expression of genes in clusters 2, 5 and 6, while CD51⁺CD140a⁺ MSCs had high expression of genes in clusters 3–5 (Extended Data Fig. 2c–g). These data suggest that Itga8⁺ MSCs constitute a unique subset of MSCs within the Alcam⁺Sca-1^−^ BLC population, that are actively involved in the formation, maintenance, and structural organisation of connective tissues. Furthermore, PCA analysis of the single-cell qPCR data revealed that Itga8⁺ MSCs are distinct from other MSC and BLC populations (Fig. 2d). To further characterise Itga8^+^ MSCs, we compared their transcriptional profile with that of N-cadherin^+^ MSCs using public gene expression data (Extended Data Fig. 3). N-cadherin^+^ MSCs have been described as an endosteal MSC population that contributes to HSC maintenance by anchoring HSCs within the niche. This analysis showed that Itga8^+^ MSCs were distinct from N-cadherin^+^ MSCs and exhibited different expression patterns of niche-related genes. These findings suggest that Itga8⁺ MSCs constitute an MSC population within the BM.

### Depletion of Itga8^+^ MSCs reduces HSC number and LTR capacity

**Fig. 3:**
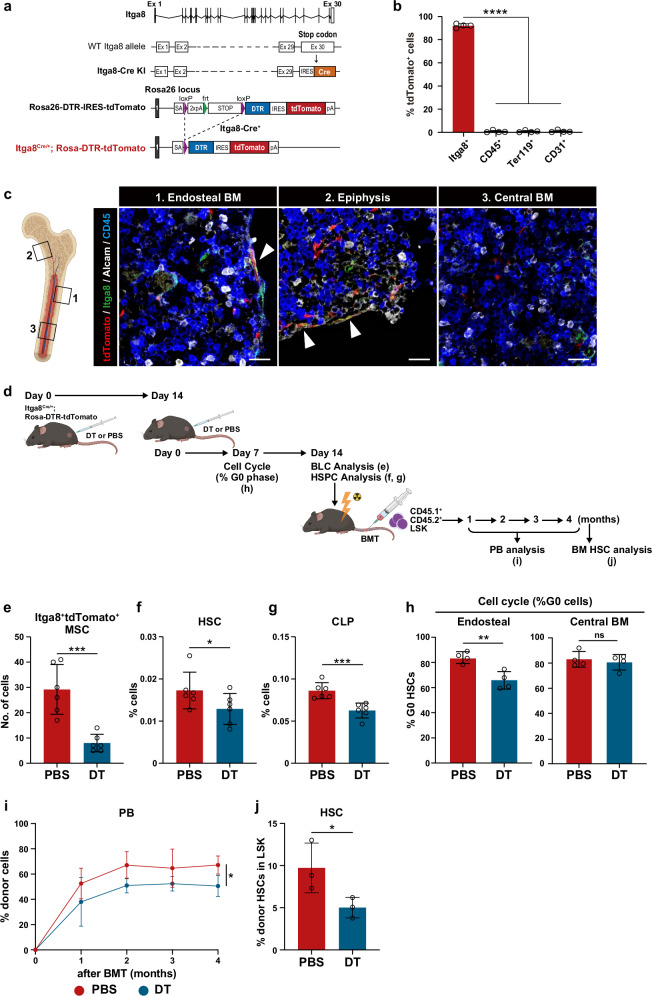
The depletion of Itga8^+^ MSCs leads to a reduction in functional HSCs. **a** Generation of Itga8^Cre/+^; Rosa-DTR-tdTomato mice. By crossing Itga8-Cre knock-in mice with Rosa-DTR-tdTomato mice, expression of DTR and tdTomato is induced characteristically in Itga8^+^ cells. **b** The percentage of tdTomato^+^ cells in Itga8^+^ MSCs, CD45^+^ cells, Ter119^+^ cells, or CD31^+^ cells (*n* = 4/group). **c** Immunohistochemical staining of tdTomato (red), Itga8 (green), Alcam (white) and CD45 (blue) in BM sections of Itga8^Cre/+^; Rosa-DTR-tdTomato mice. Itga8^+^tdTomato^+^Alcam^+^ MSCs (arrowheads). Scale bars: 20 μm. **d** Schematic representation of flow cytometric analysis and BMT following depletion of Itga8^+^ cells by DT administration in Itga8^Cre/+^; Rosa-DTR-tdTomato mice. Mice received DT or PBS on day 0 and day 14. Cell cycle analysis was performed on day 7, and flow cytometry analysis of BLCs and BM was conducted on day 14 after the final DT treatment. LSK cells were isolated and transplanted into recipient mice with competitor cells. **e–g** Representative flow cytometry analysis of BLCs and BM in Itga8^Cre/+^; Rosa-DTR-tdTomato mice after DT or control (PBS) treatment (*n* = 6/group). **e** The number of Itga8^+^tdTomato^+^ MSCs. **f** The proportion of HSCs (LSKCD48^−^CD150^+^). **g** The proportion of CLPs (CD127^+^Lin^−^Sca-1^low^c-kit^low^). **h** The percentage of HSCs in the G0 phase around the endosteum (left) and central BM (right). **i** The percentage of donor-derived cells after 1–4 months of BMT (*n* = 3/group). **j** The frequency of donor-derived HSCs in recipient mice 4 months after BMT (*n* = 3/group). Data are presented as mean ± SD. Statistical differences were determined by ordinary one-way ANOVA followed by Tukey’s multiple-comparison test for (**b**). Statistical significance was determined by a one-sided unpaired *t*-test for (**e–j**). *p < 0.05, **p < 0.01, ***p < 0.001, ****p < 0.0001 (**b**) Itga8^+^ vs CD45^+^: exact *p* = 2.6 × 10^−^^14^. Itga8^+^ vs Ter119^+^: exact *p* = 2.6 × 10^−^^14^. Itga8^+^ vs CD31^+^: exact *p* = 2.6 × 10^−^^14^. (**e**) exact *p* = 0.0003. **f** exact *p* = 0.0444. **g** exact *p* = 0.0006 (**h**) exact *p* = 0.0049. **i** exact *p* = 0.0296. **j** exact *p* = 0.0315. Created in BioRender. Arai, F. (2026) https://BioRender.com/weipum5, https://BioRender.com/jykdosl, https://BioRender.com/xghxfih, and https://BioRender.com/ykyzx28.

To investigate the role of Itga8^+^ MSCs in maintaining HSCs in the BM, we prepared Itga8-cre recombinase (Cre) knock-in (KI) mice and generated Itga8^Cre/+^; Rosa-diphtheria toxin receptor (DTR)-tdTomato mice by crossing Itga8-Cre KI mice with Rosa26-loxP-stop-loxP-DTR-tdTomato mice^30^. This mouse model enables tdTomato expression in Itga8⁺ MSCs and allows for their conditional ablation by injecting diphtheria toxin (DT) (Fig. 3a). Flow cytometry analysis confirmed tdTomato expression in Itga8^+^ MSCs, while no tdTomato expression was identified in haematopoietic or endothelial cells (Fig. 3b and Extended Data Fig. 4a). We also observed that Itga8^+^ MSCs were localised on the endosteal surface expressing tdTomato (Fig. 3c, Extended Data Fig. 4b). Itga8 expression at both the protein and mRNA levels was substantially lower in Sca-1⁺ MSCs and CD51⁺CD140a⁺ MSCs than in Itga8⁺ MSCs (Extended Data Fig. 4c, d). Interestingly, in Itga8^Cre/+^; Rosa-DTR-tdTomato mice, tdTomato expression was observed in other MSC subsets, but at lower levels compared to Itga8⁺ MSCs (Extended Data Fig. 4e). To further examine the lineage relationship between Itga8^+^ MSCs and other MSC populations, we generated LepR^Cre/+^; Rosa-DTR-tdTomato mice. LepR is expressed in a large proportion of perivascular MSCs, and this mouse model enabled us to determine whether LepR^+^ MSCs give rise to Itga8^+^ MSCs. As shown in Extended Data Fig. 5a, b, most CD51⁺CD140a⁺ MSCs were tdTomato^+^, whereas only a few Itga8⁺ MSCs and Sca-1⁺ MSCs were tdTomato^+^ in LepR^Cre/+^; Rosa-DTR-tdTomato mice. Together, these findings suggest that Itga8⁺ MSCs may be positioned upstream of other MSC subsets. Moreover, we investigated the effects of the depletion of LepR^+^ MSC on niches and haematopoiesis by injection of DT into LepR^Cre/+^; Rosa-DTR-tdTomato mice on day 0 and day 14 to deplete LepR^+^ MSCs. The number of tdTomato^+^ cells in the central BM decreased with DT administration (Extended Data Fig. 5c), but not in the endosteal region (Extended Data Fig. 5d). Additionally, no changes were observed in the proportion of Itga8^+^ cells among tdTomato^+^ cells, the number of Alcam^+^ cells, the number of Alcam^+^Itga8^+^ cells, or the number of Sca-1^+^ cells in the endosteal region (Extended Data Fig. 5e–h). Furthermore, we analysed haematopoietic cells, including HSCs and progenitor cells. We found no change in total mononuclear cell (MNC) counts after DT administration (Extended Data Fig. 5i–n). However, we observed an increase in HSCs, CMPs, and GMPs after depleting LepR^+^ MSCs.

We then investigated the function of Itga8^+^ MSCs on haematopoiesis within the BM by injection of DT into Itga8^Cre/+^; Rosa-DTR-tdTomato mice on day 0 and day 14 to deplete Itga8^+^ MSCs (Fig. 3d and Extended Data Fig. 6a). On day 14 after the final injection, 71.6 ± 9.0% of Itga8⁺tdTomato⁺ MSCs were depleted by DT administration (Fig. 3e). Depletion of Itga8⁺ MSCs resulted in a reduction of HSCs and common lymphoid progenitors (CLPs) (Fig. 3f, g). In contrast, the total number of MNCs and the proportions of common myeloid progenitors (CMPs), granulocyte-macrophage progenitors (GMPs) cells and megakaryocyte-erythrocyte progenitors (MEPs) remained unchanged (Extended Data Fig. 6b–e). Additionally, there were no differences in peripheral blood (PB) complete blood counts (CBCs) or in the proportions of myeloid, B and T cells (Extended Data Fig. 6s). Furthermore, analysis of the cell cycle status of HSCs in central BM, which contains the vascular niche, and periosteal BM, which contains the periosteal niche, revealed that the proportion of quiescent (G0)-phase HSCs remained unchanged in central BM but decreased in periosteal BM in DT-treated mice (Fig. 3h). These results suggest that Itga8^+^ MSCs play an important role in maintaining the HSC pool, particularly the periendosteal HSCs. The depletion of Itga8^+^ MSCs did not promote HSC apoptosis (Extended Data Fig. 6l). Next, to determine whether Itga8^+^ MSCs also play a crucial role in maintaining the long-term repopulating capacity of HSCs, lineage (Lin)^−^Sca-1^+^c-Kit^+^ (LSK) cells obtained from control or DT-treated mice were transplanted into lethally irradiated recipient mice. PB from recipient mice was analysed monthly for up to 4 months after the BM transplantation (BMT) (Extended Data Fig. 7a). PB analysis revealed that the engraftment of LSK cells from DT-treated mice was lower than control LSK cells (Fig. 3i). Differentiation of donor cells into myeloid, B and T cell lineages was comparable between control and DT-treated groups (Extended Data Fig. 7b–d). Additionally, the LTR of donor cells within BM LSK cells from DT-treated mice was lower than that of control LSK cells (Fig. 3j). These results suggest that Itga8⁺ MSCs are critical for maintaining HSC function and long-term repopulating capacity. Itga8^Cre/+^; Rosa-DTR-tdTomato mice treated with PBS or DT were administered 5-fluorouracil (5-FU), and haematopoietic cells were observed ten days later (Extended Data Fig. 8a–j). Compared to the controls, the BM of the DT-treated Itga8^Cre/+^; Rosa-DTR-tdTomato mice showed reduced total MNC, GMPs, MEPs, and B cell counts, which confirms the delayed haematopoietic recovery following 5-FU administration. These results suggest that, under 5-FU-induced myelosuppressive stress, Itga8^+^ MSCs provide a protective microenvironment for endosteal HSCs.

### Itga8^+^ MSCs maintain HSC long-term repopulating capacity

**Fig. 4:**
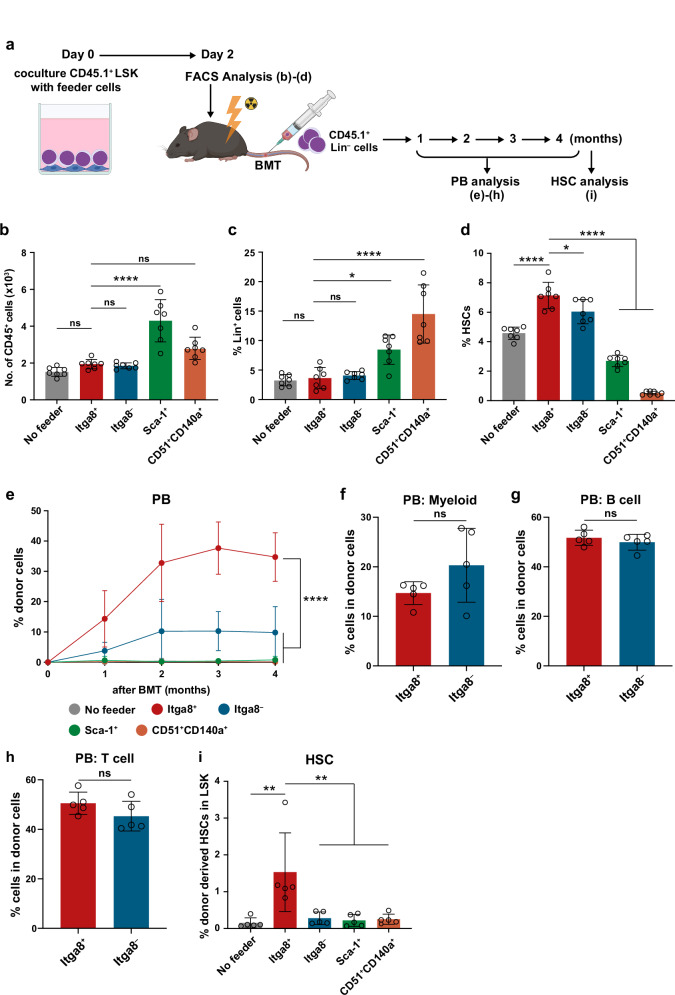
Itga8^+^ MSCs preserve the LTR capacity of HSCs. **a** Schematic representation of the coculture of LSK cells with Itga8^+^ MSCs, Itga8^−^Alcam^+^Sca-1^−^ BLCs, Sca-1^+^ MSCs and CD51^+^CD140a^+^ MSCs. LSK cells are cocultured with a feeder layer of each MSC or BLC for 2 days in MyeloCult without cytokines. After coculture, the number of CD45^+^ cells and the frequency of Lin^+^ cells and HSCs were analysed. Similarly, 200 Lin^−^CD45.1^+^ cells are transplanted into recipient mice (CD45.2^+^) with 2 × 10^5^ CD45.2^+^ competitor cells. **b** The number of CD45^+^ cells after coculture (*n* = 7/group). **c** The proportion of Lin^+^ cells in CD45^+^ cells (*n* = 7/group). **d** The proportion of LSKCD48^−^CD150^+^ HSCs in CD45^+^ cells (*n* = 7/group). **e** The percentage of donor-derived cells in PB after 1–4 months of BMT (*n* = 5/group). **f** The percentage of donor-derived cells in myeloid cells after 4 months of bone marrow transplantation (BMT) (*n* = 5/group). **g** The percentage of donor-derived cells in B cells after 4 months of BMT (*n* = 5/group). **h** The percentage of donor-derived cells in T cells after 4 months of BMT (*n* = 5/group). **i** The percentage of donor-derived HSCs in LSK cells 4 months after BMT (*n* = 5/group). Data are presented as mean ± SD. Statistical differences were determined by ordinary one-way ANOVA followed by Tukey’s multiple-comparison test for (**b–e** and **i**). Statistical significance was determined by a two-sided unpaired *t*-test for (**f–h**). *p < 0.05, **p < 0.01, ***p < 0.0001 (**b**) Itga8^+^ vs Sca-1^+^: exact *p* = 1.4 × 10^−8^. **c** Itga8^+^ vs Sca-1^+^: exact *p* = 0.016. Itga8^+^ vs CD51^+^CD140a^+^: exact *p* = 1.5 × 10^−^^7^. **d** Itga8^+^ vs No feeder: exact *p* = 6.9 × 10^−8^. Itga8^+^ vs Itga8^−^: exact *p* = 0.015. Itga8^+^ vs Sca-1^+^: exact *p* = 2.1 × 10^−^^13^. Itga8^+^ vs CD51^+^CD140a^+^: exact *p* = 5.0 × 10^−^^14^. **e** Itga8^+^ vs No feeder: exact *p* = 1.6 × 10^−8^. Itga8^+^ vs Itga8^−^: exact *p* = 3.0 × 10^−^^6^. Itga8^+^ vs Sca-1^+^: exact *p* = 2.2 × 10^−8^. Itga8^+^ vs CD51^+^CD140a^+^: exact *p* = 1.6 × 10^−8^. **i** Itga8^+^ vs No feeder: exact *p* = 0.0024. Itga8^+^ vs Itga8^−^: exact *p* = 0.0059. Itga8^+^ vs Sca-1^+^: exact *p* = 0.0039. Itga8^+^ vs CD51^+^CD140a^+^: exact *p* = 0.0048. Representative data from 2 experiments are shown (**f–h**). Created in BioRender. Arai, F. (2026) https://BioRender.com/ykyzx28, https://BioRender.com/ol6x0bs, https://BioRender.com/sepx0do, and https://BioRender.com/xghxfih.

Next, we investigated the capacity of Itga8^+^ MSCs to support HSC activity in culture mediums. In our previous study, we reported that Alcam^+^Sca-1^−^ BLCs in the endosteal niche maintained the BM repopulation capacity of HSCs in coculture, implying that these cells play a central role in maintaining and supporting HSCs within a specialised microenvironment^26^. To investigate the function of Itga8^+^ MSCs in supporting HSCs, we cocultured LSK cells with Itga8^+^ MSCs or other mesenchymal cells without cytokines (Fig. 4a). We observed that the number of CD45^+^ cells increased most significantly when cocultured with Sca-1⁺ MSCs (Fig. 4b). Additionally, coculture with CD51⁺CD140a⁺ MSCs resulted in a significant increase in differentiated Lin⁺ blood cells, and Sca-1⁺ MSCs also supported the expansion of Lin⁺ cells (Fig. 4c). In contrast, Itga8⁺ MSCs and Itga8^−^Alcam⁺BLCs suppressed the increase of Lin⁺ cells. These results suggest that Sca-1⁺ MSCs and CD51⁺CD140a⁺ MSCs promote HSC differentiation in culture. Conversely, the number of LSKCD48^−^CD150⁺ HSCs after coculture was maintained in both Itga8⁺ and Itga8^−^Alcam⁺BLC fractions, with Itga8⁺ MSCs particularly supporting the highest number of HSCs (Fig. 4d and Extended Data Fig. 9). These findings indicate that Itga8⁺ MSCs can preserve HSCs in an undifferentiated state. Next, to analyse the LTR capacity of HSCs cocultured with each MSC or BLC fraction, we engrafted 200 Lin^−^ cells into lethally irradiated recipient mice following the coculture (Fig. 4a). We observed that HSC cocultured with Itga8^+^ MSCs and Itga8^−^Alcam⁺BLCs successfully engrafted in recipient mice, whereas HSCs cocultured with Sca-1⁺ MSCs and CD51⁺CD140a⁺ MSCs showed minimal engraftment (Fig. 4e). Differentiation into myeloid, B and T cells was comparable between cells cultured with Itga8^+^ MSCs and Itga8⁻Alcam⁺BLCs (Fig. 4f–h). Furthermore, among the Alcam⁺ BLC fractions, HSCs cocultured with Itga8⁺ MSCs exhibited the highest LTR capacity (Fig. 4e). Additionally, HSCs cocultured with Itga8⁺ MSCs demonstrated higher engraftment in the BM HSC fraction compared to those cocultured with other MSC populations (Fig. 4i). These data suggest that Itga8⁺ MSCs possess HSC niche cell properties and maintain the long-term repopulating capacity of HSCs in culture.

### Itga8⁺ MSCs maintain HSCs by cell-cell and ECM interactions

**Fig. 5:**
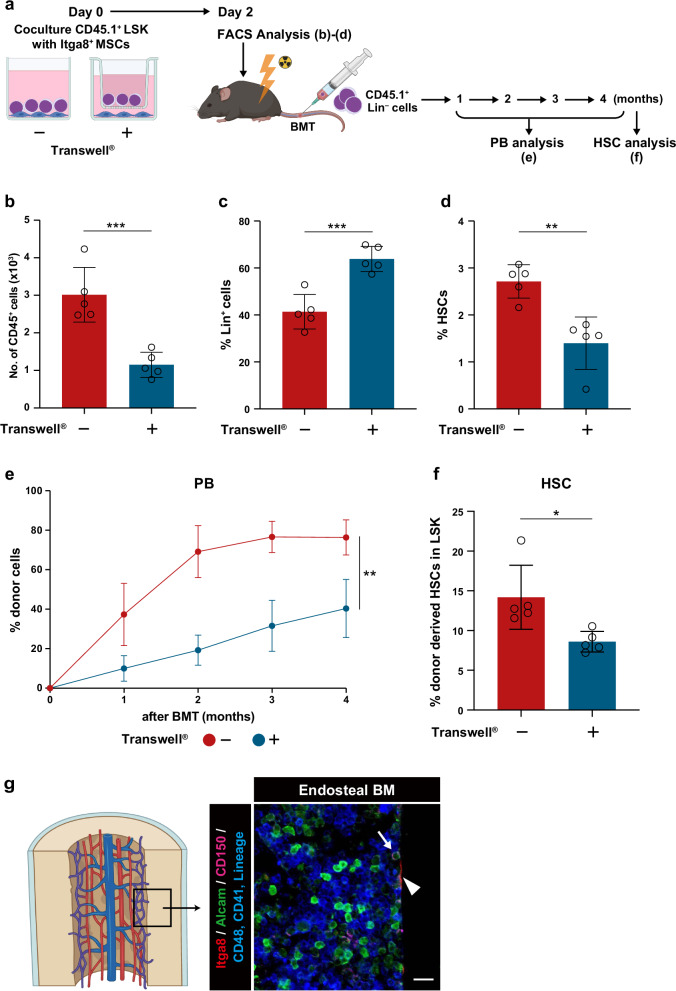
Itga8^+^ MSCs maintain the self-renewal capacity of HSCs through a cell adhesion-related mechanism. **a** Schematic representation of the coculture of LSK cells with Itga8^+^ MSCs with or without a Transwell. After 2 days of coculture, the number of CD45^+^ cells and the frequency of Lin+ cells and HSCs were analysed. Simultaneously, 200 Lin^−^CD45.1^+^ cells were transplanted into CD45.2^+^ irradiated mice along with 2 × 10^5^ CD45.2^+^ competitor cells. **b** The number of CD45^+^ cells after coculture. **c** The proportion of Lin^+^ cells in CD45^+^ haematopoietic cells. **d** The proportion of LSKCD48^−^CD150^+^ HSCs in CD45^+^ cells. **e** The percentage of donor-derived cells in PB after 1–4 months of BMT. **f** The percentage of donor-derived HSCs in LSK cells 4 months after BMT. **g** Immunohistochemical staining of Itga8 (red), Alcam (green), CD150 (magenta), and CD48, CD41 and Lin (blue) in the endosteal area of BM. Arrowheads indicate Itga8^+^Alcam^+^ cells and arrows indicate Lin^−^CD48^−^CD41^−^CD150^+^ cells. Scale bars: 20 μm. Data are presented as mean ± SD (*n* = 5/group). Statistical significance was determined by a two-sided unpaired *t*-test. *p < 0.05, **p < 0.01, ***p < 0.001. **b** exact *p* = 0.0008. **c** exact *p* = 0.0006. **d** exact *p* = 0.0022. **e** exact *p* = 0.0016. **f** exact *p* = 0.0183. Representative data from 2 experiments are shown. Created in BioRender. Arai, F. (2026) https://BioRender.com/ykyzx28, https://BioRender.com/ol6x0bs, https://BioRender.com/sepx0do, https://BioRender.com/xghxfih, and https://BioRender.com/ecnmnm0.

The above findings indicated that Itga8^+^ MSCs are involved in the maintenance of HSCs in the BM. However, the gene expression profile revealed that Itga8^+^ MSCs have low expression of HSC niche factors such as *Cxcl12*, *Scf* and *Angpt1*, which have been reported to play important roles in the maintenance of HSCs (Extended Data Fig. 10a)^6–8^. Therefore, Itga8^+^ MSCs seem to maintain HSC self-renewal through mechanisms distinct from known soluble niche factors. For this reason, we sought to identify HSC niche factors derived from Itga8^+^ MSCs, focusing on cell adhesion-mediated mechanisms as a potential pathway for HSC maintenance. To confirm the necessity of cell adhesion molecule interactions between HSCs and Itga8^+^ MSCs in HSC maintenance, we cocultured LSK cells with Itga8⁺ MSCs on Transwell^®^ inserts (Fig. 5a). We found that coculture with Transwell^®^ suppressed the expansion of CD45^+^ cells, increased Lin^+^ cells, and decreased HSCs (Fig. 5b–d and Extended Data Fig. 10b), suggesting that Itga8⁺ MSCs maintain HSCs through cell adhesion. We next transplanted Lin^−^ cells from coculture with or without Transwell^®^ into the lethally irradiated mice and analysed their LTR activity. According to PB analysis after BMT, the engraftment rate of donor cells from coculture with Transwell^®^ was lower than that of cells cocultured without Transwell^®^ (Fig. 5e). Additionally, the proportion of donor-derived HSCs was reduced by using Transwell^®^ (Fig. 5f). Differentiation into myeloid, B and T cells was comparable between cells cultured with or without Transwell^®^ (Extended Data Fig. 10c–e). These data suggest that cell-to-cell and/or cell-to-ECM interactions are crucial for HSC maintenance by Itga8⁺ MSCs. Indeed, we assessed the localisation of Itga8^+^ MSCs and HSCs in the BM by immunofluorescence staining and confirmed that HSCs were localised adjacent to Itga8^+^ MSCs around the endosteal region (Fig. 5g).

### Mfap4 as a candidate factor in HSC maintenance

**Fig. 6:**
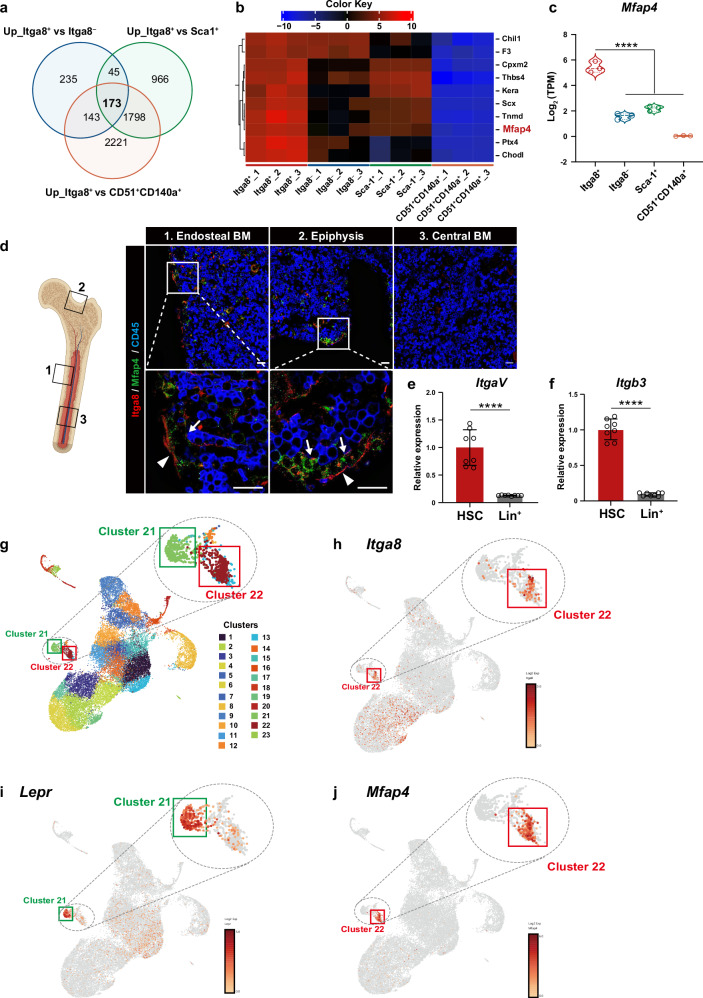
Mfap4, highly expressed in Itga8^+^ MSCs, increases the self-renewal capacity of HSCs. **a** Venn diagrams showing the number of DEGs in Itga8^+^ MSCs compared to Itga8^−^Alcam^+^ BLCs (blue), Sca-1^+^ MSCs (green) and CD51^+^CD140a^+^ MSCs (red). Taking the intersection of these sets, 173 genes were identified as characteristically highly expressed in Itga8^+^ MSCs. The numbers indicate the number of genes in each category. **b** Heatmap with hierarchical clustering based on RNA-seq data from Itga8^+^ MSCs, Itga8^−^Alcam^+^ BLCs, Sca-1^+^ MSCs and CD51^+^CD140a^+^ MSCs, displaying 10 genes highly expressed in Itga8^+^ MSCs. **c** Comparison of TPM-based expression of Mfap4 in Itga8^+^ MSCs, Itga8^−^Alcam^+^ BLCs, Sca-1^+^ MSCs and CD51^+^CD140a^+^ MSCs (*n* = 3/group). **d** Expression of *Itgav* in HSC and Lin^+^ cells (*n* = 8/group). **e** Expression of *Itgb3* in HSC and Lin^+^ cells (*n* = 8/group). **f** Immunohistochemical staining of Itga8 (red), Mfap4 (green) and CD45 (blue). (1): Endosteal region, (2): Epiphysis, (3): Central BM. The lower panels are magnified views of the area outlined by the box in the upper panel. Itga8^+^MSCs (arrowheads) and Mfap4^+^ (arrows). Scale bars: 20 μm. **g** Uniform manifold approximation and projection (UMAP) visualization of Alcam^+^ BLCs showing unsupervised clusters (1–23). **h**–**j** UMAP feature plot showing the expression of **h***Itga8*, **i***Lepr*, and **j***Mfap*4 in Alcam⁺ BLCs, with each dot represents a single cell, colours indicates log_2_-normalised expression levels. Data are presented as mean ± SD. Statistical differences were determined by ordinary one-way ANOVA followed by Tukey’s multiple-comparison test for (**c**). Statistical significance was determined by a two-sided unpaired *t*-test for (**e** and **f**). ****p < 0.0001 (**c**) Itga8^+^ vs Itga8^−^: exact *p* = 4.7 × 10^−^^7^. Itga8^+^ vs Sca-1^+^: exact *p* = 2.0 × 10^−^^6^. Itga8^+^ vs CD51^+^CD140a^+^: exact *p* = 2.8 × 10^−^^8^. **e** exact *p* = 2.7 × 10^−^^6^. **f** exact *p* = 6.5 × 10^−^^11^. Created in BioRender. Arai, F. (2026) https://BioRender.com/weipum5.

To identify the factors expressed in Itga8⁺ MSCs responsible for the maintenance of HSCs, we compared differentially expressed genes (DEGs) using RNA sequencing analysis of Itga8^+^ MSCs, Itga8^−^Alcam^+^ BLCs, Sca-1^+^ MSCs and CD51^+^CD140a^+^ MSCs. Referring to the data presented earlier, we focused on the adhesion molecules and ECM components characteristically expressed in Itga8⁺ MSCs. First, a comparison of DEGs between Itga8^+^ MSCs and Itga8^−^Alcam^+^ BLCs revealed that 596 genes were upregulated in Itga8^+^ MSCs (Extended Data Fig. 11a). Similarly, 2982 genes were upregulated in Itga8^+^ MSCs compared to Sca-1^+^ MSCs (Extended Data Fig. 11b). Additionally, 4335 genes were upregulated in Itga8^+^ MSCs compared to CD51^+^CD140a^+^ MSCs (Extended Data Fig. 11c). By taking the intersection of these three gene sets, we identified 173 genes that were characteristically highly expressed in Itga8^+^ MSCs (Fig. 6a). In the top 10 genes with the highest expression levels in Itga8^+^ MSCs we found four candidate genes related to cell adhesion: microfibrillar-associated protein 4 (*Mfap4*), tenomodulin (*Tnmd*), chondrolectin (*Chodl*) and keratocan (*Kera*) (Fig. 6b and Extended Data Fig. 11d–f). Among these, Mfap4 attracted our attention due to its high evolutionary conservation and potential role in haematopoiesis.

Mfap4 is an ECM protein belonging to the fibrinogen-related domain family^31^. In zebrafish, the loss of *Mfap4* has been shown to reduce the expression of key transcription factors essential for definitive haematopoiesis, such as *Gata2* and *Runx1*^32–34^, and has been associated with a decrease in macrophages and an increase in neutrophils^32^. These findings raised the possibility that Mfap4 might contribute to HSC-supportive functions of Itga8^+^ MSCs in mammals. We confirmed that Mfap4 expression is higher in Itga8^+^ MSCs compared to other MSCs (Fig. 6c and Extended Data Fig. 11g). Additionally, integrin αV (*ItgaV*) and integrin β3 (*Itgb3*), the binding partners of Mfap4, were expressed on HSCs (Fig. 6d, e)^31^. Consistent with this, immunostaining revealed that Mfap4 was predominantly expressed in the endosteal region in the BM and co-localised with Itga8^+^ MSCs (Fig. 6f). Mfap4 expression was not observed in the central region of the BM (Fig. 6f).

To further define the transcriptomic identity of the Itga8^+^ subpopulation and examine its relationship to candidate niche factors and other stromal cell populations, including LepR^+^ BLCs, we performed single-cell RNA sequencing (scRNA-seq) of Alcam^+^ BLCs (Fig. 6g–j and Extended Data Fig. 12). Alcam^+^ BLCs were divided into 23 clusters, and Itga8 expression was enriched in Cluster 22. *Mfap4* and other candidate niche factors, including *Kera*, *Tnmd*, *Chodl* and *Ptx4*, were also enriched in the same cluster, whereas known niche factors such as *Cxcl12* and *Kitl* were enriched in Cluster 21, which expressed *Lepr*. These findings indicate that the Itga8^+^ MSC population is transcriptionally distinct from LepR^+^ BLCs and is characterised by expression of multiple ECM-related candidate niche factors.

### Effects of Mfap4 on ex vivo HSC maintenance

**Fig. 7:**
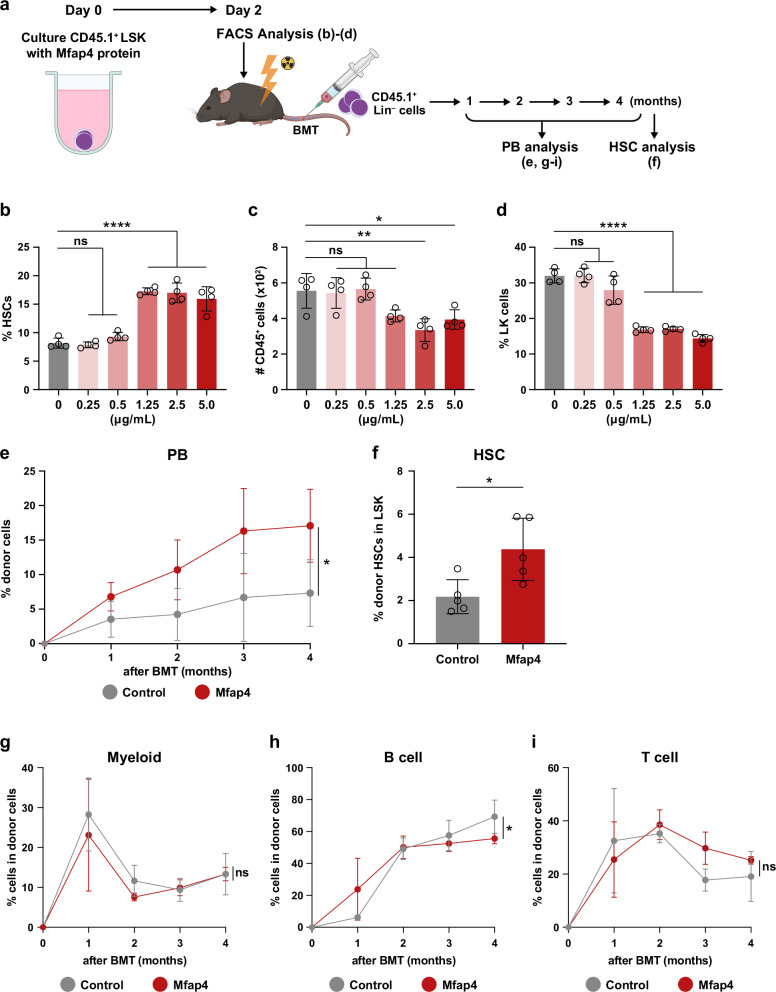
Mfap4 influences ex vivo HSC maintenance. **a** Schematic representation of the evaluation of the effect of Mfap4. LSK cells (CD45.1^+^) were cultured for 2 days with or without Mfap4. After 2 days of coculture, the number of CD45^+^ cells and the frequency of Lin^–^Kit^+^ (LK) cells and HSCs were analysed. Similarly, Lin^−^CD45.1^+^ cells were transplanted into CD45.2^+^ irradiated recipient mice along with competitor cells. **b** The proportion of LSKCD48^–^CD150^+^ HSCs. **c** The number of CD45^+^ cells. **d** The proportion of LK cells. **e** The percentage of donor-derived cells after BMT following treatment with Mfap4 (2.5 μg/ml). **f** The percentage of donor-derived HSCs in LSK cells 4 months after BMT. **g** Percentage of donor-derived myeloid cells after 1–4 months of BMT. **h** The percentage of donor-derived B cells after 1–4 months of BMT. **i** The percentage of donor-derived T cells after 1–4 months of BMT. Data are presented as mean ± SD (*n* = 4/group). Statistical differences are determined by ordinary one-way ANOVA followed by Tukey’s multiple-comparison test for (**b–d**). Statistical significance was determined by a two-sided unpaired *t*-test for (**e–i**). *p < 0.05, ****p < 0.0001 (**b**) 0 μg/ml vs 1.25 μg/ml: exact *p* = 8.5 × 10^−^^8^, 0 μg/ml vs 2.5 μg/ml: exact *p* = 1.3 × 10^−^^7^, 0 μg/ml vs 5.0 μg/ml: exact *p* = 9.3 × 10^−^^7^. **c** 0 μg/ml vs 2.5 μg/ml: exact *p* = 0.0032, 0 μg/ml vs 5.0 μg/ml: exact *p* = 0.04. **d** 0 μg/ml vs 1.25 μg/ml: exact *p* = 8.5 × 10^−^^8^, 0 μg/ml vs 2.5 μg/ml: exact *p* = 1.0 × 10^−^^7^, 0 μg/ml vs 5.0 μg/ml: exact *p* = 7.8 × 10^−^^9^. **e** exact *p* = 0.0347. **f** exact *p* = 0.0173. **h** exact *p* = 0.0454. Created in BioRender. Arai, F. (2026) https://BioRender.com/qlmjoux, https://BioRender.com/xghxfih, and https://BioRender.com/ykyzx28.

Next, we examined the effects of Mfap4 on ex vivo HSC maintenance. LSK cells were cultured in the absence of cytokines with various concentrations of Mfap4 protein for 2 days, and the expansion of haematopoietic cell populations and HSCs was then evaluated. Mfap4 supplementation suppressed the expansion of CD45^+^ cells in a dose-dependent manner, while maintaining the proportion of LSKCD48^−^CD150^+^ HSCs (Fig. 7a–c). In contrast, the frequency of Lin^−^c-Kit^+^ (LK) cells decreased (Fig. 7d). These findings suggest that Mfap4 may help preserve the immature haematopoietic stem/progenitor cell (HSPC) compartment rather than simply promoting overall haematopoietic expansion.

To further examine whether Mfap4 influences cell-cycle status or haematopoietic differentiation under these culture conditions, we analysed the cell cycle and differentiation status of LSK cells cultured in MyeloCult medium with or without Mfap4. LSK cells predominantly resided in the G0 phase, and this was unchanged between the two groups (Extended Data Fig. 13a, b). Likewise, Mfap4 protein had no significant effect on haematopoietic differentiation markers in cultured LSK cells (Extended Data Fig. 13c–f).

We next investigated whether Mfap4 treatment influences HSC function after ex vivo culture. LSK cells were cultured with Mfap4 in the absence of other cytokines for 2 days, and Lin^−^ cells were then collected and transplanted into lethally irradiated recipient mice. LSK cells treated with Mfap4 showed higher donor cell engraftment in PB compared with control cells (Fig. 7e). BM analysis also showed maintenance of donor-derived cells in the BM HSC fraction after Mfap4 treatment (Fig. 7f). Differentiation of donor cells into myeloid, B and T cell lineages was comparable between control and Mfap4-treated groups (Fig. 7g–i). Together, these data suggest that Mfap4 may contribute to the maintenance of HSC function during ex vivo culture.

## Discussion

In this study, we identified Itga8^+^ MSCs as a unique subpopulation within the Alcam^+^Sca-1^−^ BLCs that constitute the endosteal niche in the BM. Our findings demonstrate that Itga8^+^ MSCs have a critical role in maintaining HSC function and quiescence. Ablation of Itga8^+^ MSCs in Itga8^Cre/+^; Rosa-DTR-tdTomato mice reduced HSC numbers and LTR activity, indicating the importance of this MSC subset in supporting HSCs.

Previous studies have highlighted the complexity of the HSC niche, emphasising the roles of distinct microenvironments, such as the endosteal and vascular niches, in regulating HSC self-renewal and quiescence through specific cellular interactions and signalling pathways^3–5^. While perivascular MSCs, particularly CD51^+^CD140a^+^ MSCs, are known to secrete important haematopoietic niche factors like SCF and CXCL12^6,7,29^, our data suggest that Itga8^+^ MSCs support HSCs through alternative mechanisms^6,7,29^.

Notably, the functional consequences of MSC subset deletion differed markedly depending on the targeted population. Deletion of LepR⁺ MSCs resulted in an increase in the number of phenotypic HSCs, whereas deletion of Itga8⁺ MSCs led to a decrease. This divergence may reflect functional differences between these niche populations. Loss of LepR⁺ MSCs may impair quiescence-supporting signals mediated by Scf and Cxcl12, thereby moving HSCs out of quiescence and increasing the number of phenotypic HSCs. In contrast, depletion of Itga8⁺ MSCs in the endosteal niche may disrupt the ECM-dependent protective microenvironment for HSCs, thereby compromising maintenance of the HSC pool. Collectively, these findings suggest that functional heterogeneity between LepR⁺ MSCs and Itga8⁺ MSCs may underlie the distinct changes in HSC pool size observed after their respective ablation.

Interestingly, although flow cytometry and qPCR analyses showed that Sca-1^+^ MSCs and CD51^+^CD140a^+^ MSCs exhibit minimal current expression of Itga8, examination of Itga8^Cre/+^; Rosa-DTR-tdTomato mice revealed tdTomato expression in these MSC populations, albeit at lower levels than in Itga8^+^ MSCs. However, Itga8⁺ MSCs in LepR^Cre/+^; Rosa-DTR-tdTomato mice exhibited little or no tdTomato expression. These observations suggest a potential hierarchy within the MSC compartment of the HSC niche, in which rare Itga8^+^ MSCs may serve as a reservoir-like population that gives rise to other MSC subsets, such as Sca-1^+^ MSCs, CD51^+^CD140a^+^ MSCs, and LepR^+^ MSCs, and may therefore contribute to tissue maintenance or repair under specific conditions. Understanding this hierarchy is crucial, as it may explain how different MSC populations contribute to HSC maintenance through a variety of mechanisms, including the secretion of soluble cytokines, cell adhesion molecules, and ECMs. Further lineage tracing studies are needed to determine the differentiation pathways and hierarchical relationships among these MSC subsets.

Regarding the maintenance of HSC function by Itga8^+^ MSCs, our coculture experiments showed that direct cell-to-cell contact and cell-to-ECM adhesion are both crucial for Itga8^+^ MSCs to sustain the immature HSC phenotype. Notably, RNA-seq analysis revealed that Itga8^+^ MSCs did not express known niche factors such as *Cxcl12*, *Scf* and *Angpt1*, which were highly expressed in CD51^+^CD140a^+^ MSCs. Instead, we found that Itga8^+^ MSCs characteristically expressed ECM-related molecules, including *Mfap4*, *Tnmd*, *Chodl* and *Kera*. Because HSCs expressed *Itgav* and *Itgb3*, which are known to interact with *Mfap4*, we focused on *Mfap4* among those ECM factors expressed in Itga8^+^ MSCs.

The interaction between Mfap4 produced by the niche and Itgavb3 expressed on HSCs may activate the Focal adhesion kinase (FAK)/PI3K/AKT signalling pathway, resulting in the induction of the cell cycle regulator Cdkn1b and maintenance of HSC quiescence^35,36^. Previous studies have shown that HSC-specific deletion of Itgav results in reduced FAK/PI3K/AKT pathway activation and decreased Cdkn1b expression, impairing HSC quiescence^37^. Furthermore, our coculture experiments indicated that Itga8^+^ MSCs suppress haematopoietic cell proliferation, supporting the view that ECM-mediated adhesion signals in HSCs contribute to the maintenance of cell cycle quiescence. Further studies on Mfap4 and downstream integrin-associated signaling may help clarify the mechanistic basis by which Itga8^+^ MSCs support HSC maintenance and long-term repopulating capacity.

The importance of the ECM in the HSC niche, particularly under stress conditions, is increasingly being recognised^38^. Under 5-FU-induced BM suppression, HSCs near the endosteum are known to preferentially survive due to reduced apoptosis^39^. Our study found that mice lacking Itga8^+^ MSCs had lower total MNC, GMP, and MEP counts after receiving 5-FU, as well as delayed haematopoietic recovery. Additionally, deficiency of ECM components like tenascin C delays haematopoietic recovery after 5-FU treatment without affecting steady-state haematopoiesis^40^. These findings are consistent with our data and suggest that niche factors produced by Itga8^+^ MSCs, including ECM molecules, play a crucial role in HSC maintenance, especially under stress.

MSCs have diverse functions. Unlike previously reported MSCs that constitute the BM niche, Itga8^+^ MSCs secrete only modest amounts of cytokines and chemokines. Instead, they secrete abundant ECM-related molecules, such as Mfap4, which classifies them as ECM-secreting MSCs. These findings suggest that Itga8^+^ MSCs provide a structural scaffold around the endosteum via the ECM and play a part in forming a distinct niche from the vascular niche, which is rich in cytokines and chemokines.

Our study has significant implications for the ex vivo expansion of HSCs. Efficient expansion of HSCs without compromising their self-renewal and multipotency is crucial for transplantation therapy^41–43^. Given that Itga8^+^ MSCs more effectively support LTR activity than other MSCs in vitro, applying niche factors produced by Itga8^+^ MSCs into previously reported culture systems may enhance the quality and quantity of HSCs produced^44–46^. Such approaches may help to mimic the BM microenvironment more closely, potentially improving engraftment rates and haematopoietic recovery post-transplantation. Therefore, further characterisation of Itga8^+^ MSCs and identification of any additional niche molecules they express, beyond Mfap4, are needed.

In conclusion, our findings highlight the critical role of Itga8^+^ MSCs in the HSC niche and suggest that ECM-mediated interactions are key to maintaining HSC quiescence and function. Understanding the hierarchical relationships among MSC subsets and their specific contributions to the HSC niche will advance our knowledge of haematopoietic regulation and aid in developing improved strategies for HSC expansion and transplantation.

## Methods

### Ethics statement

The present study is in accordance with all pertinent ethical regulations. The study protocol was approved by the Gene Recombination Experiment Safety Committee (approval number: 5-2) and the Animal Experiment Committee (approval number: A25-209-2) of Kyushu University. All experiments were conducted in accordance with the Guidelines for Animal and Recombinant DNA Experiments at Kyushu University.

### Mice

All mice were maintained in a specific pathogen-free (SPF) environment under a 12-h light/dark cycle (lights on from 8:00 a.m. to 8:00 p.m.). The ambient temperature was regulated between 20 and 26 °C, and the relative humidity was maintained between 40 and 60%. The mice used in the experiments were 6 to 12 weeks old. C57BL/6JJcl (B6-Ly5.2) mice (6–8 weeks old) were obtained from CLEA Japan, Inc., and congenic C57BL/6 mice for the Ly5 locus (B6-Ly5.1) were purchased from Sankyo-Lab Service (Tsukuba, Japan). LepR-Cre knock-in mice were provided by Dr. Yoshihiro Baba (The University of Kyushu, Japan), and Rosa26-loxP-stop-loxP-DTR-tdTomato mice were provided by Dr. Masashi Yanagisawa (University of Tsukuba, Japan). Itga8-Cre KI mice were generated on a C57BL/6J background using CRISPR/Cas9 gene editing, where a guide RNA targeting the mouse Itga8 gene, together with a donor vector containing the IRES-Cre cassette and Cas9 mRNA, were co-injected into fertilised C57BL/6J mouse eggs to insert the IRES-Cre cassette downstream of the stop codon, producing targeted KI offspring. Rosa-DTR-tdTomato mice express tdTomato and DTR in a Cre-inducible manner. Itga8^Cre/+^; Rosa-DTR-tdTomato mice were generated by crossing Itga8-Cre KI mice with Rosa-DTR-tdTomato mice. To ablate Itga8^+^ cells, 30 µL of DT (Merck) at a concentration of 0.05 µg/mL was administered directly into the bone marrow cavity of the tibia. Genotyping for Itga8-Cre KI mice was performed using PCR with Premix Taq (Takara) under the following conditions: initial denaturation at 94 °C for 5 min; 35 cycles of 94 °C for 30 s, 57 °C for 35 s, and 72 °C for 35 s; followed by a final extension at 72 °C for 5 min and a hold at 4 °C. The primers used were as follows: forward primer (5′-GAGTTTCAATACCGGAGATCATGC-3′) and reverse primer (5′-CATGGCTGTGGATTGTTTCTGTAG-3′), which amplify a 254 bp product. In addition, Genotyping of Rosa26-loxP-stop-loxP-DTR-tdTomato mice was conducted using PCR with Quick Taq HS DyeMix (TOYOBO) under the following conditions: an initial denaturation at 94 °C for 5 min; 35 cycles consisting of denaturation at 94 °C for 30 s, annealing at 60 °C for 30 s, and extension at 68 °C for 30 s; followed by a final extension at 68 °C for 5 min and a hold at 4 °C. The primers used were forward (5′-ACGTTTCCGACTTGAGTTGC-3′) and reverse (5′-CATGTCTGAGATCCATAACTTCG-3′), amplifying a 507 bp product.

### Flow cytometry and immunohistochemical staining antibody

The following antibodies were used for flow cytometry: Anti-mouse CD45-FITC (Biolegend, Cat# 103108, Clone:30-F11, 1:200), Anti-mouse Ter119-PE (BD Biosciences, Cat# 553673, Clone:TER-119, 1:100), Anti-mouse CD31-PE-Cy7 (BD Biosciences, Cat# 561410, Clone:390, 1:100), Anti-mouse CD166(Alcam)-APC (Miltenyi biotec, Cat# 130-105-444, Clone:REA370, 10:100), Anti-mouse Itga8-Biotin (R&D, Cat# BAF4076, 2:100), Streptavidin-BV421 (BD Biosciences, Cat# 563259, 2:100), Streptavidin-APC-Cy7 (BD Biosciences, Cat# 554063, 2:100), Anti-mouse Ly-6A/E (Sca-1)-AF488 (Biolegend, Cat# 122516, Clone:E13-161.7, 1:100), Anti-mouse Ly-6A/E (Sca-1)-BV421 (BioLegend, Cat# 108127, Clone:D7, 2:100), Anti-mouse Ly-6A/E (Sca-1)-BV510 (BioLegend, Cat# 108129, Clone:D7, 3:100), Anti-mouse Ly-6A/E (Sca-1)-Biotin (Biolegend, Cat# 122504, Clone: E13-161.7, 2:100), Anti-mouse Itga8 (R&D, Cat# AF4076, 2:100), Anti-mouse CD51-APC (Bio-Rad, Cat# MCA2461A647, Clone:RMV-7, 5:100), Anti-mouse CD140a-BV421 (BD Biosciences, Cat# 562774, Clone:APA5, 3:100), Anti-mouse CD140a-Biotin (Biolegend, Cat# 135909, 2:100), LepR-Biotin (R&D, Cat# BAF497, 2:100), Anti-mouse CD4-PerCP-Cy5.5 (Biolegend, Cat# 100540, Clone:RM4-5, 1:200), Anti-mouse CD4-PE (BD Biosciences, Cat# 553049, Clone:RM4-5, 1:200), Anti-mouse CD8a-PerCP-Cy5.5 (Biolegend, Cat# 100734, Clone:53-6.7, 1:200), Anti-mouse CD8a-PE (BD Biosciences, Cat# 553033, Clone:53-6.7, 1:200), Anti-mouse CD45R/B220-PerCP-Cy5.5 (Biolegend, Cat# 103236, Clone:RA3-6B2, 1:200), Anti-mouse CD45R/B220-PE (BD Biosciences, Cat# 553090, Clone:RA3-6B2, 1:200), Anti-mouse Ter119-PerCP-Cy5.5 (Biolegend, Cat# 116228, Clone:TER-119, 1:200), Anti-mouse Ter119-PE (BD Biosciences, Cat# 553673, Clone:TER-119, 1:200), Anti-mouse Ter119-BV421 (Biolegend, Cat# 116234, Clone:TER-119, 2:100), Anti-mouse Granulocyte receptor 1 (Gr-1) (Ly-6G/Ly-6C)-PerCP-Cy5.5 (Biolegend, Cat# 116211, Clone:RB6-8C5, 1:200), Anti-mouse Gr-1(Ly-6G/Ly-6C)-PE (BD Biosciences, Cat# 561084, Clone:RB6-8C5, 1:200), Anti-mouse CD11b(Mac-1)-PerCP-Cy5.5 (Biolegend, Cat# 101228, Clone:M1/70, 1:200), Anti-mouse CD11b(Mac-1)-PE (BD Biosciences, Cat# 553311, Clone:M1/70, 1:200), Anti-mouse CD117(c-Kit)-APC (Biolegend, Cat# 105812, Clone:2B8, 5:100), CD117 MicroBeads, mouse (Miltenyi Biotec, Cat# 130-091-224, 10:100), Anti-mouse CD150(SLAM)-PE-Cy7 (Biolegend, Cat# 115914, Clone:TC15-12F12.2, 5:100), Anti-mouse CD48-BV510 (BD Biosciences, Cat# 563536, Clone:HN48-1, 3:100), Anti-mouse CD135 (FMS-like tyrosine kinase 3, Flt3)-BV421 (BD Biosciences, Cat# 562898, Clone:A2F10.1, 3:100), Anti-mouse CD34-Biotin (Thermo Fisher Scientific, Cat# 13-0341-85, 4:100), Anti-mouse CD16/32-APC-Cy7 (BD Biosciences, Cat# 553776, Clone:93, 2:100), Anti-mouse CD127(IL-7Ra)-PE-Cy7 (Biolegend, Cat# 101327, Clone:A7R34, 5:100), Anti-mouse CD45.1-PE (Tonbo Biosciences, Cat# 60-1271, Clone:A20, 1:100), Anti-mouse CD45.1-AF488 (Biolegend, Cat# 110718, Clone:A20, 1:100), Anti-mouse CD45.2-FITC (BD Biosciences, Cat# 553772, Clone:104, 1:100), Anti-mouse CD45.2-APC (BD Biosciences, Cat# 553776, Clone:104, 1:100), CD45 MicroBeads, mouse (Miltenyi Biotec, Cat# 130-052-301, 5:100), Anti-Ter-119 MicroBeads, mouse (Miltenyi Biotec, Cat# 130-049-901, 5:100), Anti-Annexin Ⅴ-APC (BioLegend, Cat# 640920, 5:100). The Annexin Ⅴ Binding Buffer (BioLegend, Cat# 422201) was used for Annexin Ⅴ staining. The antibodies used for immunohistochemical staining are listed below: Anti-mouse Alcam (GeneTex, Cat# GTX130098, 1:250), Anti-mouse CD45-APC (BD Biosciences, Cat# 561018, Clone:30-F11, 5:100), Anti-mouse CD4-APC (Biolegend, Cat# 100516, Clone:RM4-5, 2:100), Anti-mouse CD8a-APC (Biolegend, Cat# 100712, Clone:53-6.7, 2:100), Anti-mouse CD45R/B220-APC (Tonbo Biosciences, Cat# 20-0452, Clone:RA3-6B2, 2:100), Anti-mouse Ter119-APC (Biolegend, Cat# 116211, Clone:TER-119, 2:100), Anti-mouse Gr-1(Ly-6G/Ly-6C)-APC (BD Biosciences, Cat# 553129, Clone:RB6-8C5, 2:100), Anti-mouse CD11b(Mac-1)-APC (Biolegend, Cat# 101212, Clone:M1/70, 2:100), Anti-mouse CD150(SLAM)-PE (Biolegend, Cat# 115904, Clone:TC15-12F12.2, 5:100), Anti-mouse CD48-APC (Biolegend, Cat# 103412, Clone:HN48-1, 5:100), Anti-mouse CD41-APC (Biolegend, Cat# 133914, Clone:MWReg30, 2:100), Donkey anti-Goat IgG (H + L) Highly Cross-Adsorbed Secondary Antibody, Alexa Fluor™ Plus 405 (Thermo Fisher Scientific, Cat# A48259, 1:200), Donkey anti-Rabbit IgG (H + L) Highly Cross-Adsorbed Secondary Antibody, Alexa Fluor™ 488 (Thermo Fisher Scientific, Cat# A-21206, 1:200), Streptavidin, Alexa Fluor™ 555 Conjugate (Thermo Fisher Scientific, Cat# S32355, 1:200), MFAP4 Polyclonal Antibody (Thermo Fisher Scientific, Cat# PA5-42013, 1:250).

### TaqMan probe for qPCR

The following TaqMan probes were used for qPCR analysis: Actb (Thermo Fisher Scientific, Mm00607939_s1), Alcam (Thermo Fisher Scientific, Mm00711623_m1), Alpl (Thermo Fisher Scientific, Mm00475834_m1), Angpt1 (Thermo Fisher Scientific, Mm00456503_m1), Anpep (Thermo Fisher Scientific, Mn00476227-m1), Bglap1 (Thermo Fisher Scientific, Mm03413826_m1), Bmpr1a (Thermo Fisher Scientific, Mm00477650_m1), Bmpr1b (Thermo Fisher Scientific, Mm03023971_m1), Cd109 (Thermo Fisher Scientific, Mm00462151_m1), Cd151 (Thermo Fisher Scientific, Mm01275496_g1), Cd248 (Thermo Fisher Scientific, Mm00547485_s1), Cd276 (Thermo Fisher Scientific, Mm00506020_m1), Cd44 (Thermo Fisher Scientific, Mm01277160_m1), Cd63 (Thermo Fisher Scientific, Mm01966817_m1), Cd81 (Thermo Fisher Scientific, Mm00504869_m1), Cd9 (Thermo Fisher Scientific, Mm00514275_m1), Cdh11 (OB-cad) (Thermo Fisher Scientific, Mm00515462_m1), Cdh2 (N-cad) (Thermo Fisher Scientific, Mm00483213_m1), Cdh5 (VE-cad) (Thermo Fisher Scientific, Mm00486938_m1), Col1a1 (Thermo Fisher Scientific, Mm01302043_g1), Cxcl12 (Thermo Fisher Scientific, Mm00445553_m1), Cxcr4 (Thermo Fisher Scientific, Mm01302043_g1), Ednrb (Thermo Fisher Scientific, Mm00432989_m1), Egfr (Thermo Fisher Scientific, Mm00433023_m1), Eng (Thermo Fisher Scientific, Mm00468256_m1), Epcam (Thermo Fisher Scientific, Mm00493214_m1), Erbb2 (Thermo Fisher Scientific, Mm00658541_m1), Fgfr1 (Thermo Fisher Scientific, Mm00438923_m1), Fgfr2 (Thermo Fisher Scientific, Mm01275521_m1), Fgfr3 (Thermo Fisher Scientific, Mm00433294_m1), Fgfr4 (Thermo Fisher Scientific, Mm01341852_m1), Fgfrl1 (Thermo Fisher Scientific, Mm00475318_g1), Fut4 (Thermo Fisher Scientific, Mm00487448_s1), Fzd9 (Thermo Fisher Scientific, Mm01206511_s1), Gabrb3 (Thermo Fisher Scientific, Mm00433473_m1), Gapdh (Thermo Fisher Scientific, Mm99999915_g1), Gata6 (Thermo Fisher Scientific, Mm00802636_m1), Gfap (Thermo Fisher Scientific, Mm01253033_m1), Gypa (Thermo Fisher Scientific, Mm00494848_m1), Igfr1 (Thermo Fisher Scientific, Mm00802831_m1), Igfr2 (Thermo Fisher Scientific, Mm01320054_m1), Itga2b (Thermo Fisher Scientific, Mm00439768_m1), Itga2 (Thermo Fisher Scientific, Mm00434371_m1), Itga4 (Thermo Fisher Scientific, Mm00439770_m1), Itga6 (Thermo Fisher Scientific, Mm00434375_m1), Itga8 (Thermo Fisher Scientific, Mm01324958_m1), Itga9 (Thermo Fisher Scientific, Mm00519293_m1), Itgb2 (Thermo Fisher Scientific, Mm00434513_m1), Itgal (Thermo Fisher Scientific, Mm00801807_m1), Itgav (Thermo Fisher Scientific, Mm00434506_m1), Itgb1 (Thermo Fisher Scientific, Mm01253227_m1), Itgb3 (Thermo Fisher Scientific, Mm00443980_m1), Jag1 (Thermo Fisher Scientific, Mm00496921_g1), Jag2 (Thermo Fisher Scientific, Mm00439953_g1), kit (Thermo Fisher Scientific, Mm00726565_m1), Kitl (Thermo Fisher Scientific, Mm00442972_m1), Klf4 (Thermo Fisher Scientific, Mm00516104_m1), Lepr (Thermo Fisher Scientific, Mm00440181_m1), Lif (Thermo Fisher Scientific, Mm00434762_g1), Lifr (Thermo Fisher Scientific, Mm00442942_m1), Ly6a (Thermo Fisher Scientific, Mm00726565_s1), Mcam (Thermo Fisher Scientific, Mm00522397_m1), Met (Thermo Fisher Scientific, Mm01156972_m1), Mfap4 (Thermo Fisher Scientific, Mm00840681_m1), Mme (Thermo Fisher Scientific, Mm01285049_m1), Mpl (Thermo Fisher Scientific, Mm00440310_m1), Nanog (Thermo Fisher Scientific, Mm01617762_g1), Ncam (Thermo Fisher Scientific, Mm01149710_m1), Ngfr (Thermo Fisher Scientific, Mm01309638_m1), Notch1 (Thermo Fisher Scientific, Mm00435249_m1), Notch2 (Thermo Fisher Scientific, Mm00803077_m1), Nrpl (Thermo Fisher Scientific, Mm00435379_m1), Nt5e (Thermo Fisher Scientific, Mm00501910_m1), Ntrk3 (Thermo Fisher Scientific, Mm00456222_m1, Pdgfra (Thermo Fisher Scientific, Mm00440701_m1), Pdgfrb (Thermo Fisher Scientific, Mm01262489_m1), Pecam1 (Thermo Fisher Scientific, Mm01242584_m1), Palur (Thermo Fisher Scientific, Mm00440911_m1), Pou5f1 (Thermo Fisher Scientific, Mm00658129_gH), Prm1 (Thermo Fisher Scientific, Mm01342731_g1), Ptprc (Thermo Fisher Scientific, Mm01293575_m1), Ret (Thermo Fisher Scientific, Mm00436304_m1), Runx2 (Thermo Fisher Scientific, Mm00501578_m1), Sema4a (Thermo Fisher Scientific, Mm00443140_m1), Sox2 (Thermo Fisher Scientific, Mm00488369_s1), Sp7 (Thermo Fisher Scientific, Mm00504574_m1), Spp1 (Thermo Fisher Scientific, Mm01204014_m1), Tgfr1 (Thermo Fisher Scientific, Mm00436964_m1), Tgfr2 (Thermo Fisher Scientific, Mm03024091_m1), Thy1 (Thermo Fisher Scientific, Mm00493681_m1), Vcam1 (Thermo Fisher Scientific, Mm01320970_m1).

### Flow cytometry analysis

Cells were suspended in ice-cold PBS containing 0.5% bovine serum albumin (BSA) (Nacalai Tesque). Antibodies were added to the cell suspension at the proportions described above, and the cells were incubated on ice for 30 min. The samples were then washed twice with the same buffer and resuspended in the same buffer. PI was added to the cell suspension at final concentration of 1 μg/ml to exclude dead cells. The cell suspension was filtered through a 40 μm cell strainer prior to analysis. Flow cytometric analysis was performed using a FACSAria III or FACSAria Fusion (BD Biosciences) equipped with FACSDiva software. Data were analysed using FlowJo software (BD Biosciences).

### BLC isolation

Endosteal cells were isolated using the previously described method with minor modifications^26^. In brief, femora, tibias and iliac bones were fragmented into small pieces (approximately 1–2 mm) after flushing out the BM cells. The bone fragments were incubated at 37 °C for 90 min in collagenase solution containing type I collagenase (2.5 mg/mL, Worthington Biochemical), 20% fetal calf serum (FCS) and 250 units of DNase (Sigma) in DMEM. After the incubation, cells were treated with ACK lysing buffer (160 mM ammonium chloride, 10 mM potassium bicarbonate, 0.1 mM EDTA) to lyse red blood cells, and collected BLCs. BLCs were stained with anti-CD45, -Ter119, -CD31, -Alcam, -Sca-1, -Itga8 antibodies and propidium iodide (PI), and were analysed and sorted using FACS Aria III (BD Biosciences) and FACS Aria Fusion (BD Biosciences).

### CD51^+^CD140a^+^ MSC isolation

BM flushed out from the femora and the tibia was digested with Accumax at 26 °C for 60 min. After collection of the cells, red blood cells were lysed by treatment with ACK lysing buffer. The stromal cells were stained with anti-CD45, -Ter119, -CD31, -CD51, -CD140a antibodies and PI, and CD51^+^CD140a^+^ MSCs in CD45^**−**^ Ter119^**−**^ CD31^**−**^ cells were sorted using FACS Aria III and FACS Aria Fusion.

### Immunohistochemical staining

Femora were fixed with 4% paraformaldehyde (PFA, Electron Microscopy Sciences) in phosphate-buffered saline (PBS) at 4 °C overnight. After fixation, bones were decalcified with Decalcifying Solution B (FUJIFILM Wako) at 4 °C for 5 days. For the preparation of the frozen section, the decalcified femora were embedded in OCT compound (SAKURA) and frozen at −100 °C in UT2000F cooling unit (Tokyo Rikakikai, Co., Ltd; Tokyo, Japan). The frozen samples were sectioned with Cryostar NX70 (Thermo Fisher Scientific) at 10 μm sections and mounted on MAS-coated glass slides (Matsunami Glass Ind., Osaka, Japan). The tissue sections were incubated with Protein Block (Dako) at 4 °C for 1 h, and subsequently with Avidin/Biotin Blocking Kit (vector laboratories) at 4 °C for 30 min. Primary antibodies were applied to the tissue sections at 4 °C overnight. After washing in PBS, the sections were incubated with fluorescence secondary antibodies at room temperature for 1 h. Confocal imaging was performed using a BZ-X700 fluorescence microscope (KEYENCE) and an LSM 780 confocal microscope (Zeiss). The images obtained from the LSM 780 confocal microscope were analysed using ZEN 3.3 blue edition (Zeiss).

### Osteo-, adipo- and chondrogenic differentiation assay

To assess the differentiation potential into osteoblasts and adipocytes, cells were cultured on type I collagen-coated 48-well plates (Corning) in Complete StemXVivo Osteogenic/Adipogenic Base Media (R&D) for 6 days. For osteogenic differentiation, cells were subsequently cultured in Complete StemXVivo Osteogenic Differentiation Media (R&D) for an additional 14 days, and differentiation was evaluated via ALP staining. For adipogenic differentiation, cells were subsequently cultured in Complete StemXVivo Adipogenic Differentiation Media (R&D) for 21 days, and differentiation was assessed by the accumulation of lipid vesicles using Oil Red O staining.

To assess chondrogenic differentiation potential, cells were initially cultured in type I collagen-coated 6-well plates (Corning) in Mesencult Medium (STEMCELL Technologies) for 7 days. Cells were then harvested with 0.05% Trypsin-EDTA (Thermo Fisher Scientific) and pelleted by centrifugation. Cell pellets were cultured in Complete StemXVivo Chondrogenic Differentiation Media (R&D) for 28 days. Chondrogenic differentiation was evaluated using Alcian Blue staining.

ImageJ software was used to quantify staining by applying a threshold to the image to select only the stained areas, then measuring their area and intensity.

### Coculture of LSK cells with stromal cells

Itga8^+^ MSCs, Itga8^−^Alcam^+^ BLCs, Sca-1^+^ MSCs, and CD51^+^CD140a^+^ MSCs were seeded on type I collagen-coated 96-well plates (Corning) and cultured in MesenCult Basal Medium (STEMCELL Technologies) for 4 days to reach confluence. LSK cells (1 × 10^4^ cells/well) were then cocultured on each mesenchymal stromal cell type in MyeloCult H5100 Medium (STEMCELL Technologies) without cytokine supplementation. After 2 days of coculture, cells were collected to analyse HSC frequency and function.

### Coculture experiments using Transwell^®^ inserts

Itga8^+^ MSCs (8000 cells/well) were seeded onto type I collagen-coated 12-well plates (Corning) in MesenCult Basal Medium and cultured for 6 days. After this period, LSK cells (20,000 cells/well) were added to the wells with or without Transwell® inserts (0.4 µm pore size, transparent PET membrane, Corning) in MyeloCult H5100 Medium without cytokine supplementation for an additional 2 days. After coculture, cells were analysed and sorted for subsequent bone marrow transplantation.

### Culture of LSK cells with Mfap4 protein

LSK cells (1 × 10^4^ cells/well) were cultured in MyeloCult H5100 medium in fibronectin-coated 96-well plates for 2 days. Mfap4 protein (MedChemExpress) was added to the wells at the concentrations of 0, 0.25, 0.50, 1.25, 2.50 and 5.00 μg/mL.

### BMT assay

In coculture experiments involving LSK cells (CD45.1^+^) with mesenchymal cells, as well as in experiments using Transwell^®^ inserts, CD45.1^+^Lin^−^ cells were isolated after coculture and transplanted into lethally irradiated (9.5 Gy) CD45.2^+^ recipient mice along with CD45.2^+^ competitor bone marrow (BM) cells. For the BMT assay following depletion of Itga8^+^ MSCs, LSK cells were isolated from Itga8^Cre/+^; Rosa-DTR-tdTomato mice (CD45.1^+^CD45.2^+^) treated with DT or control. These cells were then transplanted into CD45.2+ recipient mice together with CD45.2^+^ competitor BM cells.

To evaluate the function of Mfap4, LSK cells (CD45.1^+^) were cultured with Mfap4 for 2 days without additional cytokines. After this culture period, CD45.1^+^Lin^−^ cells (200 cells/mice) were isolated and transplanted into CD45.2^+^ recipient mice with CD45.2^+^ competitor BM cells. The frequency of donor-derived cells in PB was assessed monthly, and the frequency of donor-derived HSCs in BM LSK cells was analysed four months after transplantation.

### qPCR

Total RNA was isolated using the QIAshredder and RNeasy Micro Kit (Qiagen). cDNA synthesis was carried out with the Transcriptor High Fidelity cDNA Synthesis Kit (Roche). qPCR reactions were performed using the TaqMan Fast Universal PCR Master Mix (Thermo Fisher Scientific). qPCR analysis was conducted on the CFX384 Touch Real-Time PCR System (Bio-Rad) and the Applied Biosystems QuantStudio 3 (Thermo Fisher Scientific). Expression levels were normalised to β-actin mRNA. Data were analysed with CFX Manager Software v2.1 (Bio-Rad) and QuantStudio™ Design and Analysis Software v1.5.2 (Thermo Fisher Scientific).

### RNA-seq analysis

The isolation of mRNA was performed using Nucleo Spin RNA Plus XS (Takara) from 2000 cells each of Itga8^+^ cells, Itga8^−^ cells, Sca-1^+^ cells and CD51^+^ CD140a^+^ cells. The cDNA synthesis was performed using the SMART-Seq v4 Ultra Low Input RNA Kit for Sequencing (Takara). The cDNA library was prepared using Nextera XT DNA Library Preparation Kits (Illumina) and assessed for quality using Agilent 2100 Bioanalyzer (Agilent) and Agilent’s High Sensitivity DNA Kit (Agilent). The library was sequenced using the NextSeq 500 system (Illumina) and mapped to the mouse genome (mm10) using TopHat/Bowtie2. Analysis of differentially expressed genes and the creation of PCA plots and K-means clustering were performed using iDEP 2.01.

### Single cell qPCR analysis

The procedure for the single-cell qPCR array (96.96 Dynamic Array, Standard BioTools) was described previously^26^.

### scRNA-seq

scRNA-seq was performed on FACS-sorted Alcam⁺Sca-1^−^ BLCs. Single-cell RNA-seq libraries were prepared using the Chromium Next GEM Single Cell 3ʹ Reagent Kit v3.1 (10× Genomics, Pleasanton, CA) according to the manufacturer’s protocol. Approximately 10,000 cells were loaded into each channel of a Chromium Next GEM Chip G, with an expected recovery of 7000–8000 single cells per sample. Libraries were sequenced on the Illumina NovaSeq 6000 platform using paired-end sequencing (28 bp for Read 1 and 91 bp for Read 2) with dual 10-bp i7 and i5 index reads, yielding approximately 20,000 read pairs per cell. FASTQ files were aligned to the mouse reference genome (mm10), and gene–barcode matrices were generated using Cell Ranger v7.1.0 (10× Genomics). Data from three independent Alcam⁺Sca-1^−^ BLC samples (total *n* = 25,704 cells) were aggregated using the cellranger aggr pipeline with default depth normalization to generate an integrated feature–barcode matrix. Graph-based clustering and UMAP visualization were then performed in Loupe Browser v8.0.0 using default settings.

### Statistical analysis

Statistical analyses were carried out using GraphPad Prism software (version 8.0). Data in all bar graphs are presented as the mean ± standard deviation (SD). A two-tailed paired Student’s *t*-test was used to compare two groups, and one-way ANOVA was used for comparisons among three or more independent groups. All statistical tests were two-sided. Significant *p*-values were defined as follows: ns, not significant; *p* < 0.05; *p* < 0.01; *p < 0.001; **p < 0.0001.

### Reporting summary

Further information on research design is available in the Nature Portfolio Reporting Summary linked to this article.

## Supplementary information

Supplementary Information

Peer Review file

Reporting Summary

## Source data

Source data

## Data Availability

All raw and processed RNA sequencing data generated in this study have been deposited in the Gene Expression Omnibus (GEO) under accession number GSE280158 and GSE326153. All data supporting the findings of this study are available within the paper and its Extended Data files, or are provided as a Source Data file. Therefore, no data are subject to restriction or require separate request from the authors. Source data are provided with this paper.

## References

[1] Laurenti, E. & Göttgens, B. From haematopoietic stem cells to complex differentiation landscapes. *Nature* **553**, 418–426 (2018). 29364285 10.1038/nature25022 PMC6555401

[2] Seita, J. & Weissman, I. L. Hematopoietic stem cell: self-renewal versus differentiation. *Wiley Interdiscip. Rev. Syst. Biol. Med.* **2**, 640–653 (2010). 20890962 10.1002/wsbm.86 PMC2950323

[3] Morrison, S. J. & Scadden, D. T. The bone marrow niche for haematopoietic stem cells. *Nature* **505**, 327–334 (2014). 24429631 10.1038/nature12984 PMC4514480

[4] Pinho, S. & Frenette, P. S. Haematopoietic stem cell activity and interactions with the niche. *Nat. Rev. Mol. Cell Biol.* **20**, 303–320 (2019). 30745579 10.1038/s41580-019-0103-9 PMC6483843

[5] Mendelson, A. & Frenette, P. S. Hematopoietic stem cell niche maintenance during homeostasis and regeneration. *Nat. Med.* **20**, 833–846 (2014). 25100529 10.1038/nm.3647 PMC4459580

[6] Sugiyama, T., Kohara, H., Noda, M. & Nagasawa, T. Maintenance of the hematopoietic stem cell pool by CXCL12-CXCR4 chemokine signaling in bone marrow stromal cell niches. *Immunity* **25**, 977–988 (2006). 17174120 10.1016/j.immuni.2006.10.016

[7] Ding, L., Saunders, T. L., Enikolopov, G. & Morrison, S. J. Endothelial and perivascular cells maintain haematopoietic stem cells. *Nature* **481**, 457–462 (2012). 22281595 10.1038/nature10783 PMC3270376

[8] Arai, F. et al. Tie2/angiopoietin-1 signaling regulates hematopoietic stem cell quiescence in the bone marrow niche. *Cell* **118**, 149–161 (2004). 15260986 10.1016/j.cell.2004.07.004

[9] Yoshihara, H. et al. Thrombopoietin/MPL signaling regulates hematopoietic stem cell quiescence and interaction with the osteoblastic niche. *Cell Stem Cell* **1**, 685–697 (2007). 18371409 10.1016/j.stem.2007.10.020

[10] Himburg, H. A. et al. Distinct bone marrow sources of pleiotrophin control hematopoietic stem cell maintenance and regeneration. *Cell Stem Cell* **23**, 370–381.e5 (2018). 30100167 10.1016/j.stem.2018.07.003 PMC6482945

[11] Kiel, M. J. et al. SLAM family receptors distinguish hematopoietic stem and progenitor cells and reveal endothelial niches for stem cells. *Cell* **121**, 1109–1121 (2005). 15989959 10.1016/j.cell.2005.05.026

[12] Kunisaki, Y. et al. Arteriolar niches maintain haematopoietic stem cell quiescence. *Nature* **502**, 637–643 (2013). 24107994 10.1038/nature12612 PMC3821873

[13] Calvi, L. M. et al. Osteoblastic cells regulate the haematopoietic stem cell niche. *Nature* **425**, 841–846 (2003). 14574413 10.1038/nature02040

[14] Zhou, B. O., Yue, R., Murphy, M. M., Peyer, J. G. & Morrison, S. J. Leptin-receptor-expressing mesenchymal stromal cells represent the main source of bone formed by adult bone marrow. *Cell Stem Cell* **15**, 154–168 (2014). 24953181 10.1016/j.stem.2014.06.008 PMC4127103

[15] Butler, J. M. et al. Endothelial cells are essential for the self-renewal and repopulation of Notch-dependent hematopoietic stem cells. *Cell Stem Cell* **6**, 251–264 (2010). 20207228 10.1016/j.stem.2010.02.001 PMC2866527

[16] Méndez-Ferrer, S. et al. Mesenchymal and haematopoietic stem cells form a unique bone marrow niche. *Nature* **466**, 829–834 (2010). 20703299 10.1038/nature09262 PMC3146551

[17] Bruns, I. et al. Megakaryocytes regulate hematopoietic stem cell quiescence through CXCL4 secretion. *Nat. Med.* **20**, 1315–1320 (2014). 25326802 10.1038/nm.3707 PMC4258871

[18] Zhao, M. et al. Megakaryocytes maintain homeostatic quiescence and promote post-injury regeneration of hematopoietic stem cells. *Nat. Med.* **20**, 1321–1326 (2014). 25326798 10.1038/nm.3706

[19] Winkler, I. G. et al. Bone marrow macrophages maintain hematopoietic stem cell (HSC) niches and their depletion mobilizes HSCs. *Blood* **116**, 4815–4828 (2010). 20713966 10.1182/blood-2009-11-253534

[20] Morikawa, S. et al. Prospective identification, isolation, and systemic transplantation of multipotent mesenchymal stem cells in murine bone marrow. *J. Exp. Med.* **206**, 2483–2496 (2009). 19841085 10.1084/jem.20091046 PMC2768869

[21] Zhang, J. et al. Identification of the haematopoietic stem cell niche and control of the niche size. *Nature* **425**, 836–841 (2003). 14574412 10.1038/nature02041

[22] Wilson, A. et al. Hematopoietic stem cells reversibly switch from dormancy to self-renewal during homeostasis and repair. *Cell* **135**, 1118–1129 (2008). 19062086 10.1016/j.cell.2008.10.048

[23] Yu, V. W. C. et al. Specific bone cells produce DLL4 to generate thymus-seeding progenitors from bone marrow. *J. Exp. Med.* **212**, 759–774 (2015). 25918341 10.1084/jem.20141843 PMC4419348

[24] Yu, V. W. C. et al. Distinctive mesenchymal-parenchymal cell pairings govern B cell differentiation in the bone marrow. *Stem Cell Rep.* **7**, 220–235 (2016). 10.1016/j.stemcr.2016.06.009 PMC4982987 27453006

[25] Zhu, J. et al. Osteoblasts support B-lymphocyte commitment and differentiation from hematopoietic stem cells. *Blood* **109**, 3706–3712 (2007). 17227831 10.1182/blood-2006-08-041384

[26] Nakamura, Y. et al. Isolation and characterization of endosteal niche cell populations that regulate hematopoietic stem cells. *Blood* **116**, 1422–1432 (2010). 20472830 10.1182/blood-2009-08-239194

[27] Hooper, A. T. et al. Engraftment and reconstitution of hematopoiesis is dependent on VEGFR2-mediated regeneration of sinusoidal endothelial cells. *Cell Stem Cell* **4**, 263–274 (2009). 19265665 10.1016/j.stem.2009.01.006 PMC3228275

[28] Takakura, N. et al. PDGFR alpha expression during mouse embryogenesis: immunolocalization analyzed by whole-mount immunohistostaining using the monoclonal anti-mouse PDGFR alpha antibody APA5. *J. Histochem. Cytochem.* **45**, 883–893 (1997). 9199674 10.1177/002215549704500613

[29] Pinho, S. et al. PDGFRα and CD51 mark human nestin+ sphere-forming mesenchymal stem cells capable of hematopoietic progenitor cell expansion. *J. Exp. Med.* **210**, 1351–1367 (2013). 23776077 10.1084/jem.20122252 PMC3698522

[30] Iwasaki, K. et al. Ablation of central serotonergic neurons decreased REM sleep and attenuated arousal response. *Front. Neurosci.* **12**, 535 (2018). 30131671 10.3389/fnins.2018.00535 PMC6090062

[31] Kanaan, R., Medlej-Hashim, M., Jounblat, R., Pilecki, B. & Sorensen, G. L. Microfibrillar-associated protein 4 in health and disease. *Matrix Biol.* **111**, 1–25 (2022). 35644509 10.1016/j.matbio.2022.05.008

[32] Ong, S. L. M., de Vos, I. J. H. M., Meroshini, M., Poobalan, Y. & Dunn, N. R. Microfibril-associated glycoprotein 4 (Mfap4) regulates haematopoiesis in zebrafish. *Sci. Rep.* **10**, 11801 (2020). 32678226 10.1038/s41598-020-68792-8 PMC7366704

[33] de Pater, E. et al. Gata2 is required for HSC generation and survival. *J. Exp. Med.* **210**, 2843–2850 (2013). 24297996 10.1084/jem.20130751 PMC3865477

[34] North, T. E., Stacy, T., Matheny, C. J., Speck, N. A. & de Bruijn, M. F. T. R. Runx1 is expressed in adult mouse hematopoietic stem cells and differentiating myeloid and lymphoid cells, but not in maturing erythroid cells. *Stem Cells* **22**, 158–168 (2004). 14990855 10.1634/stemcells.22-2-158

[35] Hynes, R. O. Integrins: bidirectional, allosteric signaling machines. *Cell* **110**, 673–687 (2002). 12297042 10.1016/s0092-8674(02)00971-6

[36] Cheng, T., Rodrigues, N., Dombkowski, D., Stier, S. & Scadden, D. T. Stem cell repopulation efficiency but not pool size is governed by p27(kip1). *Nat. Med.* **6**, 1235–1240 (2000). 11062534 10.1038/81335

[37] Khurana, S. et al. Outside-in integrin signalling regulates haematopoietic stem cell function via Periostin-Itgav axis. *Nat. Commun.* **7**, 13500 (2016). 27905395 10.1038/ncomms13500 PMC5146274

[38] Lee-Thedieck, C., Schertl, P. & Klein, G. The extracellular matrix of hematopoietic stem cell niches. *Adv. Drug Deliv. Rev.* **181**, 114069 (2022). 34838648 10.1016/j.addr.2021.114069 PMC8860232

[39] Zhao, M. et al. N-cadherin-expressing bone and marrow stromal progenitor cells maintain reserve hematopoietic stem cells. *Cell Rep.* **26**, 652–669.e6 (2019). 30650358 10.1016/j.celrep.2018.12.093 PMC6890378

[40] Nakamura-Ishizu, A. et al. Extracellular matrix protein tenascin-C is required in the bone marrow microenvironment primed for hematopoietic regeneration. *Blood* **119**, 5429–5437 (2012). 22553313 10.1182/blood-2011-11-393645 PMC3418770

[41] Copelan, E. A. Hematopoietic stem-cell transplantation. *N. Engl. J. Med.* **354**, 1813–1826 (2006). 16641398 10.1056/NEJMra052638

[42] Kumar, S. & Geiger, H. HSC niche biology and HSC expansion ex vivo. *Trends Mol. Med.* **23**, 799–819 (2017). 28801069 10.1016/j.molmed.2017.07.003 PMC5600322

[43] Tajer, P., Pike-Overzet, K., Arias, S., Havenga, M. & Staal, F. J. T. Ex vivo expansion of hematopoietic stem cells for therapeutic purposes: lessons from development and the niche. *Cells* **8**, 169 (2019). 30781676 10.3390/cells8020169 PMC6407064

[44] Wilkinson, A. C. et al. Long-term ex vivo haematopoietic-stem-cell expansion allows nonconditioned transplantation. *Nature* **571**, 117–121 (2019). 31142833 10.1038/s41586-019-1244-x PMC7006049

[45] Sakurai, M. et al. Chemically defined cytokine-free expansion of human haematopoietic stem cells. *Nature* **615**, 127–133 (2023). 36813966 10.1038/s41586-023-05739-9

[46] Bai, T. et al. Expansion of primitive human hematopoietic stem cells by culture in a zwitterionic hydrogel. *Nat. Med.* **25**, 1566–1575 (2019). 31591594 10.1038/s41591-019-0601-5

