# Supplementary material for: Endosteal integrin-α8⁺ mesenchymal stem cells maintain haematopoietic stem cell function via extracellular matrix-mediated interactions
Source: Nat Commun. 2026 Jul 6;17:8355. doi: 10.1038/s41467-026-75276-2 (PMC13473570; doi:10.1038/s41467-026-75276-2)
Supplement: Supplementary file 1 [file 41467_2026_75276_MOESM1_ESM.pdf]

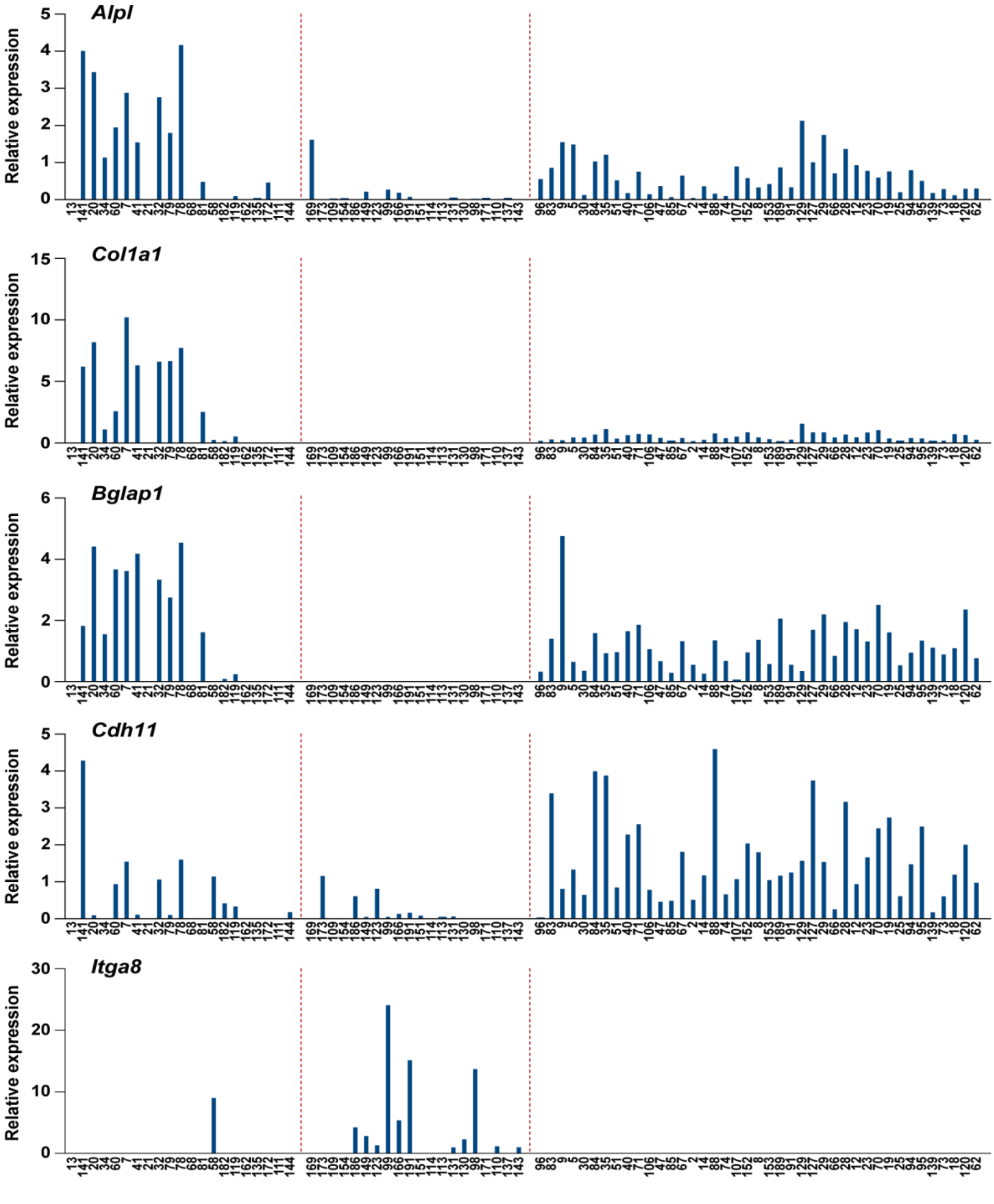


**Extended Data Fig. 1**

**Extended Data Figure 1. Itga8 marks a distinct subpopulation within Alcam⁺Sca-1⁻ BLCs.**

Single-cell qPCR analysis of *Alpl*, *Bglap1*, *Col1a1*, *Cdh11* and *Itga8* in Alcam⁺Sca-1⁻ BLCs (82 cells in total). *Itga8* was expressed in a distinct subset of Alcam⁺Sca-1⁻ BLCs with little or no expression of osteoblastic markers.

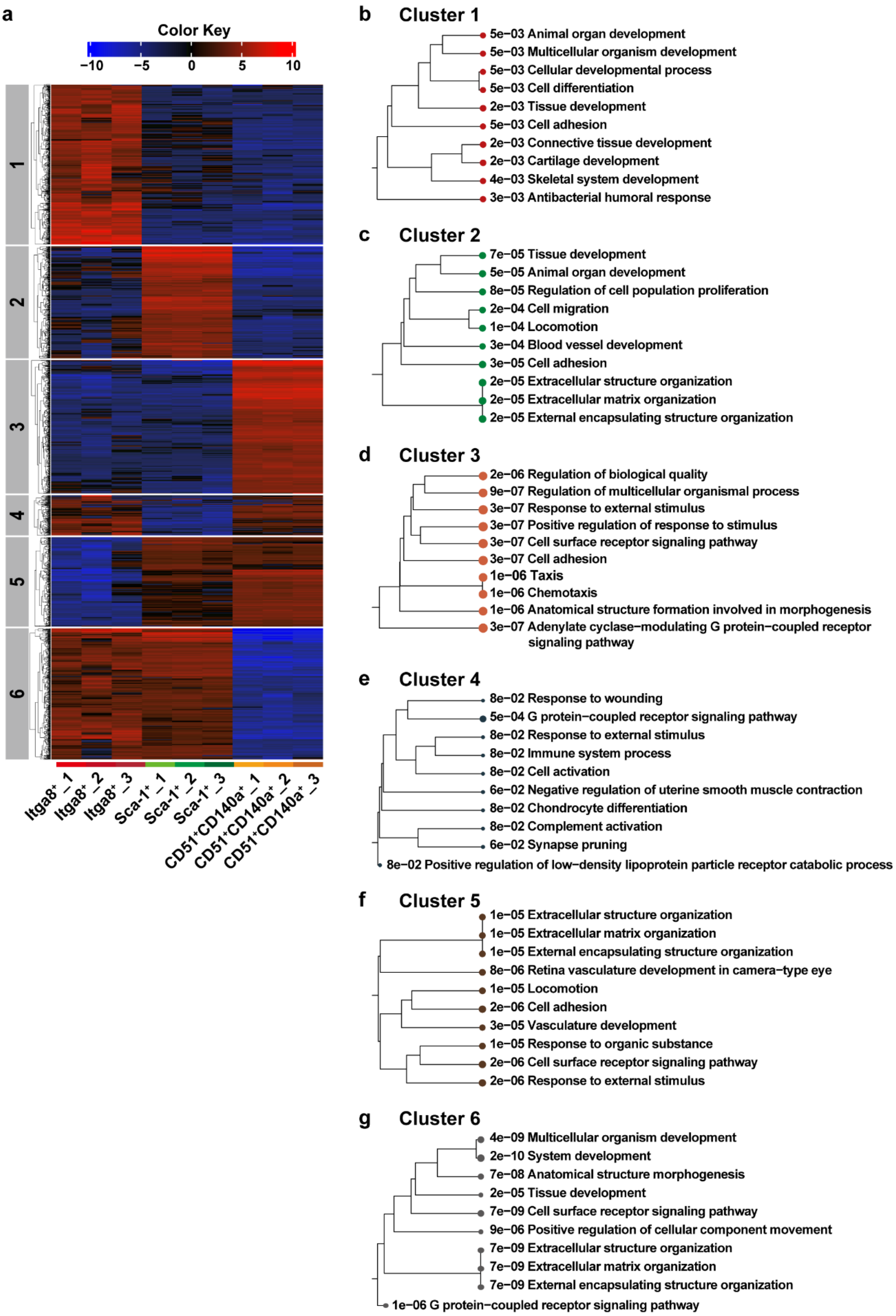


**Extended Data Fig. 2**

**Extended Data Figure 2. Gene expression profiles of Itga8$^+$ MSCs, Sca-1$^+$ MSCs, and CD51$^+$CD140a$^+$ MSCs.**

**(a)** K-means clustering of the 2,000 most variable genes in Itga8$^+$ MSCs, Sca-1$^+$ MSCs, and CD51$^+$CD140a$^+$ MSCs, revealing six distinct clusters.

**(b)** Enriched pathways in Cluster 1.

**(c)** Enriched pathways in Cluster 2.

**(d)** Enriched pathways in Cluster 3.

**(e)** Enriched pathways in Cluster 4.

**(f)** Enriched pathways in Cluster 5.

**(g)** Enriched pathways in Cluster 6.

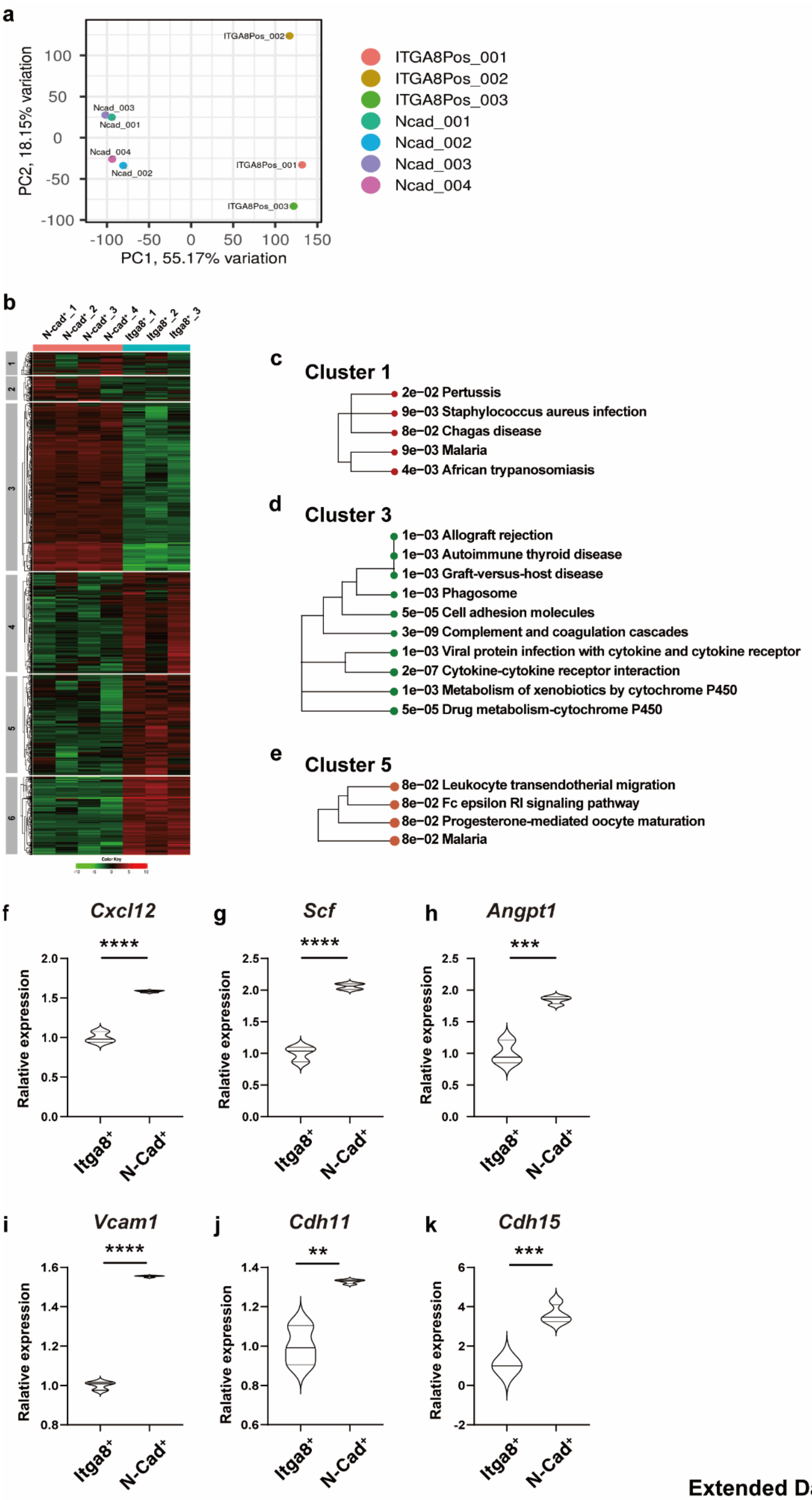


**Extended Data Fig. 3**

**Extended Data Figure 3. Gene expression profiles distinguish N-cadherin$^+$ and Itga8$^+$ MSCs in HSC niche regulation.**

**(a)** PCA plot comparing transcriptomic profiles of N-cadherin$^+$ and Itga8$^+$ MSCs.

**(b)** K-means clustering of the 2,000 most variable genes in N-cadherin$^+$ MSCs and Itga8$^+$ MSCs, revealing six distinct clusters.

**(c)** Enriched pathways in Cluster 1.

**(d)** Enriched pathways in Cluster 3.

**(e)** Enriched pathways in Cluster 5.

**(f–k)** Comparison of the expression of HSC niche factors (*Cxcl12*, *Scf*, *Angpt1, Vcam1, Cdh11, and Cdh15*) in N-cadherin$^+$ MSCs, and Itga8$^+$ MSCs. **(f)** *Cxcl12*, **(g)** *Scf*, **(h)** *Angpt1*, **(i)** *Vcam1*, **(j)** *Cdh11*, **(k)** *Cdh15*. Statistical significance was determined by a two-sided unpaired t-test. $^{**}p < 0.01$, $^{***}p < 0.001$, $^{****}p < 0.0001$. **(f)** exact $p = 1.3 \times 10^{-5}$. **(g)** exact $p = 1.6 \times 10^{-5}$. **(h)** exact $p = 0.0003$. **(i)** exact $p = 4.9 \times 10^{-8}$. **(j)** exact $p = 0.0011$. **(k)** exact $p = 0.0003$.

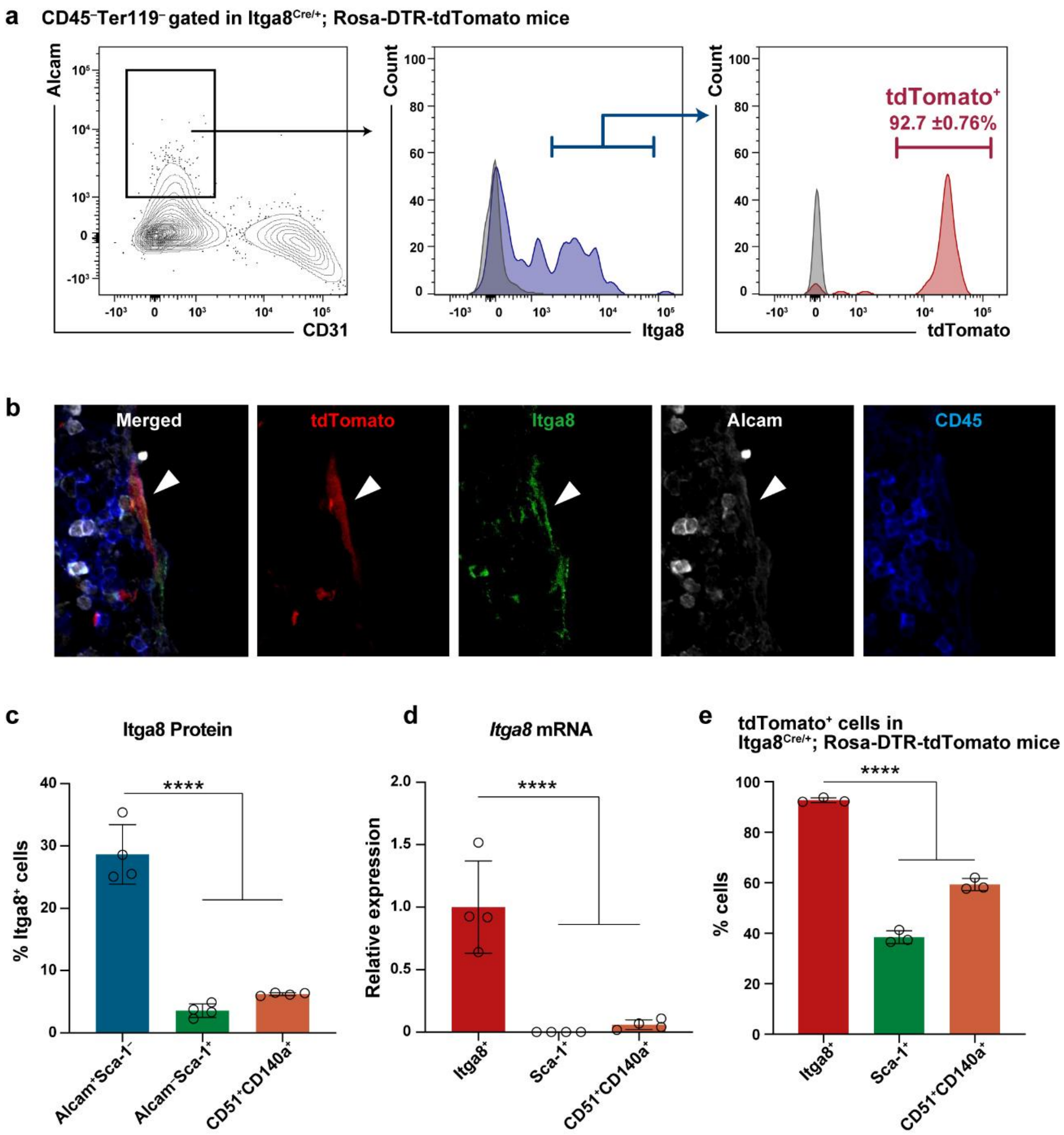


**Extended Data Fig. 4**

**Extended Data Figure 4. Expression of Itga8 in BM MSC subsets and BLCs.**

**(a)** Flow cytometric analysis showing tdTomato expression in $Itga8^{+}Alcam^{+}$ cells in $Itga8^{Cre/+}$; Rosa-DTR-tdTomato mice. A representative flow cytometry plot is shown.

**(b)** Enlarged views of the endosteal BM panel shown in Fig. 3c. $Itga8^{+}tdTomato^{+}Alcam^{+}$ MSCs are indicated by arrowheads. Scale bars, 20 μm.

**(c)** Flow cytometric analysis of the frequency of $Itga8^{+}$ cells in $Alcam^{+}Sca\text{-}1^{-}$ BLCs, $Alcam^{-}Sca\text{-}1^{+}$ MSCs and $CD51^{+}CD140a^{+}$ MSCs (n = 4/group).

**(d)** qPCR analysis of Itga8 mRNA expression in $Itga8^{+}Alcam^{+}Sca\text{-}1^{-}$ MSCs, $Alcam^{-}Sca\text{-}1^{+}$ MSCs and $CD51^{+}CD140a^{+}$ MSCs (n = 4/group).

**(e)** The percentage of $tdTomato^{+}$ cells among $Itga8^{+}$, $Sca\text{-}1^{+}$ and $CD51^{+}CD140a^{+}$ MSCs in $Itga8^{Cre/+}$; Rosa-DTR-tdTomato mice, indicating cells with a history of Itga8 expression.

Data are presented as mean ± SD. Statistical differences in (c–e) were determined by ordinary one-way ANOVA followed by Tukey's multiple-comparison test. ****$p < 0.0001$. **(c)** $Alcam^{+}Sca\text{-}1^{-}$ vs $Alcam^{-}Sca\text{-}1^{+}$: exact $p = 3.6 \times 10^{-6}$. $Alcam^{+}Sca\text{-}1^{-}$ vs $CD51^{+}CD140a^{+}$MSCs: exact $p = 1.4 \times 10^{-6}$. **(d)** $Alcam^{+}Sca\text{-}1^{-}$ vs $Alcam^{-}Sca\text{-}1^{+}$: exact $p = 2.6 \times 10^{-4}$. $Alcam^{+}Sca\text{-}1^{-}$ vs $CD51^{+}CD140a^{+}$MSCs: exact $p = 4.1 \times 10^{-4}$. **(e)** $Alcam^{+}Sca\text{-}1^{-}$ vs $Alcam^{-}Sca\text{-}1^{+}$: exact $p = 2.7 \times 10^{-7}$. $Alcam^{+}Sca\text{-}1^{-}$ vs $CD51^{+}CD140a^{+}$MSCs: exact $p = 2.6 \times 10^{-6}$.

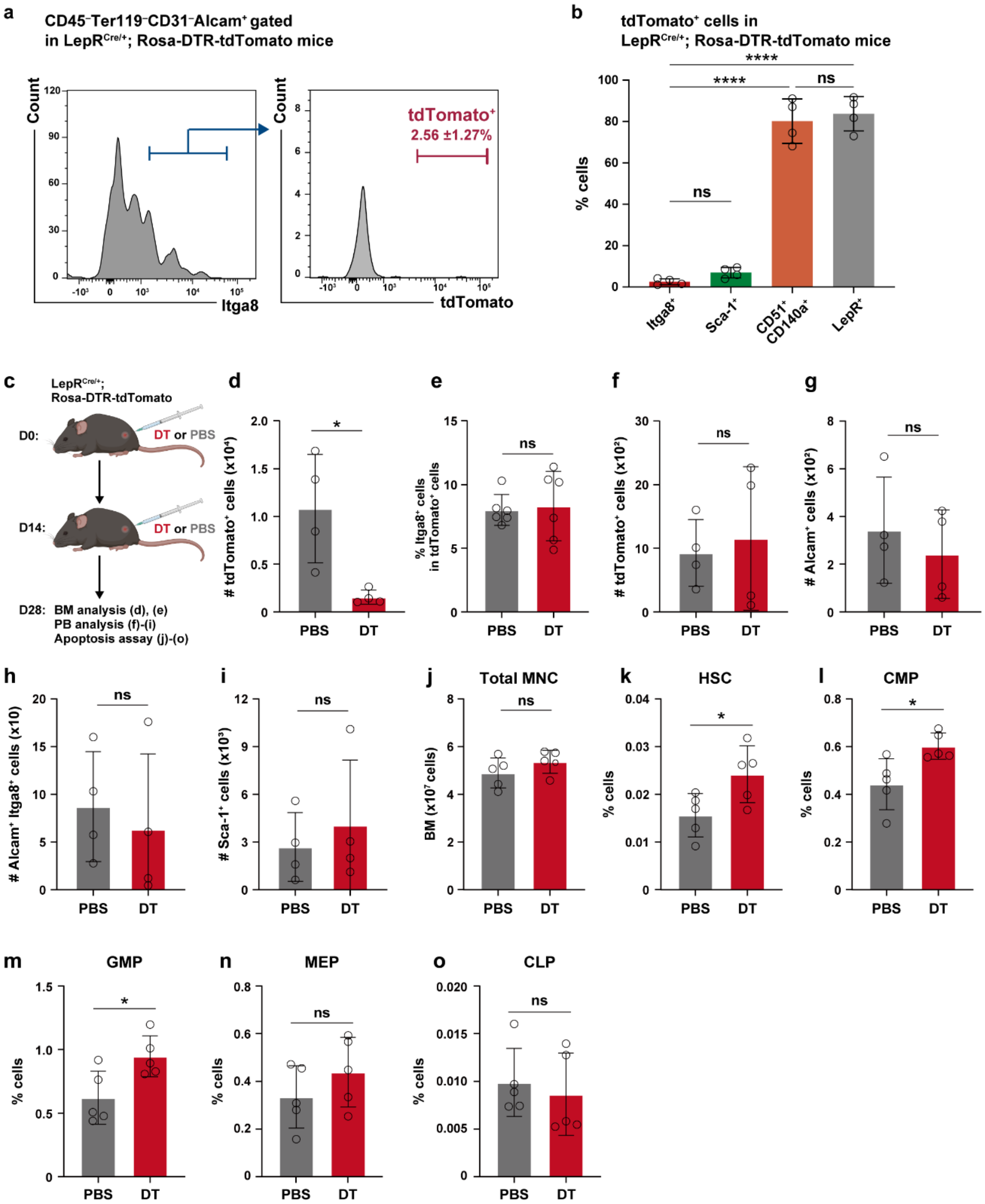


Extended Data Fig. 5

**Extended Data Figure 5. Depletion of LepR$^{+}$ MSCs does not affect endosteal cells.**

**(a)** Flow cytometric analysis showing tdTomato expression in Itga8$^{+}$Alcam$^{+}$ MSCs in LepR$^{Cre/+}$; Rosa-DTR-tdTomato mice. A representative flow cytometry plot is shown.

**(b)** Frequency of tdTomato$^{+}$ cells in Itga8$^{+}$ MSCs, Sca-1$^{+}$ MSCs, CD51$^{+}$CD140a$^{+}$ MSCs and LepR$^{+}$ MSCs in LepR$^{Cre/+}$; Rosa-DTR-tdTomato mice.

**(c)** Schematic representation of the experimental design for analysis following depletion of LepR$^{+}$ cells by DT administration in LepR$^{Cre/+}$; Rosa-DTR-tdTomato mice. Mice were administered DT or PBS on day 0 and day 14. Fourteen days after the final treatment, BM MSCs, BLCs and BM cells were analysed.

**(d–i)** Flow cytometry analysis of central BM-MSCs and BLCs in LepR$^{Cre/+}$; Rosa-DTR-tdTomato mice after DT or control (PBS) treatment (n = 4/group). **(d)** The number of tdTomato$^{+}$ cells in central BM. **(e)** The proportion of Itga8$^{+}$ cells among tdTomato$^{+}$ cells in central BM. **(f)** The number of tdTomato$^{+}$ cells in BLCs. **(g)** The number of Alcam$^{+}$ cells in BLCs. **(h)** The number of Alcam$^{+}$Itga8$^{+}$ cells in BLCs. **(i)** The number of Sca-1$^{+}$ cells in BLCs.

**(j–o)** Flow cytometry analysis of BM haematopoietic cells in LepR$^{Cre/+}$; Rosa-DTR-tdTomato mice after DT or control treatment (n = 5/group). **(j)** The total number of mononuclear cells (MNCs) in the BM. **(k)** The proportion of HSCs (LSKCD48$^{-}$CD150$^{+}$). **(l)** The proportion of CMPs (LSKCD16/32$^{-}$CD34$^{+}$). **(m)** The proportion of GMPs (LSKCD16/32$^{+}$CD34$^{+}$). **(n)** The proportion of MEPs (LSKCD16/32$^{-}$CD34$^{-}$). **(o)** The proportion of CLPs (CD127$^{+}$Lin$^{-}$Sca-1$^{low}$c-kit$^{low}$).

Statistical differences in (b) were determined by ordinary one-way ANOVA followed by Tukey's multiple-comparison test. Statistical differences in (d–o) were determined by an unpaired two-tailed Student's t-test. *$p < 0.05$, ****$p < 0.0001$; ns, not significant. (b) Itga8$^{+}$ vs

CD51$^+$CD140a$^+$: exact $p = 1.1 \times 10^{-8}$. Itga8$^+$ vs LepR$^+$: exact $p = 6.7 \times 10^{-9}$. **(d)** exact $p = 0.0175$. **(k)** exact $p = 0.0338$. **(l)** exact $p = 0.0180$. **(m)** exact $p = 0.0245$. Created in BioRender. Arai, F. (2026) https://BioRender.com/jykdosl.

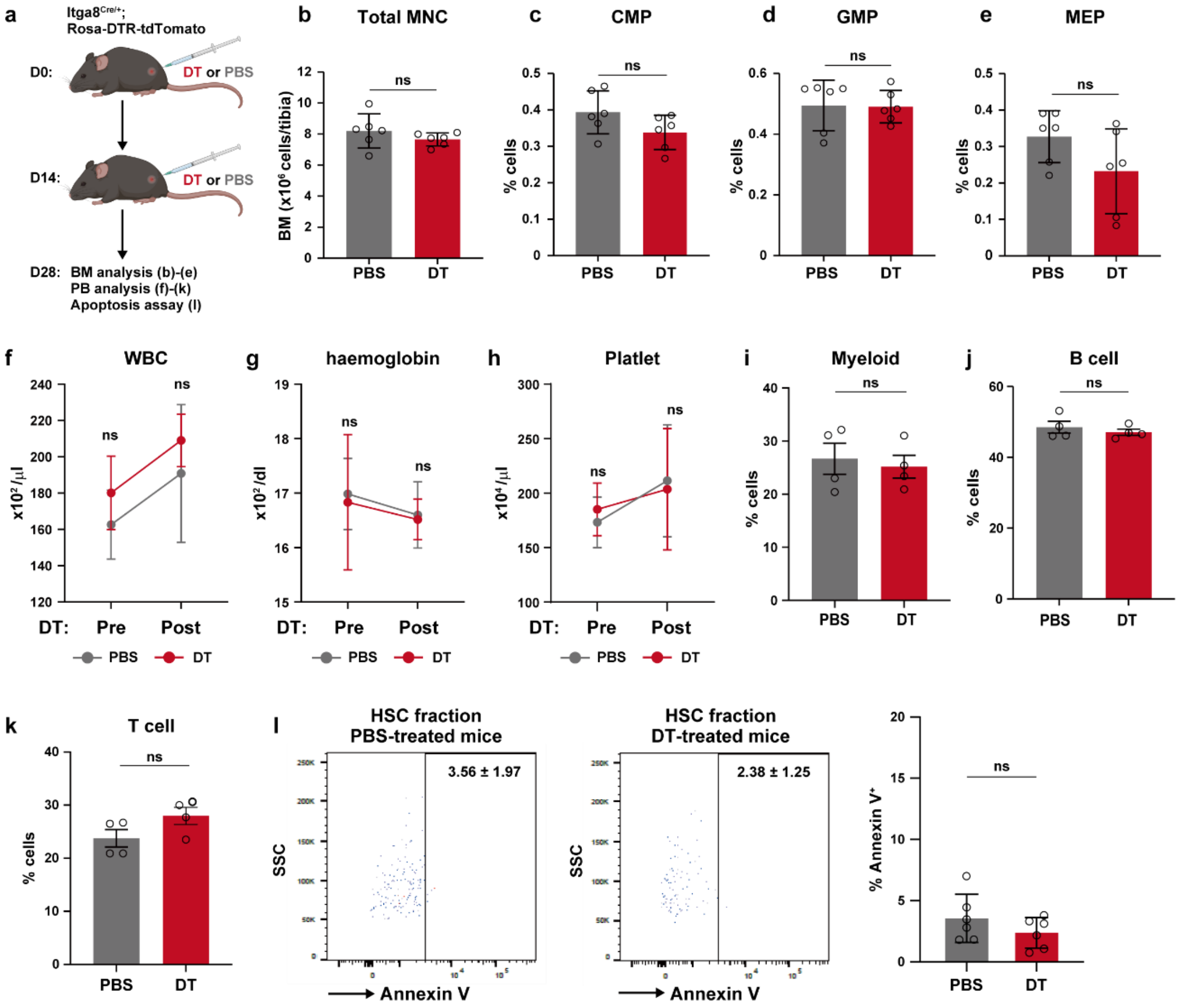


Extended Data Fig. 6

**Extended Data Figure 6. Depletion of Itga8+ MSCs does not affect haematopoietic progenitor cells or mature haematopoietic cells.**

**(a)** Schematic representation of flow cytometric analysis following depletion of $Itga8^{+}$ cells by DT administration in $Itga8^{Cre/+}$; Rosa-DTR-tdTomato mice. Mice were administered DT or PBS on day 0 and day 14. Fourteen days after the final treatment, haematopoietic progenitor cells in the BM and mature haematopoietic cells in the PB were analysed.

**(b)** Total number of BM MNCs.

**(c)** The percentage of common myeloid progenitors (CMPs; $CD16/32^{-}CD34^{+}Lin^{-}Sca\text{-}1^{-}c\text{-}Kit^{+}$).

**(d)** The percentage of granulocyte-macrophage progenitors (GMPs; $CD16/32^{+}CD34^{+}Lin^{-}Sca\text{-}1^{-}c\text{-}Kit^{+}$).

**(e)** The percentage of megakaryocyte-erythroid progenitors (MEPs; $CD16/32^{-}CD34^{-}Lin^{-}Sca\text{-}1^{-}c\text{-}Kit^{+}$).

**(f)** White blood cell counts in PB.

**(g)** Haemoglobin level in PB.

**(h)** Platelet counts in PB.

**(i)** The percentage of myeloid cells ($Gr\text{-}1^{+}/Mac\text{-}1^{+}$).

**(j)** The percentage of B cells ($B220^{+}$).

**(k)** The percentage of T cells ($CD4^{+}/CD8a^{+}$).

**(l)** Flow cytometric analysis showing the presence of Annexin V-positive cells in HSCs in $Itga8^{Cre/+}$; Rosa-DTR-tdTomato mice (left). A representative flow cytometry plot is shown. Quantification of Annexin V-positive HSCs in $Itga8^{Cre/+}$; Rosa-DTR-tdTomato mice (right).

Data are presented as mean ± SD (n = 4 or 6/group), and statistical differences were determined

by an unpaired two-tailed Student’s t-test: ns, not significant. Created in BioRender. Arai, F. (2026) https://BioRender.com/jykdosl.

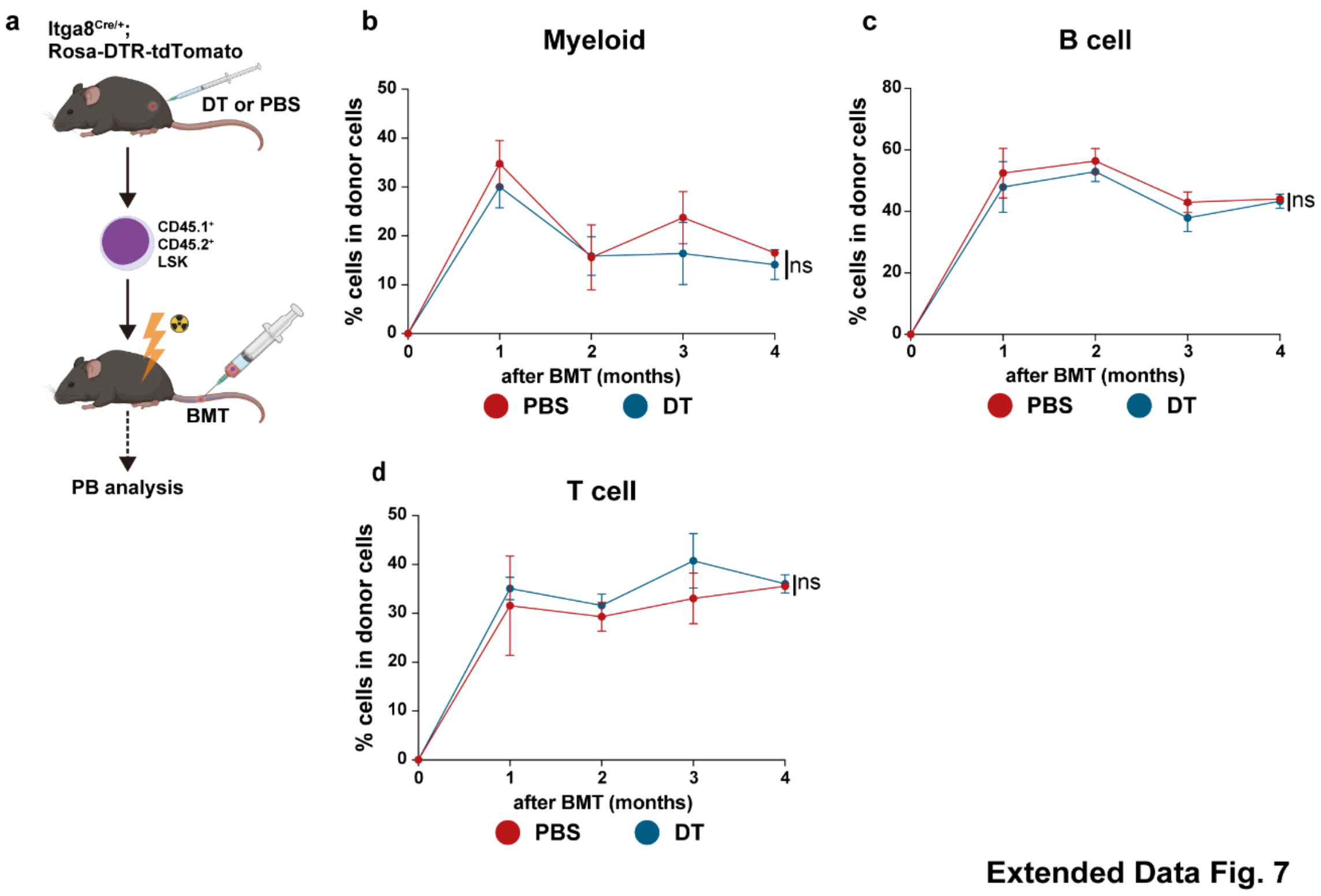


**Extended Data Figure 7. LSK cells from Itga8$^+$ MSC-depleted BM show comparable differentiation into myeloid, B and T lineages after BMT.**

**(a)** Schematic representation of PB analysis following BMT after depletion of Itga8$^+$ cells by DT administration in Itga8$^{Cre/+}$; Rosa-DTR-tdTomato mice.

**(b)** The percentage of donor-derived myeloid cells.

**(c)** The percentage of donor-derived B cells.

**(d)** The percentage of donor-derived T cells.

Data are presented as mean ± SD (n = 4 /group). Statistical differences were determined by an unpaired two-tailed Student's t-test: ns, not significant. Created in BioRender. Arai, F. (2026) https://BioRender.com/jykdosl, https://BioRender.com/xghxfih, and https://BioRender.com/ykyzx28.

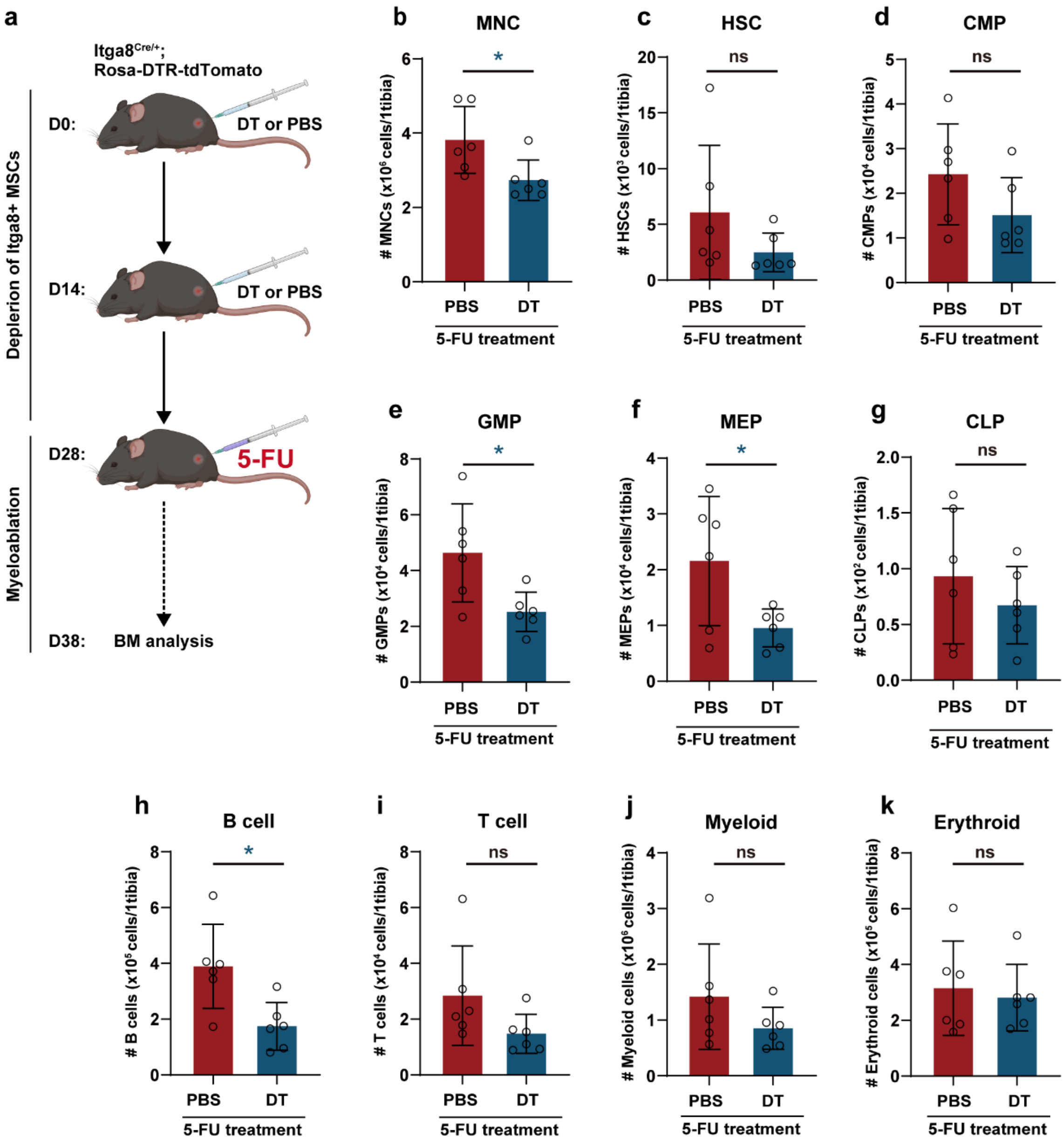


**Extended Data Fig. 8**

**Extended Data Figure 8. Haematopoietic recovery is delayed following depletion of Itga8+ MSCs under stress induced by 5-FU administration.**

**(a)** Schematic representation of the experimental design following depletion of Itga8$^{+}$ cells by DT administration and subsequent 5-FU treatment in Itga8$^{Cre/+}$; Rosa-DTR-tdTomato mice. Mice were administered DT or PBS on days 0 and 14, and 5-FU was administered 14 days after the final DT treatment. Haematopoietic cells in the BM were analysed 10 days after 5-FU administration.

**(b–k)** Quantification of HSPCs and haematopoietic lineage-positive cells in the BM of Itga8Cre/+; Rosa-DTR-tdTomato mice 10 days after 5-FU treatment. (b) Total MNCs. (c) HSCs. (d) CMPs. (e) GMPs. (f) MEPs. (g) CLPs. (h) B cells. (i) T cells. (j) Myeloid cells. (k) Erythroid cells.

Data are presented as mean ± SD (n = 6 /group). Statistical differences were determined by an unpaired two-tailed Student's t-test. *$p < 0.05$; ns, not significant. **(b)** exact p = 0.0303. **(e)** exact p = 0.0210. **(f)** exact p = 0.0351. **(h)** exact p = 0.0125. Created in BioRender. Arai, F. (2026) https://BioRender.com/jykdosl.

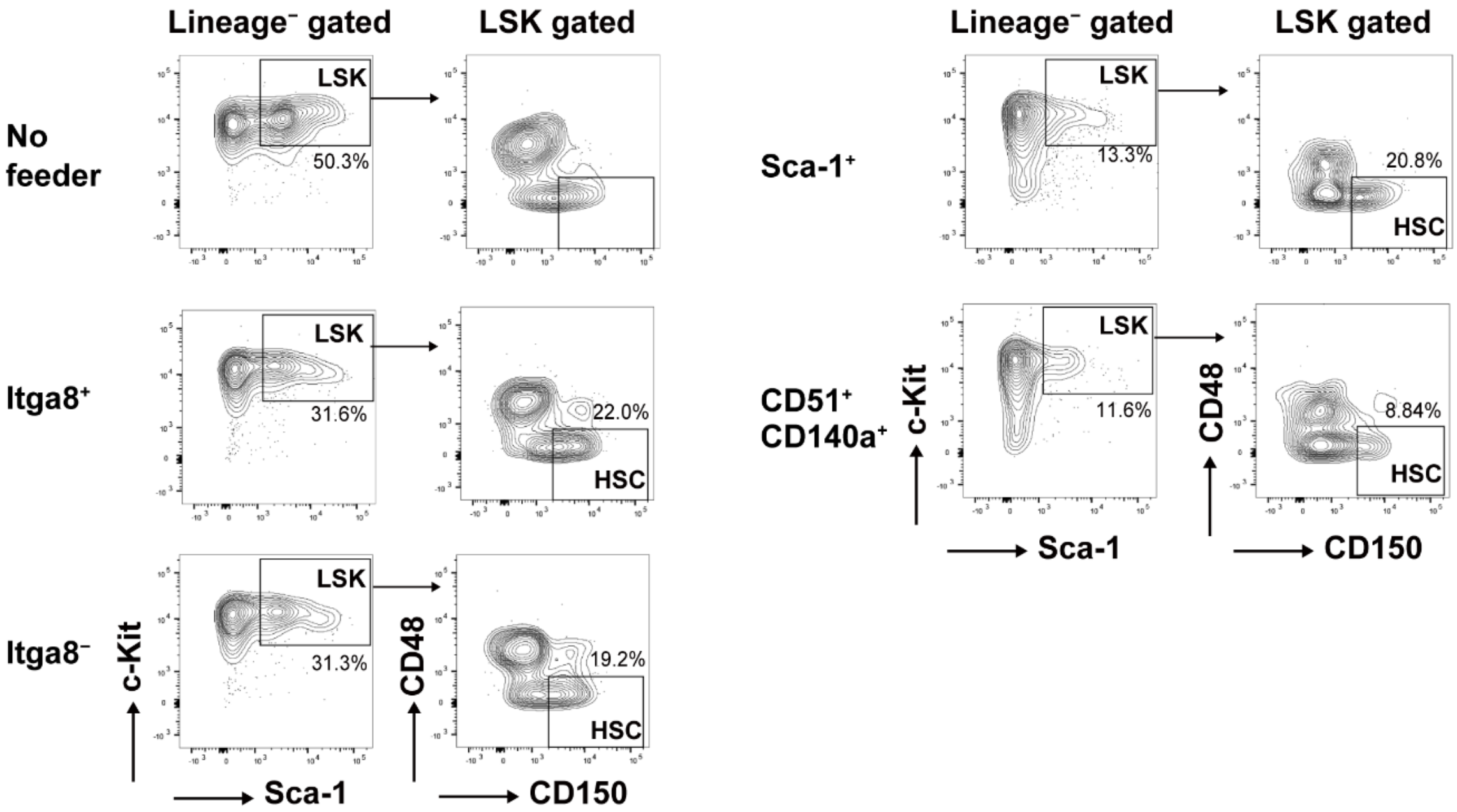


Extended Data Fig. 9

**Extended Data Figure 9. Itga8$^+$ MSCs support HSC maintenance more efficiently than other niche cells.**

Representative flow cytometry plots of LSK and LSKCD48$^-$CD150$^+$ HSCs following coculture of LSK cells with Itga8$^+$ MSCs, Itga8$^-$Alcam$^+$Sca-1$^-$ BLCs, Sca-1$^+$ MSCs and CD51$^+$CD140a$^+$ MSCs.

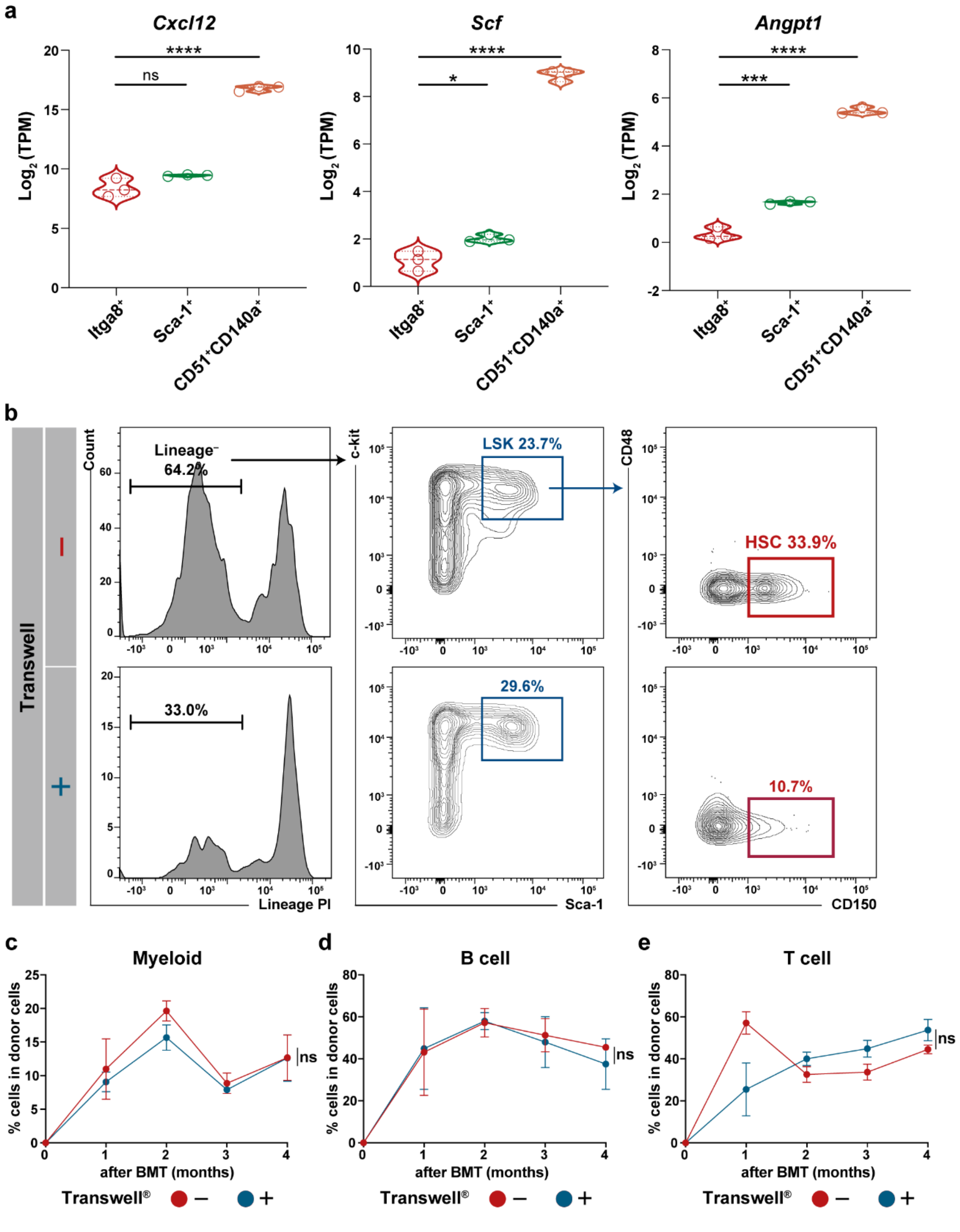


Extended Data Fig. 10

**Extended Data Figure 10. Coculture with Transwell inserts reduces the haematopoietic support capacity of Itga8$^+$ MSCs.**

**(a)** Comparison of TPM-based expression of HSC niche factors (*Cxcl12*, *Scf* and *Angpt1*) in Itga8$^+$ MSCs, Sca-1$^+$ MSCs and CD51$^+$CD140a$^+$ MSCs.

**(b)** Representative flow cytometry plots showing Lineage$^-$, LSK and LSKCD48$^-$CD150$^+$ HSC fractions following coculture with Itga8$^+$ MSCs in the presence or absence of Transwell inserts.

**(c)** The percentage of donor-derived myeloid cells after 1–4 months of BMT (n = 3 / group).

**(d)** The percentage of donor-derived B cells after 1–4 months of BMT (n = 3 / group).

**(e)** The percentage of donor-derived T cells after 1–4 months of BMT (n = 3 / group).

Data are presented as mean ± SD. Statistical differences in **(a)** were determined by ordinary one-way ANOVA followed by Tukey's multiple-comparison test. Statistical differences in **(c–e)** were determined by an unpaired two-tailed Student's t-test. *$p < 0.05$, ***$p < 0.001$, ****$p < 0.0001$; ns, not significant. **(a)** (Cxcl12) Itga8$^+$ vs CD51$^+$CD140a$^+$: exact $p = 1.5 \times 10^{-6}$. (Scf) Itga8$^+$ vs Sca-1$^+$: exact $p = 0.0197$. Itga8$^+$ vs CD51$^+$CD140a$^+$: exact $p = 2.4 \times 10^{-7}$. (Angpt1) Itga8$^+$ vs Sca-1$^+$: exact $p = 0.00021$. Itga8$^+$ vs CD51$^+$CD140a$^+$: exact $p = 1.0 \times 10^{-7}$.

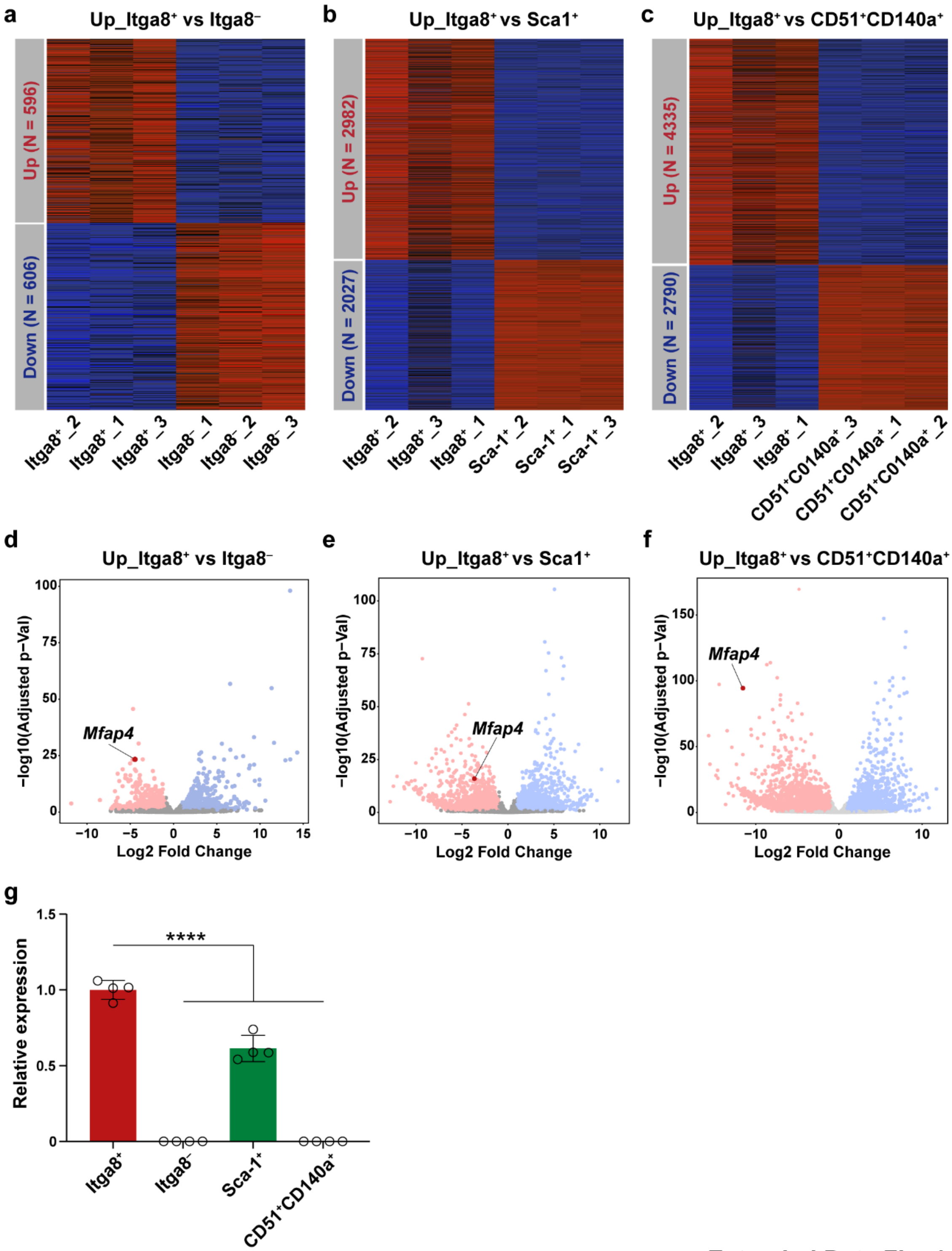


**Extended Data Fig. 11**

**Extended Data Figure 11. Mfap4 is highly expressed in Itga8$^+$ MSCs.**

**(a–c)** Heatmaps showing DEGs in RNA-seq data (false discovery rate (FDR) < 0.1, minimum fold change > 2). **(a)** Comparison between Itga8$^+$ MSCs and Itga8$^-$Alcam$^+$ BLCs. **(b)** Comparison between Itga8$^+$ MSCs and Sca-1$^+$ MSCs. **(c)** Comparison between Itga8$^+$ MSCs and CD51$^+$CD140a$^+$ MSCs.

**(d–f)** Volcano plots showing expression changes (log2 fold change) and statistical significance (−log10 adjusted P value) of genes in the RNA-seq data. **(d)** Comparison between Itga8$^+$ MSCs and Itga8$^-$Alcam$^+$ BLCs. **(e)** Comparison between Itga8$^+$ MSCs and Sca-1$^+$ MSCs. **(f)** Comparison between Itga8$^+$ MSCs and CD51$^+$CD140a$^+$ MSCs.

**(g)** Expression of *Mfap4* in Itga8$^+$ MSCs, Itga8$^-$Alcam$^+$ BLCs, Sca-1$^+$ MSCs and CD51$^+$CD140a$^+$ MSCs (n = 4/group).

Data are presented as mean ± SD. Statistical differences were determined by ordinary one-way ANOVA followed by Tukey's multiple-comparison test. ****$p < 0.0001$. **(g)** Itga8$^+$ vs Itga8$^-$: exact $p = 1.4 \times 10^{-11}$. Itga8$^+$ vs Sca-1$^+$: exact $p = 1.4 \times 10^{-6}$. Itga8$^+$ vs CD51$^+$CD140a$^+$: exact $p = 1.4 \times 10^{-11}$.

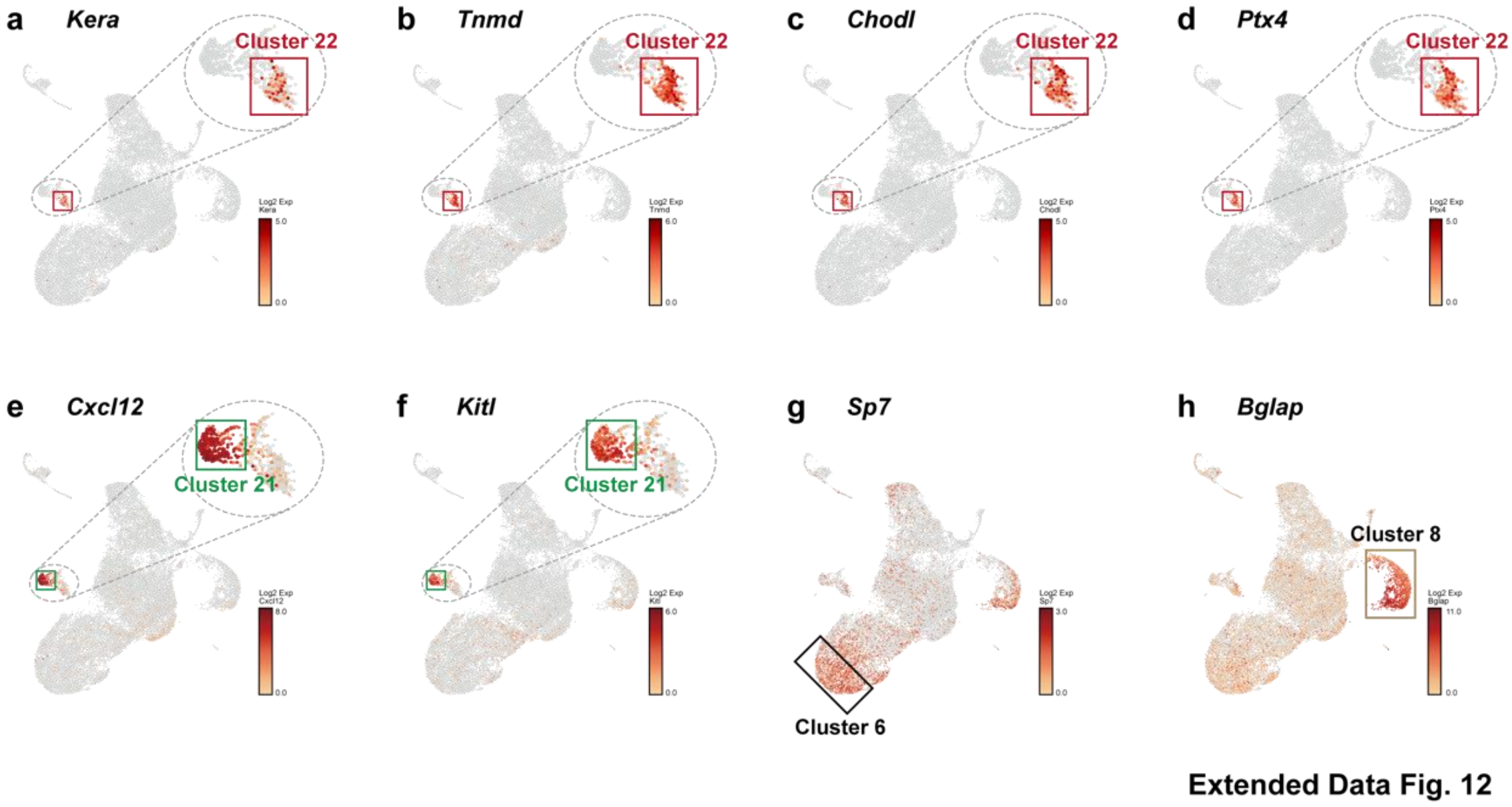


**Extended Data Figure 12. scRNA-seq analysis reveals distinct mesenchymal cell populations within Alcam$^{+}$ BLCs.**

**(a–h)** UMAP feature plots showing the expression of selected genes in Alcam$^{+}$ BLCs: **(a)** *Kera*, **(b)** *Tnmd*, **(c)** *Chodl*, **(d)** *Ptx4*, **(e)** *Cxcl12*, **(f)** *Kitl*, **(g)** *Sp7* and **(h)** *Bglap*. Each dot represents a single cell, and the colour indicates log-normalised expression level. In addition to *Mfap4* shown in Fig. 6j, *Kera*, *Tnmd*, *Chodl* and *Ptx4* were enriched in Cluster 22, whereas *Cxcl12* and *Kitl* were enriched in Cluster 21. By contrast, *Sp7* and *Bglap* were enriched in distinct osteoblastic clusters, indicating that Clusters 21 and 22 are transcriptionally distinct from osteoblast-lineage populations.

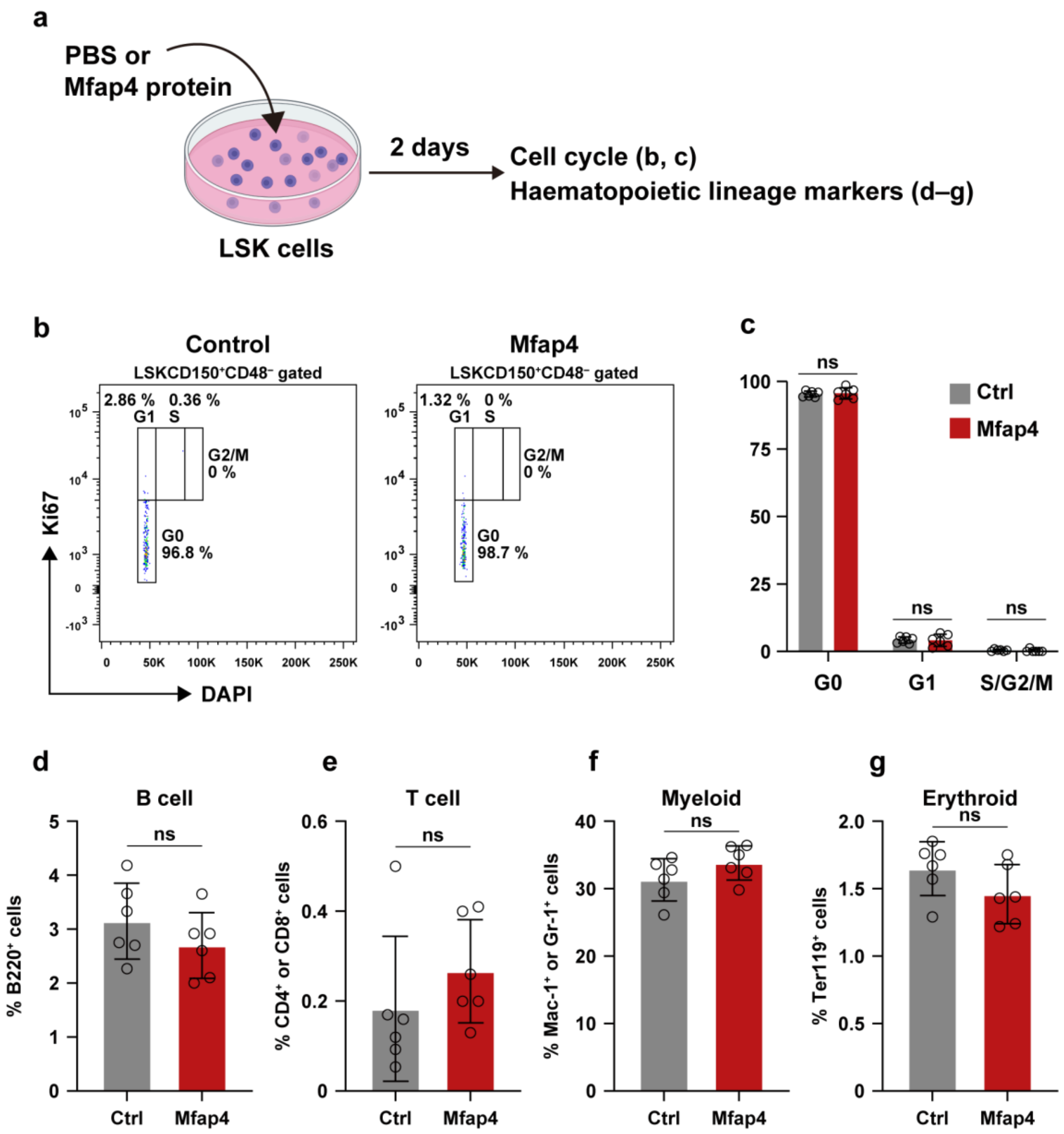


**Extended Data Fig. 13**

**Extended Data Figure 13. Effects of Mfap4 protein on the cell cycle and differentiation of HSCs.**

**(a)** Schematic representation of LSK cell culture in the presence or absence of Mfap4 protein.

**(b)** Representative flow cytometry plots showing cell cycle distribution of cultured HSCs in the presence or absence of Mfap4 (2.5 μg/ml).

**(c)** The percentages of HSCs in G0, G1 and S/G2/M phases.

**(d–g)** The frequencies of mature haematopoietic lineage cells derived from cultured HSCs. **(d)** B cells. **(e)** T cells. **(f)** Myeloid cells. **(g)** Erythroid cells.

Data are presented as mean ± SD (n = 6/group). Statistical differences were determined by an unpaired two-tailed Student's t-test: ns, not significant. Created in BioRender. Arai, F. (2026) https://BioRender.com/xo1818a.
