# Supplementary material for: Endosteal integrin-α8⁺ mesenchymal stem cells maintain haematopoietic stem cell function via extracellular matrix-mediated interactions
Source: Nat Commun. 2026 Jul 6;17:8355. doi: 10.1038/s41467-026-75276-2 (PMC13473570; doi:10.1038/s41467-026-75276-2)
Supplement: Supplementary file 2 [file 41467_2026_75276_MOESM2_ESM.pdf]

nature portfolio

Peer Review File

Endosteal Integrin-α8⁺ Mesenchymal Stem Cells Maintain Haematopoietic Stem Cell Function via Extracellular Matrix-Mediated Interactions

Corresponding Author: Dr Fumio Arai

This file contains all reviewer reports in order by version, followed by all author rebuttals in order by version.

Version 0:

Reviewer comments:

Reviewer #1

(Remarks to the Author)
The study by Yuta R is a remarkable step forward towards the characterization of the endosteal bone marrow (BM) niche for hematopoietic stem cells (HSCs). The authors build on their previous studies to further characterize the subpopulations of mesenchymal stromal cells (MSCs) maintaining HSCs in this niche. Through a screening approach they identify integrin alpha8 (Itga8) as a candidate marker of primitive, MSCs not yet committed towards osteolineage cells, in the endosteal niche. The authors generate a new mouse line allowing conditional ablation and lineage-tracing of Itga8+ MSCs. In combination with immunofluorescence studies, the results unambiguously demonstrate that Itga8 marks endosteal niche cells with seemingly primitive MSC features that allow to maintain HSCs in vivo and ex vivo. Finally, they investigate potential molecules involved in the Itga8+ MSC-HSC interaction and identify microfibrillar-associated protein 4 (Mfap4) produced by Itga8+ MSC and binding to ItgaV and Itgb3 on HSCs as a novel adhesion molecule maintaining HSCs in the endosteal niche. Overall, the manuscript is very clearly written and presented, and easy to follow. Below are relatively minor comments aimed at further improving the presentation of this highly interesting study.

General Comments

Overall

- Carefully proofread the entire manuscript, including figure legends, as some misspellings and formatting issues remain.

- Improve figure labeling for clarity and consistency.

- The submitted version appears to have been generated from a Word document containing previous co-author comment. Approving these and submitting a clean version would be encouraged.

Abstract

“Itga8+ MSCs have higher haematopoietic supportive capacity than other MSC subsets. Transwell assays indicated that the HSC-supporting activity of Itga8+ MSCs is mediated through cell-cell and cell-extracellular matrix ECM interactions. RNA sequencing revealed that Itga8+ MSCs exhibit low expression of haematopoietic niche factors and high expression of cell adhesion molecules and ECM components. Notably, Itga8+ MSCs specifically express Mfap4, a factor which enhances the BM reconstitution capacity of HSCs. These findings suggest that Itga8+ MSCs constitute a novel niche cell population essential for HSC maintenance, providing new insights into the regulation of HSCs.”
Current phrasing appears counter intuitive, I would suggest to rephrase for the reader to catch the essence of the paper. Itga8+ support HSC pool maintenance via direct cell-cell contact and expression of the specific adhesion molecule Mfap4.

The abstract currently lacks one of the key translational aspects of your work – potential implication of Itga8+ MSC potential to optimize in vitro HSC maintenance and enhance in vivo engraftment.

Introduction

46-57 – this section could be refined for clarity, flow, and precision. The current wording is somewhat repetitive, and the transition between ideas could be smoother.

68 – The term 'specifically' may not be entirely accurate, as your data show that Itga8 is also expressed in other MSC subsets, suggesting high enrichment but no exclusivity. More broadly, comparing the transcriptional profile of Itga8⁺ MSCs with other MSC populations previously identified in the endosteal niche (e.g. N-cadherin+ MSCs) would allow to better place this population in the context of the existing knowledge and literature.

70 – For clarity and accuracy, please refine this section. Your data demonstrate that Itga8⁺ MSCs support HSC quiescence and long-term repopulating (LTR) capacity.
71 – The current scope of the study may not fully support the claim that Itga8⁺ MSCs specifically express Mfap4. Consider refining this statement to reflect the strength of the data. Was Mfap4 reduced in mice with deletion of Itga8+ MSCs?

Results

A unique subpopulation expressing Itga8 exists in Alcam+Sca-1– BLCs in the endosteal region of the BM.

A brief introduction to the model used for generating the present data would enhance clarity and provide helpful context.

Representative pictures in Figure 2b are quite convincing, but show ideally be accompanied by quantification of independent samples.

88 – see previous comment on the term “specifically” when referring to Itg8a+ expression within Alcam+Sca1- BLCs

90,91 – The phrasing 'almost absent' is somewhat vague and may benefit from a more quantitative statement. Additionally, stating that Itga8⁺ cells are 'rare in the endosteum' based solely on their presence among Alcam⁺ Sca-1⁻ BLCs expressing osteoblast markers may be an overstatement. Clarifying whether this conclusion applies broadly to the endosteal niche remains needed.

93, 94 – reinforcing the previous comment regarding specificity. Additional markers could enhance niche characterization (including hematopoietic and vascular niche).

Itga8+Alcam+Sca-1– BLCs define a distinct MSC population in the endosteal niche.

The two results section titles are very similar in meaning, potentially leading to redundancy. Consider differentiating them more clearly to emphasize distinct aspects of the findings.

141 – The statement "No Itga8 expression was detected in Sca-1+ MSCs and CD51+CD140a+ MSCs at both protein and mRNA levels" contradicts Extended Data Fig. 3b, c, which actually shows Itga8 surface and mRNA expression within the CD51+CD140a+ fraction as well as in the Alcam-Sca1+ fraction. This discrepancy needs to be addressed to avoid misinterpretation of the data.

144 -146 – The sudden mention of LepRCre/+; Rosa-DTR-tdTomato mice in the latter part of the paragraph disrupts the logical flow, as this model was not introduced earlier. To improve readability, introduce this model before discussing its relevance in comparison to Itga8Cre/+; Rosa-DTR-tdTomato mice. This will ensure that the reader follows the rationale behind the comparison. If you introduce this model, please deepen the results observed in LepR model (Impact of ablation on MSCs subsets, Itg8a+ expression within MSCs susbsets…)

156 – Itg8a+

150-165 –

Itg8+ MSC ablation resulted in decreased proportion of HSC in G0. Is this compensatory HSC proliferation following their partial reduction? Does this happen only in the endosteal niche or throughout the BM?

The transplantation experiment confirms that HSCs from DT-treated mice show reduced engraftment in lethally irradiated recipients, suggesting a defect in LTR capacity. If compatible with the revision timeframe secondary transplantation results would confirm the self-renewal impairment.

Itga8+ MSCs maintain HSCs through cell-cell and ECM interactions.
In zebrafish models, Mfap4 deletion led to reduced Gata2 and Runx1 expression, a decrease in macrophages, and an increase in neutrophils. This suggests that Mfap4 could have a regulatory role in hematopoiesis, potentially balancing lymphoid vs. myeloid differentiation in mammals.
If Mfap4 plays a role in steady-state hematopoiesis, it might compensate for Itga8+ MSC depletion by maintaining HSC survival or differentiation potential, thereby preventing a drop in PB lymphoid populations? Is there compensatory Mfap4 upregulation by other MSCs subsets?
Suggestions for future experiments (outside the scope of this revision) to further investigate the link between Mfap4, Itg8a+ MSCs and HSCs maintenance/ LTR   Mfap4 Knockout or Mfap4-Blocking Experiments   Does Mfap4 inhibition phenocopy

Itga8+ MSC depletion? Measuring integrin activation (e.g., phospho-FAK, phospho-SMAD2/3) in the context of the different coculture settings could add further functional evidence, as alluded to in the discussion.

Discussion

310 – has

The discussion was overall well written, with strong concluding remarks that effectively summarize the key findings and their potential implications. Placing these in the broader state of the art, particularly in context of other MSC populations previously identified in this niche, would add value. What would be the main physiological role of Itga8+ MSCs? To facilitate HSC adhesion in a specialized microenvironment where other niche cells (such as CXCL12- and SCF-producing MSCs) would promote HSC quiescence and maintenance? And/or would Itga8+ MSCs be a “reservoir” population of MSCs that contribute to skeletal repair or regeneration (but not to homeostatic skeletal turnover)?

Reviewer #2

(Remarks to the Author)
In this study, Yuta et al. report a previously uncharacterized BM MSC cell type identified by ITGA8 expression and evaluate its role in HSC function through mediating ECM-HSC interaction. While ITGA8 is highly expressed in renal tissue and has previously been shown to play a role in vascular smooth muscle cell ECM interaction and mediating their functon(Nat Cardiovasc Res 2023), this is the first alpha 8 integrin-DTR tDT reporter mouse that enables visualization and specific targeting of ITGA8+ BM stromal cells. Here, the researchers found that BM lining cells, identified by Alcam, express high levels of ITGA8 compared to other stromal populations. These cells, like MSCs, exhibit robust trilineage differentiation (osteogenic, adipocyte, and chondrocyte) and distinct molecular features. Functionally, the authors claim that these cells support normal hematopoiesis, both at steady-state and in hematopoietic recovery. Mechanistically, the authors claim that the ECM molecule, Mfap4, mediates ITGA8+ MSCs' role in supporting HSC function via cell-cell interaction.

While the mouse model is a useful resource for the scientific community, overall, there are several general concerns about the study. Additional experiments would strengthen this manuscript:
1. All their in vivo functional studies show differences in CD45+ reconstitution with comparable lineage or progenitor chimerism—this does not support impaired HSC multilineage reconstitution. Additionally, there are no secondary transplant studies performed to evaluate HSC self-renewal properties. The sample size is insufficient.
2. Do Itga8-depleted recipient mice support hematopoietic reconstitution?
3. In the co-culture experiments with subsequent transplants, what cells are being transplanted? What percentage are HSCs?
4. Mechanistic studies do not support the role of the Itga8-Mfap4 axis in HSC function. Rescue experiments with Mfap4 are difficult to interpret since these were not performed either in the context of Itga8+ depletion or using their transwell system (which disrupts HSPC-Itga8 cell interaction). Depletion of Mfap4 would also strengthen these studies.

Some additional, more specific points to be addressed are listed below:
Major Points
a. Figure 3c: It is unclear if there is costaining between TdTomato+ cells and ITGA8.
b. Figure 3 (F-G) and Extended Figure 4: The data does not support the claim "The depletion of Itga8+ MSCs leads to a decrease in HSC function."
• Missing baseline CBC and BM HSPC characterization in ITGA8-depleted mice.
• Are there differences in HSC apoptosis?
• Are there any hematopoietic recovery defects in Itga8-depleted mice following 5-FU challenge?
• There are no differences in HSC function following competitive transplantation. The sample size is insufficient. Overall, the current experiment does not evaluate Itga8+ MSCs' role in supporting regenerating HSCs in stress hematopoiesis. I would consider assessing reconstitution defects in WT HSCs transplanted in Itga8-depleted recipient mice to better demonstrate this role.
c. Figures 4 and 5: The HSC-ITGA8+ MSC coculture experiments, although well-conceived, do not demonstrate their claim of supporting HSC long-term reconstitution properties. Specifically, it is unclear how their observations in overall CD45+ chimerism align with no differences in individual lineage chimerism. Additionally, there are paradoxical findings, such as better myeloid reconstitution with ITGA8- cells. Considering:
• What is the percentage of HSCs recovered following 48 hours post-coculture in Figure 4 and 5?
• What is the HSPC reconstitution?
• The sample size is insufficient.
d. Figure 6, Panel F: It is unclear from IF if ITGA8+ cells express Mfap4.
e. Figure 7: Similar comments to Figures 4 and 5. The sample size is insufficient. Data does not support the claims:
• "Mfap4 protein suppressed the proliferation of HSCs" (lines 249-250). While there is evidence for decreased expansion of hematopoietic precursors and mature cells with HSC expansion, without cell cycle analysis and thorough phenotypic characterization of these ex vivo cultured HSPC cells, it is unclear if Mfap4 is inducing quiescence or a differentiation block.
• "examined the effect of Mfap4 on the self-renewal of HSCs" (line 252). There is no evidence for improved multilineage reconstitution (Extended Figure 7), let alone self-renewal capacity (SRC), which cannot be evaluated with a primary transplant.
Minor Points
a. Figure 1b: Label panel "ITGA8 RNA".

b. Extended Figure 5b: Provide quantification.
c. Extended Figure 3b: Revise label—it's the protein, not the antibody, being measured.
d. There is inconsistent formatting across figure legends (e.g., some use full sentences while others use fragments).
e. Some panels aren't fully explained (especially in supplementary figures). For example, Extended Figure 5c-e: it is unclear which BMT is being referred to here.
f. Some abbreviations are used before being defined.
g. Panel labels (A, B, C, etc.) aren't always clearly linked to their descriptions.
h. Show representative flow plots, especially for ex vivo transwell coculture studies.

Version 1:

Reviewer comments:

Reviewer #1

(Remarks to the Author)
The authors have sufficiently addressed all of the comments.
The revised manuscript is much more accurate, balanced and supported.
I have no further comments on this highly interesting and important study.

Reviewer #2

(Remarks to the Author)
The authors have responded to all the concerns.

Open Access This Peer Review File is licensed under a Creative Commons Attribution 4.0 International License, which permits use, sharing, adaptation, distribution and reproduction in any medium or format, as long as you give appropriate credit to the original author(s) and the source, provide a link to the Creative Commons license, and indicate if changes were made.
In cases where reviewers are anonymous, credit should be given to 'Anonymous Referee' and the source.
The images or other third party material in this Peer Review File are included in the article's Creative Commons license, unless indicated otherwise in a credit line to the material. If material is not included in the article's Creative Commons license and your intended use is not permitted by statutory regulation or exceeds the permitted use, you will need to obtain permission directly from the copyright holder.
To view a copy of this license, visit https://creativecommons.org/licenses/by/4.0/

Reviewer #1:

The study by Yuta R is a remarkable step forward towards the characterization of the endosteal bone marrow (BM) niche for hematopoietic stem cells (HSCs). The authors build on their previous studies to further characterize the subpopulations of mesenchymal stromal cells (MSCs) maintaining HSCs in this niche. Through a screening approach they identify integrin alpha8 (Itga8) as a candidate marker of primitive, MSCs not yet committed towards osteolineage cells, in the endosteal niche. The authors generate a new mouse line allowing conditional ablation and lineage-tracing of Itga8+ MSCs. In combination with immunofluorescence studies, the results unambiguously demonstrate that Itga8 marks endosteal niche cells with seemingly primitive MSC features that allow to maintain HSCs in vivo and ex vivo. Finally, they investigate potential molecules involved in the Itga8+ MSC-HSC interaction and identify microfibrillar-associated protein 4 (Mfap4) produced by Itga8+ MSC and binding to ItgaV and Itgb3 on HSCs as a novel adhesion molecule maintaining HSCs in the endosteal niche. Overall, the manuscript is very clearly written and presented, and easy to follow. Below are relatively minor comments intended to further improve the presentation of this highly interesting study.

[Response]

We appreciate the reviewer's generous and encouraging overall assessment of our work. We are pleased that the reviewer recognizes the significance of our study in advancing the characterization of the endosteal bone marrow niche and in identifying Itga8+ MSCs as a primitive endosteal MSC population that supports HSC maintenance. We also appreciate the reviewer's positive evaluation of the clarity and presentation of the manuscript. We have carefully addressed the comments below and revised the manuscript accordingly.

Specific comments

1. "Itga8+ MSCs have higher haematopoietic supportive capacity than other MSC subsets. Transwell assays indicated that the HSC-supporting activity of Itga8+ MSCs is mediated through cell-cell and cell-extracellular matrix ECM interactions. RNA sequencing revealed that Itga8+ MSCs exhibit low expression of haematopoietic niche factors and high expression of cell adhesion molecules and ECM components. Notably, Itga8+ MSCs specifically express Mfap4, a factor which enhances the BM reconstitution capacity of HSCs. These findings suggest that Itga8+ MSCs constitute a novel niche cell population essential for HSC maintenance, providing new insights into the regulation of HSCs."

The abstract currently lacks one of the key translational aspects of your work – potential implication of Itga8+ MSC potential to optimize in vitro HSC maintenance and enhance in vivo engraftment.

[Response]

We appreciate the reviewer's comment. We agree that the translational implication of our findings should be stated more clearly in the Abstract. In response, we revised the Abstract to indicate that Itga8$^+$ MSCs support HSC maintenance through direct cell-cell and cell-ECM interactions, and that these findings may provide a basis for improving in vitro HSC maintenance and enhancing engraftment after transplantation.

2. Line 46-57 – this section could be refined for clarity, flow, and precision. The current wording is somewhat repetitive, and the transition between ideas could be smoother.

[Response]

Thank you for your valuable comment. We revised this section to eliminate repetition, strengthen the connections between concepts, and improve the overall clarity and accuracy.

3. Line 68 – The term 'specifically' may not be entirely accurate, as your data show that Itga8 is also expressed in other MSC subsets, suggesting high enrichment but no exclusivity. More broadly, comparing the transcriptional profile of Itga8$^+$ MSCs with other MSC populations previously identified in the endosteal niche (e.g. N-cadherin+ MSCs) would allow to better place this population in the context of the existing knowledge and literature.

[Response]

We thank the reviewer for this valuable suggestion. Since Itga8 expression is not restricted to Alcam$^+$Sca-1$^-$ BLCs, we changed "specifically" to "characteristically" throughout. In addition, to better place Itga8$^+$ MSCs in the context of previously described endosteal MSC populations, we compared their transcriptional profile with that of N-cadherin$^+$ MSCs using published RNA-seq data. These analyses showed that Itga8$^+$ MSCs are transcriptionally distinct from N-cadherin$^+$ MSCs and exhibit different expression patterns of niche-associated genes. These results have now been included as the revised Extended Data Figure 3. (Line 136~)

4. Line 70 – For clarity and accuracy, please refine this section. Your data demonstrate that Itga8$^+$ MSCs support HSC quiescence and long-term repopulating (LTR) capacity.

[Response]

Thank you for your valuable comment. We changed the sentence to "Itga8$^+$ MSCs play a crucial role in supporting HSC quiescence and maintaining long-term repopulating (LTR) capacity." (Line 63~)

5. Line 71 – The current scope of the study may not fully support the claim that Itga8⁺ MSCs specifically express Mfap4. Consider refining this statement to reflect the strength of the data. Was Mfap4 reduced in mice with deletion of Itga8⁺ MSCs?

[Response]

We thank the reviewer for this helpful comment. We have removed this sentence and revised the text to more cautiously describe Mfap4 as a candidate factor enriched in $Itga8^{+}$ MSCs, as suggested. We did not directly examine whether Mfap4 expression was reduced after deletion of $Itga8^{+}$ MSCs, and so we have avoided making claims beyond the data.

6. A unique subpopulation expressing Itga8 exists in Alcam+Sca-1− BLCs in the endosteal region of the BM. A brief introduction to the model used for generating the present data would enhance clarity and provide helpful context.

[Response]

We thank the reviewer for this helpful comment. We added the following text as an addendum to the main text: "After single-cell RNA amplification, multi-gene expression profiling was conducted using a high-density microfluidic device to characterise the subpopulations in detail." In addition, to provide clearer context for the model and dataset used to identify the $Itga8^{+}$ subpopulation, we now include the corresponding single-cell RNA-seq (scRNA-seq) data as the revised Figure 6 and Extended Data Figure 12. These figures illustrate the presence of the $Itga8^{+}$ subpopulation within $Alcam^{+}Sca\text{-}1^{-}$ BLCs in the endosteal BM region. (Line 76~)

7. Representative pictures in Figure 2b are quite convincing, but show ideally be accompanied by quantification of independent samples.

[Response]

We appreciate the reviewer's suggestion. In response, we quantified the staining across independent samples and added the results to Figure 2b.

8. Line 88 – see previous comment on the term "specifically" when referring to $Itg8a^{+}$ expression within $Alcam^{+}Sca1^{-}$ BLCs

[Response]

We appreciate the reviewer's comment. Consistent with the revision made in response to the previous comment, we changed the term "specifically" to "characteristically."

9. Line 90,91 – The phrasing 'almost absent' is somewhat vague and may benefit from a more quantitative statement. Additionally, stating that Itga8^{+} cells are 'rare in the endosteum' based solely on their presence among Alcam^{+} Sca-1^{-} BLCs expressing osteoblast markers may be an overstatement. Clarifying whether this conclusion applies broadly to the endosteal niche remains needed.

[Response]

We appreciate the reviewer's comment. We revised the text to avoid overstatement and to provide a more accurate description. Specifically, Itga8^{+} cells were described as being rarely detected within the osteoblast-marker-positive fraction of Alcam^{+}Sca-1^{-} BLCs. We also agreed that the statement "Itga8^{+} cells highlight their rare presence in the endosteum" was too strong and therefore removed it. (Line 38)

10. Line 93, 94 – reinforcing the previous comment regarding specificity. Additional markers could enhance niche characterization (including hematopoietic and vascular niche). Itga8+Alcam+Sca-1− BLCs define a distinct MSC population in the endosteal niche. The two results section titles are very similar in meaning, potentially leading to redundancy. Consider differentiating them more clearly to emphasize distinct aspects of the findings.

[Response]

We appreciate the reviewer's comment. In line with the previous comments regarding specificity, we revised the relevant text to avoid overstatement. To further characterize the niche environment, we separated the CD45^{+} hematopoietic cell and Ter119^{+} erythrocyte data from Figure 1e and presented them in Figure 1f. In addition, we analyzed Itga8 expression in CD31^{+} endothelial cells and CD51^{+}CD140a^{+} MSCs, which are components of the vascular niche, and included these results in Figure 1g. We also revised the two Results section titles to distinguish more clearly between identification of the Itga8^{+} subpopulation and characterization of its MSC properties. The revised titles are "Identification of an Itga8^{+} subpopulation within Alcam^{+}Sca-1^{-} BLCs in the endosteal BM" and "Itga8^{+}Alcam^{+}Sca-1^{-} BLCs exhibit distinct MSC properties." (Line 72 and 108)

11. Line 141 – The statement "No Itga8 expression was detected in Sca-1^{+} MSCs and CD51^{+}CD140a^{+} MSCs at both protein and mRNA levels" contradicts Extended Data Fig. 3b, c (In the revised version, these are Fig. 4c, d), which actually shows Itga8 surface and mRNA expression within the CD51^{+}CD140a^{+} fraction as well as in the Alcam^{-}Sca1^{+} fraction. This discrepancy needs to be addressed to avoid misinterpretation of the data.

[Response]

We appreciate the reviewer's comment. As pointed out, the original statement was inconsistent with the revised Extended Data Fig. 4c, d. We therefore revised the text to state that Itga8 expression at both the protein and mRNA levels was substantially lower in Sca-1^+ MSCs and $CD51^+CD140a^+$ MSCs than in $Itga8^+$ MSCs. (Line 155~)

12. Line 144 -146 – The sudden mention of $LepR^{Cre/+}$; Rosa-DTR-tdTomato mice in the latter part of the paragraph disrupts the logical flow, as this model was not introduced earlier. To improve readability, introduce this model before discussing its relevance in comparison to $Itga8^{Cre/+}$; Rosa-DTR-tdTomato mice. This will ensure that the reader follows the rationale behind the comparison. If you introduce this model, please deepen the results observed in LepR model (Impact of ablation on MSCs subsets, Itg8a+ expression within MSCs susbsets…)

[Response]

We appreciate the reviewer's comment. To improve the logical flow, we revised this section to introduce the $LepR^{Cre/+}$; Rosa-DTR-tdTomato model before comparing it with the $Itga8^{Cre/+}$; Rosa-DTR-tdTomato model. We originally introduced the LepR model to genetically label $LepR^+$ MSCs and examine their potential relationship to $Itga8^+$ MSCs, including whether $LepR^+$ MSCs give rise to the $Itga8^+$ MSC population. In response to the reviewer's suggestion, we further analyzed the effects of DT administration in $LepR^{Cre/+}$; Rosa-DTR-tdTomato mice on central BM-MSCs, BLCs, and the BM microenvironment, and these results are now presented in Extended Data Fig. 5. These analyses showed that DT administration reduced $tdTomato^+$ LepR-lineage cells, whereas the numbers of $Itga8^+$ BLCs and Sca-1^+ cells were not significantly changed, suggesting that ablation of $LepR^+$ MSCs predominantly affects the vascular niche rather than the endosteal niche. In addition, the numbers of HSCs, CMPs, and GMPs increased after DT administration, suggesting reduced quiescence of HSCs and progenitor cells following disruption of the perivascular niche. (Line 176~)

13. Line 156 – Itg8a+

[Response]

We appreciate the reviewer's comment. We corrected "$Itg8a^+$" to "$Itga8^+$" in the revised manuscript.

14. Line 150-165 – Itg8+ MSC ablation resulted in decreased proportion of HSC in G0. Is this compensatory HSC proliferation following their partial reduction? Does this happen only in the endosteal niche or throughout the BM?

[Response]

We thank the reviewer for this insightful comment. In the original manuscript, the data were derived from HSCs isolated from whole BM. To clarify this issue, we conducted additional analyses of HSC cell-cycle status in both the central and periendosteal BM and included these results in the revised Figure 3h and i. Our findings show that the proportion of quiescent (G0) HSCs was significantly reduced in the periendosteal BM following DT administration, whereas it remained unchanged in the central BM. These results indicate that the loss of HSC quiescence is spatially confined to the periendosteal niche and support the conclusion that Itga8^{+} MSCs are required to maintain HSC quiescence in this region. (Line 202~)

15. The transplantation experiment confirms that HSCs from DT-treated mice show reduced engraftment in lethally irradiated recipients, suggesting a defect in LTR capacity. If compatible with the revision timeframe secondary transplantation results would confirm the self-renewal impairment.

[Response]

We thank the reviewer for this valuable suggestion. We agree that secondary transplantation would provide important additional evidence regarding self-renewal capacity. However, because establishment and preparation of the required mouse cohorts took longer than anticipated, we were unable to complete the secondary transplantation experiments for the current revision. We therefore limited our conclusion to reduced long-term repopulating capacity based on the primary transplantation results and refrained from making a definitive statement regarding impaired self-renewal.

16. Itga8+ MSCs maintain HSCs through cell-cell and ECM interactions. In zebrafish models, Mfap4 deletion led to reduced Gata2 and Runx1 expression, a decrease in macrophages, and an increase in neutrophils. This suggests that Mfap4 could have a regulatory role in hematopoiesis, potentially balancing lymphoid vs. myeloid differentiation in mammals. If Mfap4 plays a role in steady-state hematopoiesis, it might compensate for Itga8+ MSC depletion by maintaining HSC survival or differentiation potential, thereby preventing a drop in PB lymphoid populations? Is there compensatory Mfap4 upregulation by other MSCs subsets?

[Response]

We thank the reviewer for this insightful comment. Depletion of Itga8^{+} MSCs, which highly express Mfap4, led to a significant reduction in common lymphoid progenitors (CLPs) in the BM (Fig. 3g). This finding suggests that factors produced by Itga8^{+} MSCs, including Mfap4, may contribute to lymphoid-supportive functions within the BM niche. However, our current data do not permit us to determine the specific role of Mfap4 in this phenotype. The lack of a clear reduction in peripheral

blood lymphoid populations may be due to the restricted anatomical scope of Itga8$^+$ MSC depletion and the large systemic lymphocyte pool. We also attempted to assess whether other MSC subsets upregulate Mfap4 expression following Itga8$^+$ MSC depletion, but we were unable to isolate sufficient numbers of CD51$^+$CD140a$^+$ MSCs, Sca-1$^+$ MSCs, and Itga8$^-$ BLCs from the tibia for reliable RNA analysis. Therefore, we have refrained from making a definitive statement regarding compensatory Mfap4 upregulation in other MSC subsets.

(Line 292~)

17. Suggestions for future experiments (outside the scope of this revision) to further investigate the link between Mfap4, Itg8a$^+$ MSCs and HSCs maintenance/ LTR ◊ Mfap4 Knockout or Mfap4-Blocking Experiments ◊ Does Mfap4 inhibition phenocopy Itga8$^+$ MSC depletion? Measuring integrin activation (e.g., phospho-FAK, phospho-SMAD2/3) in the context of the different coculture settings could add further functional evidence, as alluded to in the discussion.

[Response]

We thank the reviewer for these valuable suggestions. We agree that further investigation of Mfap4 and downstream integrin-associated signaling would provide important mechanistic insight into the link between Itga8$^+$ MSCs and HSC maintenance/LTR capacity. In response to this comment, we added a statement to the Discussion noting that further studies on Mfap4 and downstream integrin-associated signaling may clarify how Itga8$^+$ MSCs support HSC maintenance and long-term repopulating capacity.

(Line 461~)

18. Line 310 – has

[Response]

We appreciate the reviewer’s comment. We corrected this typographical error in the revised manuscript.

19. The discussion was overall well written, with strong concluding remarks that effectively summarize the key findings and their potential implications. Placing these in the broader state of the art, particularly in context of other MSC populations previously identified in this niche, would add value. What would be the main physiological role of Itga8$^+$ MSCs? To facilitate HSC adhesion in a specialized microenvironment where other niche cells (such as CXCL12- and SCF-producing MSCs) would promote HSC quiescence and maintenance? And/or would Itga8$^+$ MSCs be a “reservoir” population of MSCs that contribute to skeletal repair or regeneration (but not to homeostatic skeletal turnover)?

[Response]

We appreciate the reviewer's insightful comment. In response, we expanded the Discussion to more clearly position Itga8$^+$ MSCs within the context of previously identified MSC populations in the bone marrow niche. Specifically, we now note that, in contrast to conventional cytokine-rich MSC populations, Itga8$^+$ MSCs secrete abundant extracellular matrix-related molecules and may provide a structural scaffold around the endosteum, thereby contributing to a niche environment distinct from the vascular niche. Additionally, we further noted that rare Itga8$^+$ MSCs may function as a reservoir-like population within the MSC compartment, potentially giving rise to other MSC subsets, such as Sca-1$^+$ MSCs, CD51$^+$CD140a$^+$ MSCs, and LepR$^+$ MSCs, and thus may contribute to tissue maintenance or repair under specific conditions.

(Line 437~, and Line473~)

Reviewer #2:

In this study, Yuta et al. report a previously uncharacterized BM MSC cell type identified by ITGA8 expression and evaluate its role in HSC function through mediating ECM-HSC interaction. While ITGA8 is highly expressed in renal tissue and has previously been shown to play a role in vascular smooth muscle cell ECM interaction and mediating their functon (Nat Cardiovasc Res 2023), this is the first alpha 8 integrin-DTR tDT reporter mouse that enables visualization and specific targeting of ITGA8+ BM stromal cells. Here, the researchers found that BM lining cells, identified by Alcam, express high levels of ITGA8 compared to other stromal populations. These cells, like MSCs, exhibit robust trilineage differentiation (osteogenic, adipocyte, and chondrocyte) and distinct molecular features. Functionally, the authors claim that these cells support normal hematopoiesis, both at steady-state and in hematopoietic recovery. Mechanistically, the authors claim that the ECM molecule, Mfap4, mediates ITGA8+ MSCs' role in supporting HSC function via cell-cell interaction.

[Response]

We appreciate the reviewer's thoughtful overall assessment of our study. We are encouraged that the reviewer recognized the novelty of our $Itga8^{Cre/+}$; Rosa-DTR-tdTomato mouse model and the significance of identifying $Itga8^{+}$ MSCs as a previously uncharacterized bone marrow stromal population with distinct molecular and functional properties. We also appreciate the reviewer's summary of our findings regarding the role of these cells in supporting HSC function through ECM-related interactions. In response to the comments below, we have carefully revised the manuscript, particularly where the wording of our conclusions required greater precision, including the description of Mfap4 and the functional interpretation of $Itga8^{+}$ MSCs in hematopoiesis.

1. All their in vivo functional studies show differences in CD45+ reconstitution with comparable lineage or progenitor chimerism—this does not support impaired HSC multilineage reconstitution. Additionally, there are no secondary transplant studies performed to evaluate HSC self-renewal properties. The sample size is insufficient.

[Response]

We appreciate the reviewer's important comment. We agree that CD45 chimerism alone is insufficient to evaluate multilineage reconstitution capacity. In response, we analyzed donor-derived myeloid, B-cell, and T-cell reconstitution in the relevant transplantation experiments and added these data to the revised manuscript (Extended Data Figs. 7b–d, Fig. 7g–I, and Extended Data Figs.10c–e). These analyses showed that donor-derived lineage output was broadly comparable between groups, indicating that our data do not support a major defect in multilineage differentiation. We also agree that secondary transplantation would be required to more directly assess self-renewal capacity.

Therefore, in the revised manuscript, we have limited our conclusion to reduced long-term repopulating capacity and avoided making a definitive claim regarding impaired self-renewal. We also acknowledge the reviewer's concern regarding sample size and have accordingly taken care to avoid overinterpretation of the transplantation results.

2. Do Itga8-depleted recipient mice support hematopoietic reconstitution?

[Response]

We appreciate the reviewer's important suggestion. We attempted reverse transplantation experiments using Itga8$^{Cre/+}$; Rosa-DTR-tdTomato mice as recipients. However, the results were difficult to interpret in this model. Integrin α8 is highly expressed in the kidney, and in Itga8$^{Cre/+}$; Rosa-DTR-tdTomato mice, strong tdTomato expression was observed throughout the kidney (Fig. R1), indicating widespread DTR expression in renal Itga8^{+} cells. As a result, DT administration caused marked renal injury, which itself is expected to affect hematopoiesis and BM function. In particular, renal damage is associated with reduced erythropoietin (EPO) production, which can directly influence hematopoietic recovery. In addition, recipient mice showed severe deterioration in general condition after irradiation. Therefore, the recipient transplantation experiments in this system did not allow a clear assessment of BM niche function specifically attributable to Itga8+ MSC depletion.

Fig. R1

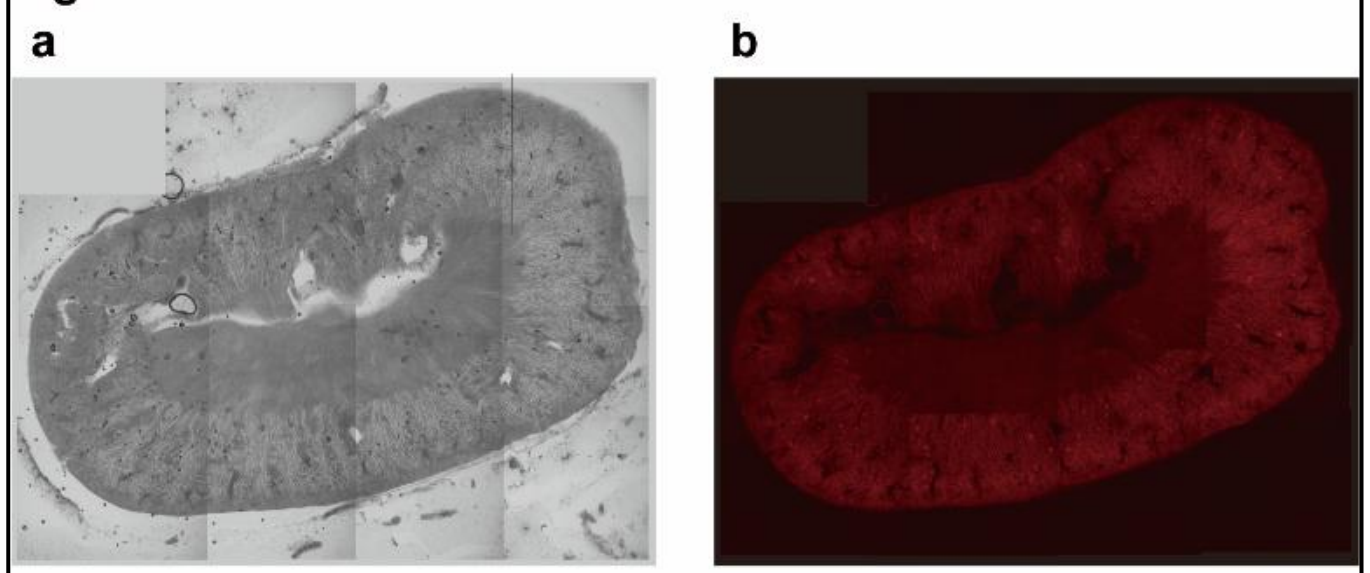

Figure. R1. Strong tdTomato expression throughout the kidney in Itga8Cre/+; Rosa-DTR-tdTomato mice.
(a) Brightfield image of the kidney.
(b) tdTomato fluorescence image of the same kidney, showing widespread labeling of renal Itga8^{+} cells.

3. In the co-culture experiments with subsequent transplants, what cells are being transplanted? What percentage are HSCs?

[Response]

We appreciate the reviewer's comment. After coculture, we isolated the lineage-negative (Lin^{-}) cell fraction and transplanted 200 Lin^{-} cells into recipient mice. The proportion of HSCs within the Lin^{-} fraction after coculture is shown in Fig. 4d.

4. Mechanistic studies do not support the role of the Itga8-Mfap4 axis in HSC function. Rescue experiments with Mfap4 are difficult to interpret since these were not performed either in the context of Itga8+ depletion or using their transwell system (which disrupts HSPC-Itga8 cell interaction). Depletion of Mfap4 would also strengthen these studies.

[Response]

We appreciate the reviewer's important comment. We agree that our current data do not fully establish a definitive mechanistic role for Mfap4 in the regulation of HSCs by Itga8$^+$ MSCs. We would like to clarify that the Mfap4 supplementation experiments were performed without coculture with Itga8$^+$ MSCs and therefore in the absence of direct cell-cell contact (Fig. 7), this is conceptually analogous to the Transwell® condition. At the same time, we recognize that these experiments do not directly test rescue in the context of Itga8$^+$ MSC depletion. We also agree that loss-of-function studies targeting Mfap4 would provide stronger mechanistic evidence. Therefore, in the revised manuscript, we have tempered our interpretation and describe Mfap4 more cautiously as a candidate ECM-related mediator produced by Itga8$^+$ MSCs, rather than as a definitively validated mechanism. (Line 295~, Line 306~)

Major points

5. Figure 3c: It is unclear if there is co-staining between TdTomato+ cells and ITGA8.

[Response]

We appreciate the reviewer's comment. To clarify the spatial relationship between Itga8 and tdTomato expression in the endosteal niche, we added high-magnification images of immunohistochemical co-staining to the revised Extended Data Fig. 4b. These images allow clearer visualization of the overlap between Itga8 and tdTomato signals in the endosteal BM region.

6. Figure 3 (F-G) and Extended Figure 4: The data does not support the claim "The depletion of Itga8+ MSCs leads to a decrease in HSC function."

[Response]

We appreciate the reviewer's comment. As pointed out, the reduction in HSC number in the BM of Itga8$^+$ MSC-depleted mice does not by itself demonstrate impaired HSC function. However, because Figure 3 also includes transplantation data showing reduced engraftment capacity of LSK cells from Itga8$^+$ MSC-depleted mice, we revised the Figure 3 title from "The depletion of Itga8$^+$ MSCs leads to a decrease in HSC function" to "The depletion of Itga8$^+$ MSCs leads to a reduction in functional HSCs." (Line 993)

7. Missing baseline CBC and BM HSPC characterization in ITGA8-depleted mice.

[Response]

We appreciate the reviewer's comment. In response, we added baseline complete blood count (CBC) data and further characterization of BM hematopoietic stem and progenitor cells (HSPCs) after depletion of Itga8$^+$ MSCs. CBC analysis showed no significant differences after local depletion of Itga8$^+$ MSCs, and these data are now included in Extended Data Fig. 6. We also present the changes in BM HSPC populations, including HSCs, CMPs, GMPs, MEPs, and CLPs, in Fig. 3 and Extended Data Fig. 6. These analyses showed that HSCs and CLPs were reduced, whereas other progenitor populations and mature PB cell counts were not significantly changed.

8. Are there differences in HSC apoptosis?

[Response]

We appreciate the reviewer's comment. We confirmed that depletion of Itga8$^+$ MSCs did not induce apoptosis in HSCs. The corresponding flow cytometry plot has been added to Extended Data Fig. 6l.

9. Are there any hematopoietic recovery defects in Itga8-depleted mice following 5-FU challenge?

[Response]

We appreciate the reviewer's comment. To address this point, we administered 5-FU to Itga8$^{Cre/+}$; Rosa-DTR-tdTomato mice treated with PBS or DT and assessed hematopoietic recovery 10 days later (Extended Data Fig. 8a–j). Hematopoietic recovery after 5-FU administration was significantly delayed in mice depleted of Itga8+ MSCs. These results suggest that Itga8$^+$ MSCs are required to maintain an adequate HSC pool under hematopoietic stress.

10. There are no differences in HSC function following competitive transplantation. The sample size is insufficient. Overall, the current experiment does not evaluate Itga8+ MSCs' role in supporting regenerating HSCs in stress hematopoiesis. I would consider assessing reconstitution defects in WT HSCs transplanted in Itga8-depleted recipient mice to better demonstrate this role.

[Response]

We appreciate the reviewer's important comment. We agree that the transplantation data should be interpreted cautiously, particularly given the limited sample size, and we have therefore avoided overstatement in the revised manuscript. At the same time, our primary transplantation data support reduced long-term repopulating capacity of HSCs following depletion of Itga8$^+$ MSCs. Because the

BM niche is composed of multiple stromal populations, depletion of a rare stromal subset would not necessarily be expected to abolish all niche support immediately. To complement these data, we evaluated hematopoietic recovery after 5-FU administration and found that recovery was delayed in Itga8+ MSC-depleted mice (Extended Data Fig. 8), supporting a role for Itga8^{+} MSCs in hematopoietic regeneration under stress. We also appreciate the reviewer's suggestion to assess reconstitution of WT HSCs in Itga8+ MSC-depleted recipient mice. However, as described above, this recipient model is difficult to interpret because Itga8 is strongly expressed in the kidney, and DT administration in Itga8$^{Cre/+}$; Rosa-DTR-tdTomato mice causes marked renal injury, which would substantially confound hematopoietic reconstitution analyses.

11. Figure 4 and 5: What is the percentage of HSCs recovered following 48 hours post-coculture in Figure 4 and 5?

[Response]

We appreciate the reviewer's comment. The percentage of HSCs recovered 48 hours after coculture is shown in Fig. 4d and Fig. 5d.

12. Figure 4 and 5: What is the HSPC reconstitution?

[Response]

We appreciate the reviewer's comment. In this study, reconstitution in the BM HSC fraction was assessed by the proportion of donor-derived CD48^{-}CD150^{+} cells within the LSK fraction in the BM (Figs. 4i and 5f).

13. Figure 4 and 5: The sample size is insufficient.

[Response]

We appreciate the reviewer's comment. We acknowledge that the sample size is limited and have therefore avoided overstatement in the revised manuscript. Our conclusions have been restricted to what is supported by the statistically significant differences observed in these experiments.

14. Figure 6, Panel F: It is unclear from IF if ITGA8+ cells express Mfap4.

[Response]

We appreciate the reviewer's comment. We agree that the immunofluorescence image in Fig. 6f does not clearly demonstrate Mfap4 expression in Itga8$^+$ cells. To address this point, we added single-cell RNA-seq analysis of Alcam$^+$ BLCs as the revised Figure 6g–j and the revised Extended Data Figure 12. In this analysis, Alcam+ BLCs were divided into 23 clusters, and Itga8 expression was enriched in Cluster 22. Mfap4 expression was also highly enriched in the same cluster, indicating that Itga8$^+$ MSCs represent a major Mfap4-producing population within the endosteal BLC compartment. In addition, Postn and Mfap5 were enriched in the same cluster, whereas LepR, Cxcl12, and Kitl were enriched in a distinct cluster. These findings support the interpretation that Mfap4 is preferentially associated with the Itga8$^+$ MSC population. (Line 302~310)

15. Figure 7: Similar comments to Figures 4 and 5. The sample size is insufficient. Data does not support the claims: "Mfap4 protein suppressed the proliferation of HSCs" (lines 249-250). While there is evidence for decreased expansion of hematopoietic precursors and mature cells with HSC expansion, without cell cycle analysis and thorough phenotypic characterization of these ex vivo cultured HSPC cells, it is unclear if Mfap4 is inducing quiescence or a differentiation block.

[Response]

We appreciate the reviewer's important comment. In response, we evaluated the effects of Mfap4 protein on the cell-cycle status and differentiation of cultured hematopoietic cells. LSK cells were cultured in MyeloCult medium with or without Mfap4, and cell-cycle distribution and hematopoietic differentiation markers were analyzed. Under these conditions, most cells remained in G0 phase, and no significant differences in cell-cycle distribution were observed between the control and Mfap4-treated groups (Extended Data Figure 12a, b). Likewise, analysis of hematopoietic differentiation markers did not reveal significant differences between the two groups (Extended Data Figure 12c–f). As shown in the original submission, Mfap4 supplementation suppressed the expansion of CD45$^+$ hematopoietic cells in a dose-dependent manner while maintaining the proportion of HSCs. This finding suggests that Mfap4 may help preserve the immature HSPC compartment rather than simply promoting overall hematopoietic expansion. We therefore revised the manuscript to avoid overinterpreting the Mfap4 data and to more cautiously describe Mfap4 as a candidate ECM-related factor that may contribute to the maintenance of HSC function. (Line 295~, Line 306~)

16. Figure 7: "examined the effect of Mfap4 on the self-renewal of HSCs" (line 252). There is no evidence for improved multilineage reconstitution (Extended Figure 7), let alone self-renewal capacity (SRC), which cannot be evaluated with a primary transplant.

[Response]

We appreciate the reviewer’s comment. We agree that primary transplantation does not allow direct assessment of HSC self-renewal capacity. Accordingly, we revised the text to avoid overinterpreting the transplantation results as evidence of self-renewal. Instead, we now describe Mfap4 more cautiously as a candidate ECM-related factor that contributes to the maintenance of HSC function during ex vivo culture (Line 376-377). In addition, to further characterize the effect of Mfap4, we included cell-cycle analysis of cultured HSCs (Extended Fig. 13), which showed that Mfap4 treatment influences the cell-cycle status of HSCs (Line 364-369). We have revised the relevant text accordingly throughout the manuscript.

Minor points

17. Figure 1b: Label panel "ITGA8 RNA".

[Response]

We appreciate the reviewer’s comment. We revised the panel label in Fig. 1b to indicate Itga8 mRNA.

18. Extended Figure 5b: Provide quantification.

[Response]

We appreciate the reviewer’s comment. In the original submission, the representative flow cytometry plots included the percentages of the relevant populations within the panels. In the revised manuscript, these data are shown in new Extended Data Fig. 9. The quantitative results from the Transwell culture experiments are presented in Fig. 5b-d.

19. Extended Figure 3b: Revise label—it's the protein, not the antibody, being measured.

[Response]

We appreciate the reviewer’s comment. As pointed out, we revised the label to indicate Itga8 protein rather than antibody. In the revised manuscript, the immunostaining data were added as panel b. Accordingly, the Itga8 protein data are now shown in Extended Data Fig. 4c.

20. There is inconsistent formatting across figure legends (e.g., some use full sentences while others use fragments).

[Response]

We appreciate the reviewer’s comment. In response, we revised the figure legends throughout the manuscript to ensure consistent formatting and style. Specifically, we unified the legends in a full-sentence format, including those in Figs. 1 and 7 and Extended Data Figs. 1 and 4.

21. Some panels aren't fully explained (especially in supplementary figures). For example, Extended Figure 5c-e: it is unclear which BMT is being referred to here.

[Response]

We appreciate the reviewer’s comment. To clarify this point, we revised the legend to explicitly indicate that the panels corresponding to the original Extended Figs. 5c–e (now new Extended Data Fig. 10c–e) refer to the BM transplantation experiments performed after Transwell coculture. We also revised other figure legends where needed to improve clarity.

22. Some abbreviations are used before being defined.

[Response]

We appreciate the reviewer’s comment. In response, we carefully checked the manuscript and revised the text so that abbreviations are defined at their first appearance in the main text, including PBS, DTR, Alcam, Sca-1, Gr-1, Flt3, and Cre.

23. Panel labels (A, B, C, etc.) aren't always clearly linked to their descriptions.

[Response]

We appreciate the reviewer’s comment. In response, we revised the figure legends and the corresponding descriptions in the main text to ensure that panel labels are clearly linked. We also corrected an incorrect figure reference in the main text, where “Extended Data Fig. 4f–h” should have been “Fig. 4f–h.”

24. Show representative flow plots, especially for ex vivo transwell coculture studies.

[Response]

We appreciate the reviewer’s comment. Representative flow cytometry plots from the direct coculture experiments are shown in Extended Data Fig. 9, and those from the ex vivo Transwell coculture experiments are shown in Extended Data Fig. 10.
