# Supplementary material for: Endosteal integrin-α8⁺ mesenchymal stem cells maintain haematopoietic stem cell function via extracellular matrix-mediated interactions
Source: Nat Commun. 2026 Jul 6;17:8355. doi: 10.1038/s41467-026-75276-2 (PMC13473570; doi:10.1038/s41467-026-75276-2)
Supplement: Supplementary file 3 [file 41467_2026_75276_MOESM3_ESM.pdf]

nature portfolio

Corresponding author(s): Fumio Arai

Last updated by author(s): Jun 1, 2026

Reporting Summary

Nature Portfolio wishes to improve the reproducibility of the work that we publish. This form provides structure for consistency and transparency in reporting. For further information on Nature Portfolio policies, see our Editorial Policies and the Editorial Policy Checklist.

Statistics

For all statistical analyses, confirm that the following items are present in the figure legend, table legend, main text, or Methods section.

n/a	Confirmed	
☐	☒	The exact sample size (*n*) for each experimental group/condition, given as a discrete number and unit of measurement
☐	☒	A statement on whether measurements were taken from distinct samples or whether the same sample was measured repeatedly
☐	☒	The statistical test(s) used AND whether they are one- or two-sided<br>*Only common tests should be described solely by name; describe more complex techniques in the Methods section.*
☒	☐	A description of all covariates tested
☒	☐	A description of any assumptions or corrections, such as tests of normality and adjustment for multiple comparisons
☐	☒	A full description of the statistical parameters including central tendency (e.g. means) or other basic estimates (e.g. regression coefficient) AND variation (e.g. standard deviation) or associated estimates of uncertainty (e.g. confidence intervals)
☐	☒	For null hypothesis testing, the test statistic (e.g. *F*, *t*, *r*) with confidence intervals, effect sizes, degrees of freedom and *P* value noted<br>*Give P values as exact values whenever suitable.*
☒	☐	For Bayesian analysis, information on the choice of priors and Markov chain Monte Carlo settings
☒	☐	For hierarchical and complex designs, identification of the appropriate level for tests and full reporting of outcomes
☒	☐	Estimates of effect sizes (e.g. Cohen's *d*, Pearson's *r*), indicating how they were calculated

Our web collection on statistics for biologists contains articles on many of the points above.

Software and code

Policy information about availability of computer code

Data collection	The data were acquired using the FACS Diva 8.0 software (BD Biosciences) on the BD FACS Aria Ⅲ and FACS Fusion instruments.<br>The data were acquired using ZEN 2012 SP5 software (Carl Zeiss) on an LSM 780 confocal microscope system.<br>The data were acquired using QuantStudio Real-Time PCR Software on a QuantStudio 3 Real-Time PCR System (Applied Biosystems).<br>The library was sequenced using the NextSeq 500 and NovaSeq 6000 system (Illumina)
Data analysis	Flow cytometric analysis was performed using FlowJo software (version 10.8, BD Biosciences).<br>Statistical analysis was performed using GraphPad Prism 8.4.3 (GraphPad Software).<br>The images were analysed using ZEN 3.3 blue edition (Zeiss) and ImageJ (NIH).<br>The analysis of bulk RNA sequencing data was conducted using the iDEP2.01 software (http://bioinformatics.sdstate.edu/idep/).<br>The analysis of single cell RNA sequencing data was conducted using the Loupe Browser v8.0.0 (10x Genomics) software.

For manuscripts utilizing custom algorithms or software that are central to the research but not yet described in published literature, software must be made available to editors and reviewers. We strongly encourage code deposition in a community repository (e.g. GitHub). See the Nature Portfolio guidelines for submitting code & software for further information.

Data

Policy information about availability of data

All manuscripts must include a data availability statement. This statement should provide the following information, where applicable:

- Accession codes, unique identifiers, or web links for publicly available datasets
- A description of any restrictions on data availability
- For clinical datasets or third party data, please ensure that the statement adheres to our policy

Bulk RNA sequencing data have been deposited in the Gene Expression Omnibus under accession number GSE280158.
Single cell RNA sequencing data have been deposited in the Gene Expression Omnibus under accession number GSE326153.

Research involving human participants, their data, or biological material

Policy information about studies with human participants or human data. See also policy information about sex, gender (identity/presentation), and sexual orientation and race, ethnicity and racism.

Reporting on sex and gender	N/A
Reporting on race, ethnicity, or other socially relevant groupings	N/A
Population characteristics	N/A
Recruitment	N/A
Ethics oversight	N/A

Note that full information on the approval of the study protocol must also be provided in the manuscript.

Field-specific reporting

Please select the one below that is the best fit for your research. If you are not sure, read the appropriate sections before making your selection.

☒ Life sciences ☐ Behavioural & social sciences ☐ Ecological, evolutionary & environmental sciences

For a reference copy of the document with all sections, see nature.com/documents/nr-reporting-summary-flat.pdf

Life sciences study design

All studies must disclose on these points even when the disclosure is negative.

Sample size	The sample size was determined based on pilot experiments and previous studies, with a minimum of three independent biological replicates per condition to ensure sufficient statistical power.
Data exclusions	The analysis did not result in the exclusion of any data points.
Replication	The key findings were replicated in at least two independent experimental batches.
Randomization	The random assignment of experiments was performed in both in vitro and in vivo contexts.
Blinding	As this study did not involve human participants and the readouts were primarily quantitative and objective, blinding of experimenters was not implemented.

Reporting for specific materials, systems and methods

We require information from authors about some types of materials, experimental systems and methods used in many studies. Here, indicate whether each material, system or method listed is relevant to your study. If you are not sure if a list item applies to your research, read the appropriate section before selecting a response.

Materials & experimental systems

n/a	Involved in the study
☐	☒ Antibodies
☒	☐ Eukaryotic cell lines
☒	☐ Palaeontology and archaeology
☐	☒ Animals and other organisms
☒	☐ Clinical data
☒	☐ Dual use research of concern
☒	☐ Plants

Methods

n/a	Involved in the study
☒	☐ ChIP-seq
☐	☒ Flow cytometry
☒	☐ MRI-based neuroimaging

Antibodies

Antibodies used

The following antibodies were used for flow cytometry: Anti-mouse CD45-FITC (Biolegend, Cat# 103108, Clone:30-F11, 1:200), Anti-mouse Ter119-PE (BD Biosciences, Cat# 553673, Clone:TER-119, 1:100), Anti-mouse CD31-PE-Cy7 (BD Biosciences, Cat# 561410, Clone:390, 1:100), Anti-mouse CD166(Alcam)-APC (Miltenyi biotec, Cat# 130-105-444, Clone:REA370, 10:100), Anti-mouse Itga8-Biotin (R&D, Cat# BAF4076, 2:100), Streptavidin-BV421 (BD Biosciences, Cat# 563259, 2:100), Streptavidin-APC-Cy7 (BD Biosciences, Cat# 554063, 2:100), Anti-mouse Ly-6A/E (Sca-1)-AF488 (Biolegend, Cat# 122516, Clone:E13-161.7, 1:100), Anti-mouse Ly-6A/E (Sca-1)-BV421 (BioLegend, Cat# 108127, Clone:D7, 2:100), Anti-mouse Ly-6A/E (Sca-1)-BV510 (BioLegend, Cat# 108129, Clone:D7, 3:100), Anti-mouse Ly-6A/E (Sca-1)-Biotin (Biolegend, Cat# 122504, Clone: E13-161.7, 2:100), Anti-mouse Itga8 (R&D, Cat# AF4076, 2:100), Anti-mouse CD51-APC (Bio-Rad, Cat# MCA2461A647, Clone:RMV-7, 5:100), Anti-mouse CD140a-BV421 (BD Biosciences, Cat# 562774, Clone:APA5, 3:100), Anti-mouse CD140a-Biotin (Biolegend, Cat# 135909, 2:100), LepR-Biotin (R&D, Cat# BAF497, 2:100), Anti-mouse CD4-PerCP-Cy5.5 (Biolegend, Cat# 100540, Clone:RM4-5, 1:200), Anti-mouse CD4-PE (BD Biosciences, Cat# 553049, Clone:RM4-5, 1:200), Anti-mouse CD8a-PerCP-Cy5.5 (Biolegend, Cat# 100734, Clone:53-6.7, 1:200), Anti-mouse CD8a-PE (BD Biosciences, Cat# 553033, Clone:53-6.7, 1:200), Anti-mouse CD45R/B220-PerCP-Cy5.5 (Biolegend, Cat# 103236, Clone:RA3-6B2, 1:200), Anti-mouse CD45R/B220-PE (BD Biosciences, Cat# 553090, Clone:RA3-6B2, 1:200), Anti-mouse Ter119-PerCP-Cy5.5 (Biolegend, Cat# 116228, Clone:TER-119, 1:200), Anti-mouse Ter119-PE (BD Biosciences, Cat# 553673, Clone:TER-119, 1:200), Anti-mouse Ter119-BV421 (Biolegend, Cat# 116234, Clone:TER-119, 2:100), Anti-mouse Granulocyte receptor 1 (Gr-1) (Ly-6G/Ly-6C)-PerCP-Cy5.5 (Biolegend, Cat# 116211, Clone:RB6-8C5, 1:200), Anti-mouse Gr-1(Ly-6G/Ly-6C)-PE (BD Biosciences, Cat# 561084, Clone:RB6-8C5, 1:200), Anti-mouse CD11b(Mac-1)-PerCP-Cy5.5 (Biolegend, Cat# 101228, Clone:M1/70, 1:200), Anti-mouse CD11b (Mac-1)-PE (BD Biosciences, Cat# 553311, Clone:M1/70, 1:200), Anti-mouse CD117(c-Kit)-APC (Biolegend, Cat# 105812, Clone:2B8, 5:100), CD117 MicroBeads, mouse (Miltenyi Biotec, Cat# 130-091-224, 10:100), Anti-mouse CD150(SLAM)-PE-Cy7 (Biolegend, Cat# 115914, Clone:TC15-12F12.2, 5:100), Anti-mouse CD48-BV510 (BD Biosciences, Cat# 563536, Clone:HN48-1, 3:100), Anti-mouse CD135 (FMS-like tyrosine kinase 3, Flt3)-BV421 (BD Biosciences, Cat# 562898, Clone:A2F10.1, 3:100), Anti-mouse CD34-Biotin (Thermo Fisher Scientific, Cat# 13-0341-85, 4:100), Anti-mouse CD16/32-APC-Cy7 (BD Biosciences, Cat# 553776, Clone:93, 2:100), Anti-mouse CD127(IL-7Ra)-PE-Cy7 (Biolegend, Cat# 101327, Clone:A7R34, 5:100), Anti-mouse CD45.1-PE (Tonbo Biosciences, Cat# 60-1271, Clone:A20, 1:100), Anti-mouse CD45.1-AF488 (Biolegend, Cat# 110718, Clone:A20, 1:100), Anti-mouse CD45.2-FITC (BD Biosciences, Cat# 553772, Clone:104, 1:100), Anti-mouse CD45.2-APC (BD Biosciences, Cat# 553776, Clone:104, 1:100), CD45 MicroBeads, mouse (Miltenyi Biotec, Cat# 130-052-301, 5:100), Anti-Ter-119 MicroBeads, mouse (Miltenyi Biotec, Cat# 130-049-901, 5:100), Anti-Annexin V-APC (BioLegend, Cat# 640920, 5:100). The Annexin V Binding Buffer (BioLegend, Cat# 422201) was used for Annexin V staining.
The antibodies used for immunohistochemical staining are listed below: Anti-mouse Alcam (GeneTex, Cat# GTX130098, 1:250), Anti-mouse CD45-APC (BD Biosciences, Cat# 561018, Clone:30-F11, 5:100), Anti-mouse CD4-APC (Biolegend, Cat# 100516, Clone:RM4-5, 2:100), Anti-mouse CD8a-APC (Biolegend, Cat# 100712, Clone:53-6.7, 2:100), Anti-mouse CD45R/B220-APC (Tonbo Biosciences, Cat# 20-0452, Clone:RA3-6B2, 2:100), Anti-mouse Ter119-APC (Biolegend, Cat# 116211, Clone:TER-119, 2:100), Anti-mouse Gr-1(Ly-6G/Ly-6C)-APC (BD Biosciences, Cat# 553129, Clone:RB6-8C5, 2:100), Anti-mouse CD11b(Mac-1)-APC (Biolegend, Cat# 101212, Clone:M1/70, 2:100), Anti-mouse CD150(SLAM)-PE (Biolegend, Cat# 115904, Clone:TC15-12F12.2, 5:100), Anti-mouse CD48-APC (Biolegend, Cat# 103412, Clone:HN48-1, 5:100), Anti-mouse CD41-APC (Biolegend, Cat# 133914, Clone:MWReg30, 2:100), Donkey anti-Goat IgG (H+L) Highly Cross-Adsorbed Secondary Antibody, Alexa Fluor™ Plus 405 (Thermo Fisher Scientific, Cat# A48259, 1:200), Donkey anti-Rabbit IgG (H+L) Highly Cross-Adsorbed Secondary Antibody, Alexa Fluor™ 488 (Thermo Fisher Scientific, Cat# A-21206, 1:200), Streptavidin, Alexa Fluor™ 555 Conjugate (Thermo Fisher Scientific, Cat# S32355, 1:200), MFAP4 Polyclonal Antibody (Thermo Fisher Scientific, Cat# PA5-42013, 1:250).

Validation

The antibodies utilized in this study are extensively employed within the field and have been validated by their respective manufacturers.

Animals and other research organisms

Policy information about studies involving animals; ARRIVE guidelines recommended for reporting animal research, and Sex and Gender in Research

Laboratory animals

The experimental subjects were six- to twelve-week-old mice. All mice were maintained in a controlled environment free of pathogens, with a 12-hour light cycle from 8:00 a.m. to 8:00 p.m., at temperatures ranging from 20 to 26°C, and humidity levels between 40 and 60%. The mice used in the experiments were 6 to 12 weeks old. C57BL/6JJcl (B6-Ly5.2) mice (6–8 weeks old) were obtained from CLEA Japan, Inc., and congenic C57BL/6 mice for the Ly5 locus (B6-Ly5.1) were purchased from Sankyo-Lab Service (Tsukuba, Japan). LepR-Cre knock-in mice were provided by Dr Yoshihiro Baba (The University of Kyushu, Japan), and Rosa26-loxP-stop-loxP-DTR-tdTomato mice were provided by Dr. Masashi Yanagisawa (University of Tsukuba, Japan). Itga8-Cre KI mice were generated on a C57BL/6J background using CRISPR/Cas9 gene editing, where a guide RNA targeting the mouse Itga8 gene, together with a donor vector containing the IRES-Cre cassette and Cas9 mRNA, were co-injected into fertilised C57BL/6J mouse eggs to insert

	the IRES-Cre cassette downstream of the stop codon, producing targeted KI offspring. Rosa-DTR-tdTomato mice express tdTomato and DTR in a Cre-inducible manner. Itga8Cre/+; Rosa-DTR-tdTomato mice were generated by crossing Itga8-Cre KI mice with Rosa-DTR-tdTomato mice.
Wild animals	The experiment did not use wild animals.
Reporting on sex	Both male and female mice were used in the experiments.
Field-collected samples	Samples collected in the field weren't used in this experiment.
Ethics oversight	This study was approved by the Animal Experiment Committee of Kyushu University. All experiments followed the rules for animal experiments at Kyushu University.

Note that full information on the approval of the study protocol must also be provided in the manuscript.

Plants

Seed stocks	N/A
Novel plant genotypes	N/A
Authentication	N/A

Flow Cytometry

Plots

Confirm that:

- [x] The axis labels state the marker and fluorochrome used (e.g. CD4-FITC).
- [x] The axis scales are clearly visible. Include numbers along axes only for bottom left plot of group (a 'group' is an analysis of identical markers).
- [x] All plots are contour plots with outliers or pseudocolor plots.
- [x] A numerical value for number of cells or percentage (with statistics) is provided.

Methodology

Sample preparation

The following is a detailed protocol for performing a flow cytometry analysis of bone marrow (BM) cells.:
The mouse must be euthanized humanely according to the approved animal protocol. Sterile harvesting of the femora, tibias, and iliac bones is also required. The BM is then extracted from the bones using a syringe filled with cold phosphate-buffered saline (PBS) and transferred into a 50-mL tube. Subsequently, the cell suspension should be filtered through a 70-µm cell strainer to eliminate any residual bone fragments or debris.

The following is a detailed protocol for performing a flow cytometry analysis of bone lining cells (BLCs):
The mouse must be euthanized humanely according to the approved animal protocol. Sterile harvesting of the femora, tibias, and iliac bones is also required. Femora, tibias and iliac bones were fragmented into small pieces (approximately 1 – 2 mm) after flushing out the BM cells. The bone fragments were incubated at 37°C for 90 min in collagenase solution containing type I collagenase (2.5 mg/mL, Worthington Biochemical), 20% fetal calf serum (FCS) and 250 units of DNase (Sigma) in DMEM. Subsequently, the cell suspension should be filtered through a 70-µm cell strainer to eliminate any residual bone fragments or debris.

The following is a detailed protocol for performing a flow cytometry analysis of CD51+CD140a+ MSCs:
The mouse must be euthanized humanely according to the approved animal protocol. Sterile harvesting of the femora and tibias bones is also required. BM flushed out from the femora and the tibia was digested with Accumax at 26°C for 60 min. Subsequently, the cell suspension should be filtered through a 70-µm cell strainer to eliminate any residual bone fragments or debris.

(The following is a common procedure for collecting the cells described above.)
The cells were lysed in ACK lysis buffer. The cells must then be washed in PBS. The cell suspension should then be subjected to centrifugation, after which the pellet should be resuspended in FACS buffer. The antibodies previously enumerated in the antibody list should be added to the cell suspension at the final concentration recommended by the manufacturer. The mixture should be mixed gently. Subsequently, the cell antibody mixture should be incubated in the dark on ice for a duration of 30 minutes. Subsequent to the incubation period, approximately 1 mL of cold FACS buffer should be added to the tube, after which the cells should be subjected to centrifugation. The upper layer must be extracted with caution, taking care not to disrupt the pellet. Subsequent to this initial step, the wash step is to be repeated once more in order to remove unbound

antibodies and thereby reduce background staining. Following the final wash, the cell pellet should be resuspended in 500 µl of cold FACS buffer. Propidium iodide (PI) was added to a final concentration of 1 µg/mL to discriminate dead cells. Prior to acquisition, the cell suspension should be filtered through a 40-µm filter to remove cell aggregates and debris.

Instrument	FACS Aria III (BD Biosciences)<br>FACS Aria Fusion (BD Biosciences)
Software	The data were acquired using the FACS Diva 8.0 software (BD Biosciences) on the BD FACS Aria Ⅲ and FACS Fusion instruments.<br>Flow cytometric analysis was performed using FlowJo software (version 10.8, BD Biosciences).
Cell population abundance	The purity of the sorted or analyzed cells was assessed based on their frequency and absolute numbers in control wild-type animals. This approach is consistent with previous reports that this population represents a very small fraction of total bone marrow. In each experiment, a control group was included to maintain consistent gating and sorting conditions.
Gating strategy	A detailed gating strategy for the flow cytometry analysis is shown in the representative figure, including the selection of live single cells, lineage gating, and further subdivision into the target cell population.

☒ Tick this box to confirm that a figure exemplifying the gating strategy is provided in the Supplementary Information.
