# Supplementary material for: Endosteal integrin-α8⁺ mesenchymal stem cells maintain haematopoietic stem cell function via extracellular matrix-mediated interactions
Source: Nat Commun. 2026 Jul 6;17:8355. doi: 10.1038/s41467-026-75276-2 (PMC13473570; doi:10.1038/s41467-026-75276-2)
Supplement: Supplementary file 4 [file 41467_2026_75276_MOESM4_ESM.zip › Source Data/Extended Data Figure 2/Extended Data Figure 2b.pdf]

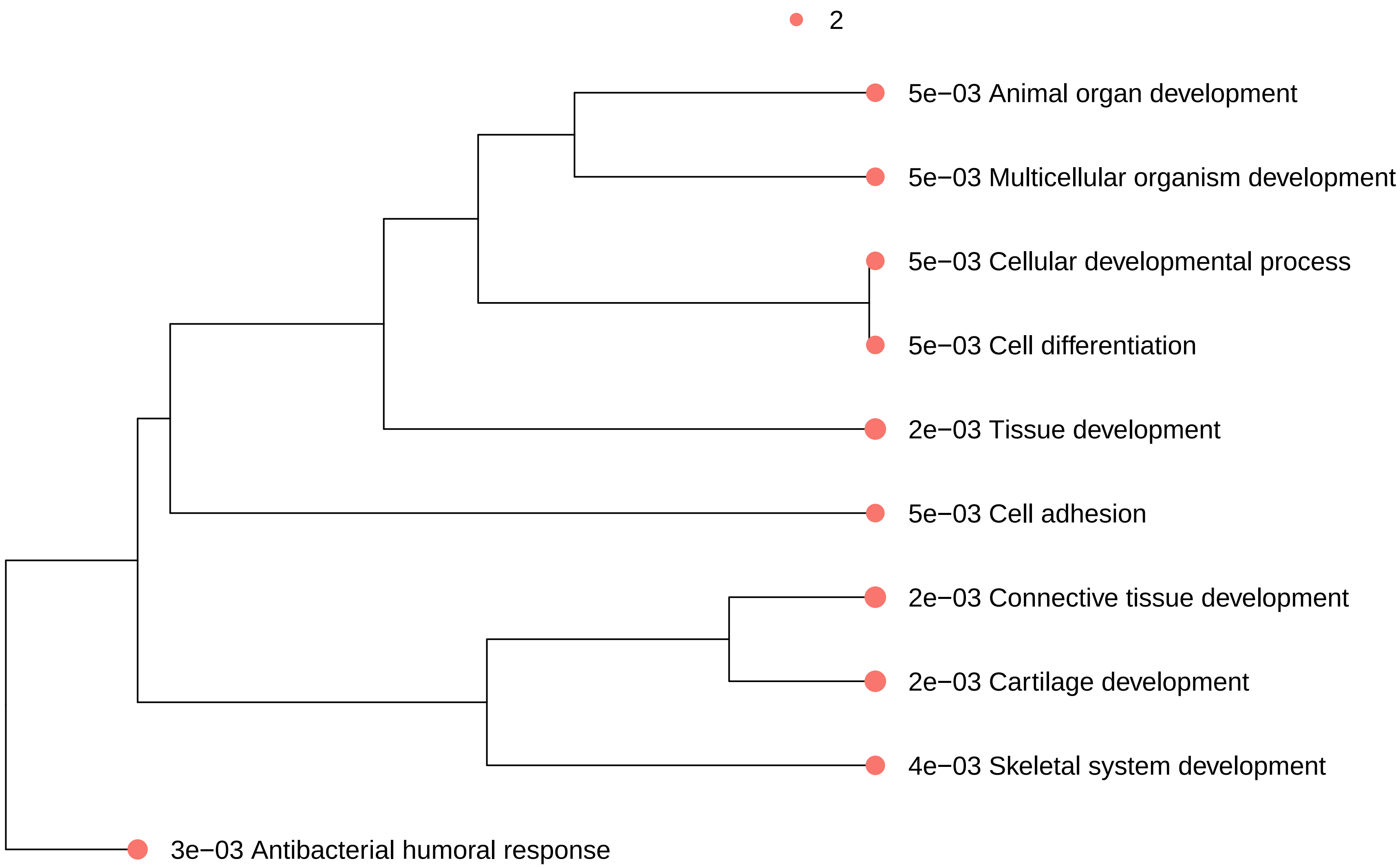
2
5e−03 Animal organ development
5e−03 Multicellular organism development
5e−03 Cellular developmental process
5e−03 Cell differentiation
2e−03 Tissue development
5e−03 Cell adhesion
2e−03 Connective tissue development
2e−03 Cartilage development
4e−03 Skeletal system development
3e−03 Antibacterial humoral response
