# Supplementary material for: Endosteal integrin-α8⁺ mesenchymal stem cells maintain haematopoietic stem cell function via extracellular matrix-mediated interactions
Source: Nat Commun. 2026 Jul 6;17:8355. doi: 10.1038/s41467-026-75276-2 (PMC13473570; doi:10.1038/s41467-026-75276-2)
Supplement: Supplementary file 4 [file 41467_2026_75276_MOESM4_ESM.zip › Source Data/Extended Data Figure 2/Extended Data Figure 2c.pdf]

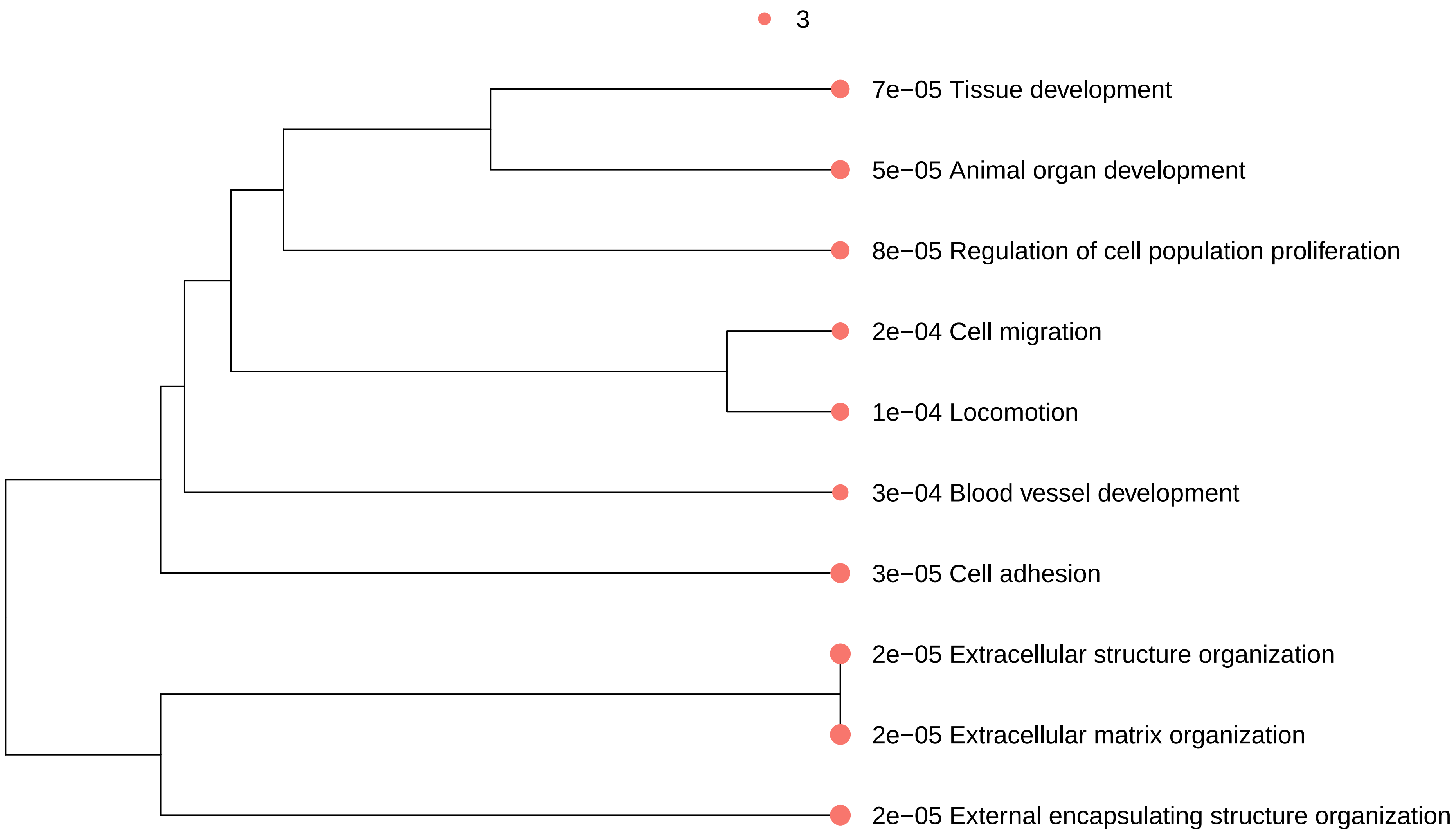
3
7e−05 Tissue development
5e−05 Animal organ development
8e−05 Regulation of cell population proliferation
2e−04 Cell migration
1e−04 Locomotion
3e−04 Blood vessel development
3e−05 Cell adhesion
2e−05 Extracellular structure organization
2e−05 Extracellular matrix organization
2e−05 External encapsulating structure organization
