# Supplementary material for: Endosteal integrin-α8⁺ mesenchymal stem cells maintain haematopoietic stem cell function via extracellular matrix-mediated interactions
Source: Nat Commun. 2026 Jul 6;17:8355. doi: 10.1038/s41467-026-75276-2 (PMC13473570; doi:10.1038/s41467-026-75276-2)
Supplement: Supplementary file 4 [file 41467_2026_75276_MOESM4_ESM.zip › Source Data/Extended Data Figure 2/Extended Data Figure 2d.pdf]

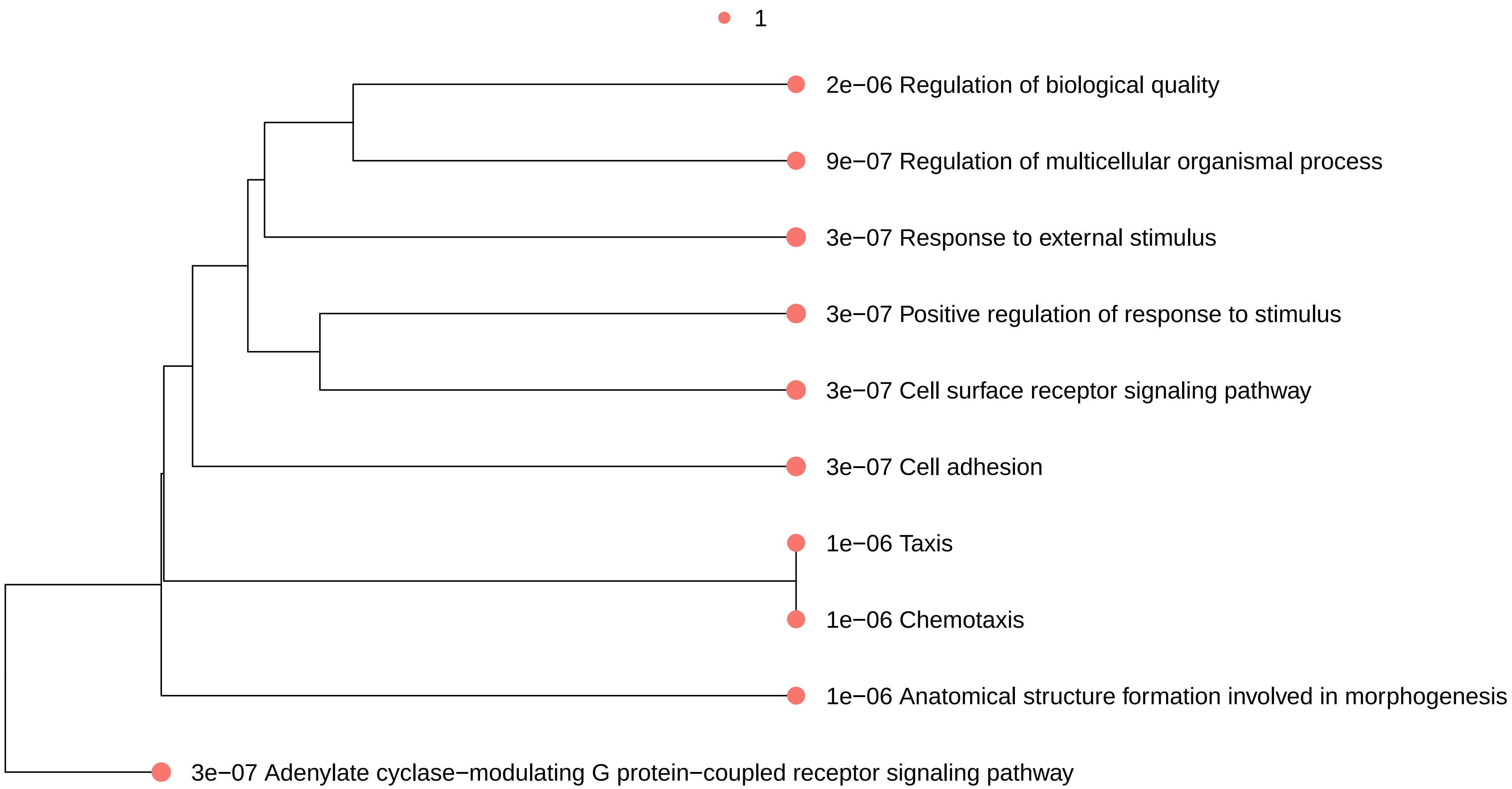
1
2e−06 Regulation of biological quality
9e−07 Regulation of multicellular organismal process
3e−07 Response to external stimulus
3e−07 Positive regulation of response to stimulus
3e−07 Cell surface receptor signaling pathway
3e−07 Cell adhesion
1e−06 Taxis
1e−06 Chemotaxis
1e−06 Anatomical structure formation involved in morphogenesis
3e−07 Adenylate cyclase−modulating G protein−coupled receptor signaling pathway
