# Supplementary material for: Endosteal integrin-α8⁺ mesenchymal stem cells maintain haematopoietic stem cell function via extracellular matrix-mediated interactions
Source: Nat Commun. 2026 Jul 6;17:8355. doi: 10.1038/s41467-026-75276-2 (PMC13473570; doi:10.1038/s41467-026-75276-2)
Supplement: Supplementary file 4 [file 41467_2026_75276_MOESM4_ESM.zip › Source Data/Extended Data Figure 2/Extended Data Figure 2e.pdf]

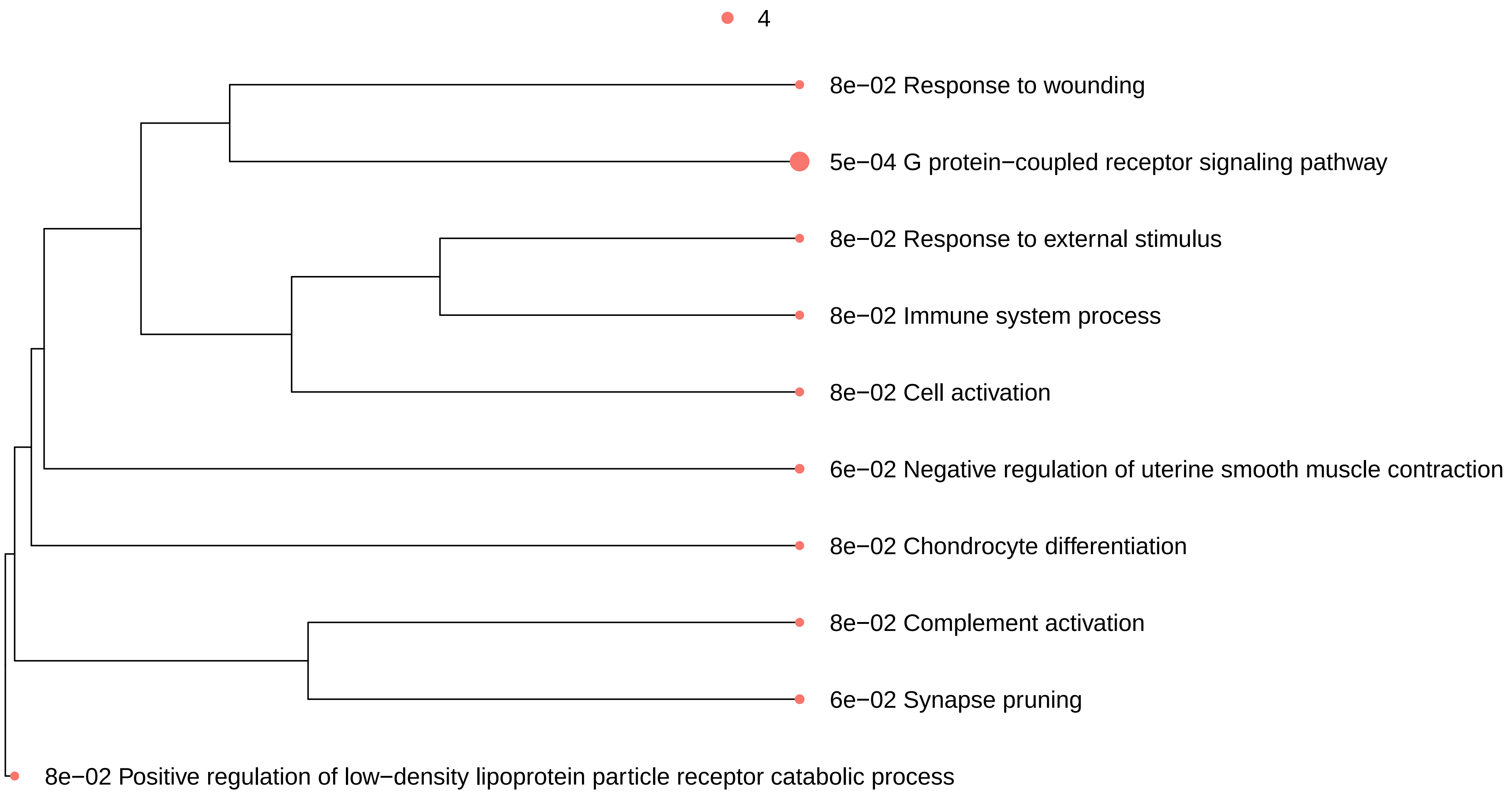
4
8e−02 Response to wounding
5e−04 G protein−coupled receptor signaling pathway
8e−02 Response to external stimulus
8e−02 Immune system process
8e−02 Cell activation
6e−02 Negative regulation of uterine smooth muscle contraction
8e−02 Chondrocyte differentiation
8e−02 Complement activation
6e−02 Synapse pruning
8e−02 Positive regulation of low−density lipoprotein particle receptor catabolic process
