# Supplementary material for: Endosteal integrin-α8⁺ mesenchymal stem cells maintain haematopoietic stem cell function via extracellular matrix-mediated interactions
Source: Nat Commun. 2026 Jul 6;17:8355. doi: 10.1038/s41467-026-75276-2 (PMC13473570; doi:10.1038/s41467-026-75276-2)
Supplement: Supplementary file 4 [file 41467_2026_75276_MOESM4_ESM.zip › Source Data/Extended Data Figure 2/Extended Data Figure 2f.pdf]

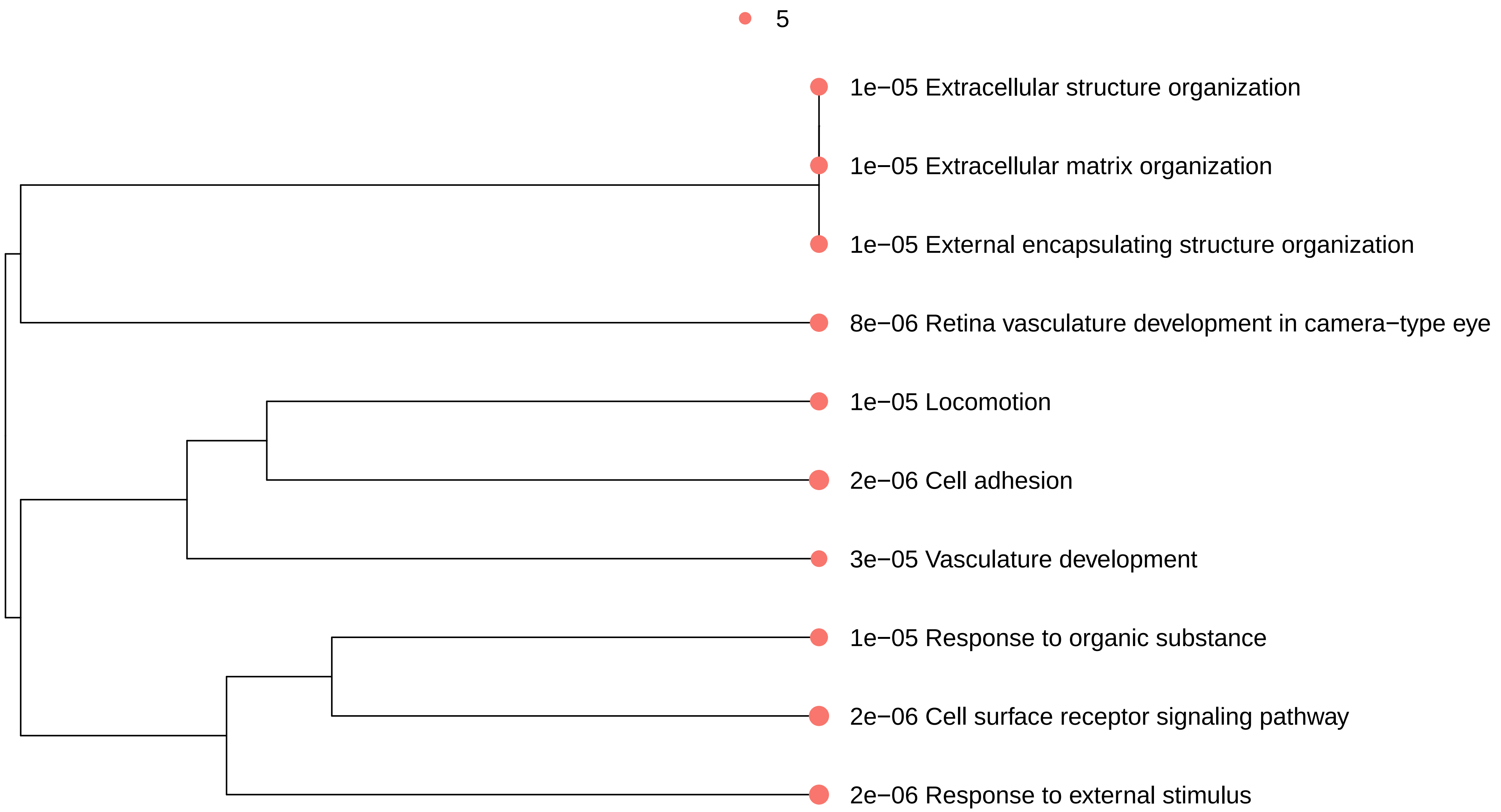
5
1e−05 Extracellular structure organization
1e−05 Extracellular matrix organization
1e−05 External encapsulating structure organization
8e−06 Retina vasculature development in camera−type eye
1e−05 Locomotion
2e−06 Cell adhesion
3e−05 Vasculature development
1e−05 Response to organic substance
2e−06 Cell surface receptor signaling pathway
2e−06 Response to external stimulus
