# Supplementary material for: Endosteal integrin-α8⁺ mesenchymal stem cells maintain haematopoietic stem cell function via extracellular matrix-mediated interactions
Source: Nat Commun. 2026 Jul 6;17:8355. doi: 10.1038/s41467-026-75276-2 (PMC13473570; doi:10.1038/s41467-026-75276-2)
Supplement: Supplementary file 4 [file 41467_2026_75276_MOESM4_ESM.zip › Source Data/Extended Data Figure 2/Extended Data Figure 2g.pdf]

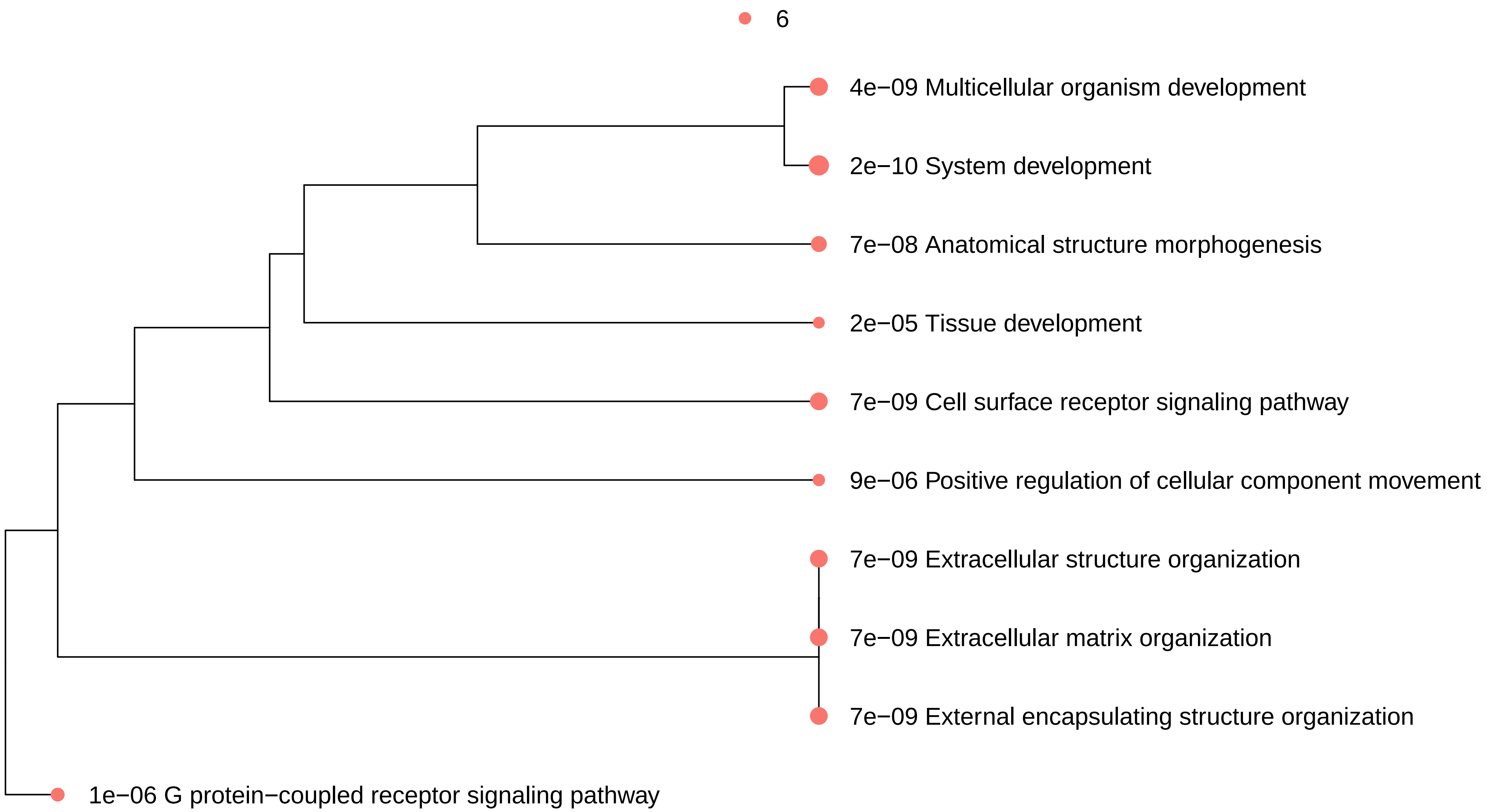
6
4e−09 Multicellular organism development
2e−10 System development
7e−08 Anatomical structure morphogenesis
2e−05 Tissue development
7e−09 Cell surface receptor signaling pathway
9e−06 Positive regulation of cellular component movement
7e−09 Extracellular structure organization
7e−09 Extracellular matrix organization
7e−09 External encapsulating structure organization
1e−06 G protein−coupled receptor signaling pathway
